# Evaluating the quality of large language model-generated preoperative patient education material: a comparative study across models and surgery types

**DOI:** 10.3389/fmed.2025.1701344

**Published:** 2025-12-11

**Authors:** Junwei Ma, Yunshan Zhang, Huifeng Tang, Xuemei Yi, Tangsheng Zhong, Xinyun Li, Gang Wang

**Affiliations:** 1 School of Nursing, Jilin University, Changchun, China; 2 The First Hospital of Jilin University, Changchun, China

**Keywords:** artificial intelligence (AI), large language model (LLM), preoperative education, patient education material (PEM), comparative analysis

## Abstract

**Background:**

Numerous studies have confirmed the effectiveness of large language models (LLMs) as a patient education tool; however, these studies primarily relied on the method of asking medical questions. So far, no studies have comprehensively assessed the quality of the complete preoperative patient education material (PEM) generated by LLMs from the perspectives of different models and surgical types.

**Objective:**

This study aims to comprehensively assess and compare the quality of different types of complete preoperative PEM generated by six common LLMs.

**Design:**

A Cross-sectional Comparative Study.

**Methods:**

We prompted 6 LLMs to generate preoperative PEMs for 6 distinct surgical types. For each surgical type, the materials were evaluated by 3 groups of experts from relevant fields using a 5-point scale for their accuracy and completeness. Two researchers assessed the materials for understandability and actionability using the PEMAT-P, and for suitability using SAM. We also analyzed the materials for readability with Flesch-Kincaid and for sentiment with the VADER sentiment analysis tool. Statistical analysis was performed using the Friedman test, followed by Conover’s *post-hoc* test with Bonferroni correction.

**Results:**

The research results show that each model has its strengths in different dimensions. All the models demonstrated excellent accuracy, understandability, and actionability with no statistically significant differences. In terms of completeness, Grok-4 and Claude-Opus-4 significantly outperformed GPT-4o. For suitability, Claude-Opus-4 performed the best, while Grok-4 was the worst. For readability, Grok-4 and Gemini-2.5-Pro were the easiest to understand, while Claude-Opus-4 had the lowest readability. Moreover, only Gemini-2.5-Pro could consistently generate content with positive emotions.

**Conclusion:**

The research has found that the materials generated by these models can achieve high levels in multiple dimensions, but there is no perfect model. These models can be used by medical staff to generate the initial draft of preoperative PEMs. However, before providing them to the patients, they still need to be reviewed and supplemented by the medical staff.

## 1 Introduction

Artificial intelligence (AI) is a multidisciplinary field that aims to simulate human intelligence through computer systems and perform tasks that were traditionally carried out by humans. Recently, AI can perform multiple tasks and generalize skills with minimal oversight (1, 2). A large language model (LLM) is an AI model based on deep learning, engineered for processing and generating natural language. With a vast number of parameters and a complex architecture, LLMs, through extensive training on massive text data, are capable of performing a wide range of natural language processing (NLP) tasks (3, 4). In November 2022, OpenAI released a dialog tool called ChatGPT, which is based on LLMs. This tool has received a huge response due to the appeal of its intuitive interface, natural-sounding responses, and open availability, marking a significant breakthrough in the field of NLP (1, 5). Subsequently, many companies launched their LLMs, for instance: Google’s Gemini series, Anthropic’s Claude, xAI’s Grok, Baidu’s ERNIE Bot, and DeepSeek’s DeepSeek.

These LLMs have demonstrated their transformative potential in numerous fields, such as healthcare. ChatGPT has proven capable of passing the three examinations of the US Medical Licensing Examination (USMLE), automatically generating hospital discharge summaries and post-operative patient guidance (6–8). In terms of patient education, LLMs can answer patients’ questions by converting complex medical information into plain and understandable language. Most studies have found that LLMs can provide accurate answers to patients’ questions. A research report stated that ChatGPT achieved an accuracy rate of 92.5% in answering questions about hypertension (9). Therefore, medical institutions not only can utilize them to enhance the level of personalized diagnosis and treatment, but also can assist patients in participating in medical decision-making (10, 11).

The preoperative patient education material (PEM) for patients serves as the core tool for preoperative education. They can help to explain the procedures and goals that aim to reduce perioperative anxiety, fatigue, and discomfort, and facilitate quicker recovery and discharge (12, 13). However, PEMs are expensive and time-consuming to develop and update (14). Therefore, the application of LLMs provides a new solution for preoperative education for surgical patients. Some studies claim that integrating LLMs into healthcare could fundamentally enhance the convenience for healthcare professionals and patients in accessing medical information (15). However, it is uncertain whether these LLMs can generate complete preoperative PEMs, and the reliability and accuracy of these materials have not yet been fully verified. In a review article, it is claimed that in various studies, there is a significant difference in the readability of the content generated by LLMs (16). Furthermore, although LLMs can provide conversational and seemingly authoritative answers to each complex medical question, they may also produce responses that appear coherent yet are factually erroneous or without substance. This phenomenon is known as “hallucination” (17–19).

Previous studies mainly involved collecting common medical questions and having LLMs generate responses, after which researchers would judge whether the generated answers were correct. This approach neglects the ability to generate comprehensive and multi-faceted preoperative PEMs. Moreover, it fails to take into account the patient’s medical and social background and other relevant information, thus resulting in limitations in the generated content. Therefore, this study aims to evaluate the ability of LLMs to generate complete preoperative PEM after obtaining more patient information. We will analyze the quality of these models in generating complete preoperative PEMs for different types of surgeries through the following seven dimensions: accuracy, completeness, understandability, actionability, suitability, readability, and sentiment. Through comparisons across various models and surgeries, this study seeks to facilitate the standardized application of LLMs in the field of patient preoperative education, providing a quantifiable decision-making basis for medical institutions to produce high-quality, readable, and personalized preoperative PEMs efficiently.

## 2 Materials and methods

### 2.1 Ethical approval

This study only utilized publicly available and anonymized data, and no interaction or intervention was conducted with any human subjects. The case information was sourced from the online medical forum DXY.CN. We carried out thorough de-identification procedures to ensure complete anonymity of the patients. Therefore, ethical approval was not required for this study.

### 2.2 Design and approach

**FIGURE 1:**
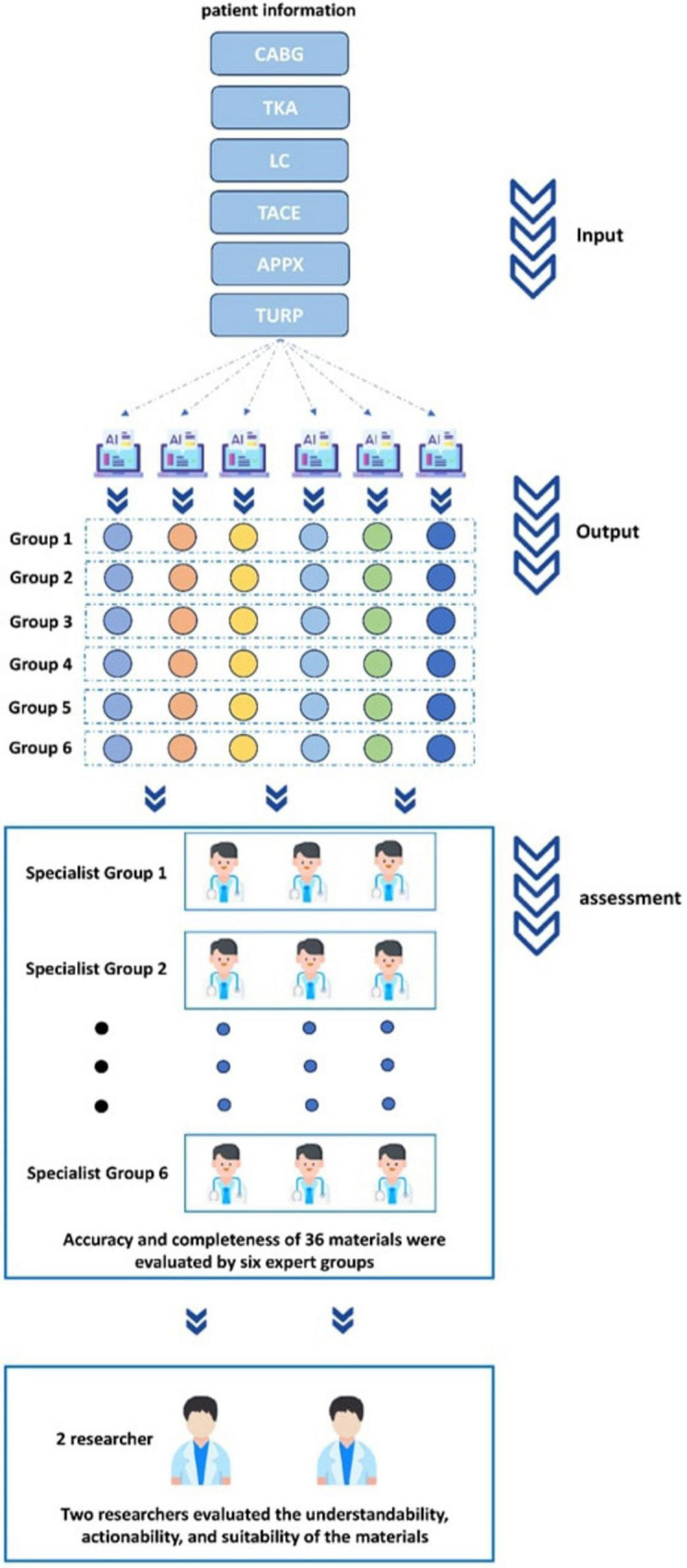
Flowchart of the overall study design.

This study adopted a descriptive comparative design to evaluate and compare the performance of 6 LLMs in generating complete preoperative PEMs (see the research flowchart in Figure 1). We selected the following LLMs based on their availability and wide usage: GPT-4o, Gemini-2.5-Pro, Claude-Opus-4, Grok-4, Ernie-4.5-Turbo, Deepseek-R1 (for details, Supplementary Table A.1). The patient information we used included the following surgical types: coronary artery bypass grafting (CABG), total knee arthroplasty (TKA), laparoscopic cholecystectomy (LC), transcatheter arterial chemoembolization (TACE), appendectomy (APPX), transurethral resection of the prostate (TURP) (for details, Supplementary Table A.2). The assessment of the generated preoperative PEMs focused on seven key performance dimensions: accuracy, completeness, understandability, actionability, suitability, readability, and Sentiment.

### 2.3 Data sources and collection

To prevent locally stored data from affecting LLM performance, before each interaction with the LLM, we will perform the following actions: close all sessions, clear the browser history (including cookies), and restart the computer. To ensure consistency across all trials, a single, definitive prompt was used. This prompt was presented to each LLM as the initial instruction: now, assume you are a medical professional, and your task is to provide comprehensive preoperative education for a patient about to undergo surgery. Please generate a complete preoperative education material based on the patient information and surgical details provided by me. Include all aspects of preoperative education and avoid using complex medical terms to ensure the readability of the material. And then input the patient’s case information to obtain the model’s response (The prompts and responses are provided in Supplementary Table A.3). All queries for this study were executed on 18 July 2025. Each model was assigned a number from 1 to 6 to identify it, and the evaluators did not know the identity information of the model. Each evaluator completed the scoring independently. To objectively measure the consistency among the scorers, we used Fleiss’ Kappa statistic. A Kappa statistic of ≤ 0 represents “no consistency,” > 0 but ≤ 0.20 represents “slight consistency,” 0.21–0.40 represents “fair consistency,” 0.41–0.60 represents “moderate consistency,” 0.61–0.80 represents “substantial consistency,” and > 0.80 represents “almost perfect consistency.”

### 2.4 Criteria of evaluation

#### 2.4.1 Accuracy and completeness

For each type of surgery, the accuracy and completeness of the PEMs were evaluated on a 5-point Likert scale by a panel of three specialists from the corresponding corresponding surgical specialty (e.g., cardiothoracic surgeons for CABG), each with over eight years of relevant clinical experience. The accuracy score ranged from 1 (completely inconsistent with the guidelines) to 5 (fully consistent and clinically accurate). The completeness score ranged from 1 (severe lack of key information) to 5 (covering all necessary information and having detailed content). For details, see Supplementary Tables A.4, A.5. Evaluations followed guidelines mainly from the American College of Surgeons, the National Health Service, the National Library of Medicine, UpToDate, among others. If there were disagreements among the assessors, consensus was reached via group discussion.

#### 2.4.2 Understandability and actionability

The Patient Education Materials Assessment Tool for Printable Materials (PEMAT-P) was used to evaluate the understandability and actionability of the materials generated by the LLMs (20). PEMAT-P consists of two dimensions: understandability and actionability. Since the materials generated by the models are purely text-based, questions related to visual aids, illustrations, tables, and lists were excluded. The understandability assesses whether the PEMs are expressed in a way that patients can understand, using a total percentage score of 11 true-or-false questions (questions 1–11). The actionability focuses on evaluating whether the PEMs can guide and motivate patients to take appropriate actions based on the provided information, using a total percentage score of 5 true-or-false questions (questions 20–24). For both the understandability and actionability indicators, a score of 70% or above is considered a qualified standard. Additionally, a higher PEMAT-P score (ranging from 0% to 100%) indicates a higher degree of comprehensibility and actionability. The assessment was conducted independently by 2 researchers with a medical background, and the differences were resolved through discussion. To calibrate the researchers’ assessment capabilities, the researchers received specialized training on the Patient Education Materials Assessment Tool (PEMAT) and User’s Guide.

#### 2.4.3 Suitability

The suitability of the preoperative PEMs was evaluated using the Suitability Assessment of Materials (SAM), which was developed by Doak et al. (21). SAM scale is a widely used and validated tool for evaluating health education materials, covering six dimensions: content, readability, visuals, design, motivation, and cultural relevance. After excluding the visuals and design dimensions, 14 items remained. Each item was scored on a 3-point scale (2 = superior, 1 = adequate, 0 = inadequate). The final percentage score was calculated, with 70%–100% being “superior,” 40%–69% being “adequate,” and 0%–39% being “inappropriate.” The suitability was also independently evaluated by 2 researchers with medical backgrounds, and the differences were resolved through discussion.

#### 2.4.4 Readability

The preoperative PEMs were, respectively, input into the WebFX readability tool.^1^ We reported two commonly used readability evaluation indicators: Flesch-Kincaid Reading Ease (FKRE) and Flesch-Kincaid Grade Level (FKGL). Both of these scores involve the total number of words, the number of sentences, and the number of syllables. The FKRE score ranges from 0 to 100, with higher scores indicating better readability and lower scores indicating higher complexity. FKGL can be used to assess the American school grade level required to understand the text; the lower the score, the better the readability.

#### 2.4.5 Sentiment

Sentiment of the preoperative PEMs was automatically evaluated using the VADER sentiment analysis tool. This tool can give the input text a composite score (ranging from −1 to +1), where a score > 0.05 indicates a positive emotion, a score < −0.05 indicates a negative emotion, and the range between these two represents neutrality. The sentiment score results are mainly used to assess whether the preoperative PEMs may trigger negative emotions in them.

### 2.5 Statistical analysis

The results were statistically analyzed using Python (version 3.10). For each performance dimension, we calculated the descriptive statistics of the results, including mean, standard deviation, median, variance, minimum, and maximum. Since the results were non-normal, we used the Friedman test to compare the differences between different models, and then conducted *post hoc* analysis using the Conover’s test. To control for the family-wise error rate (Type I error) resulting from multiple comparisons, the *p*-values from Conover’s test were adjusted using the Bonferroni correction method. The statistical significance level for all tests was set at *P* ≤ 0.05. In addition, we also conducted subgroup analyses by surgical type to examine whether there were any differences in the performance of LLMS in different types of surgeries.

## 3 Results

**FIGURE 2:**
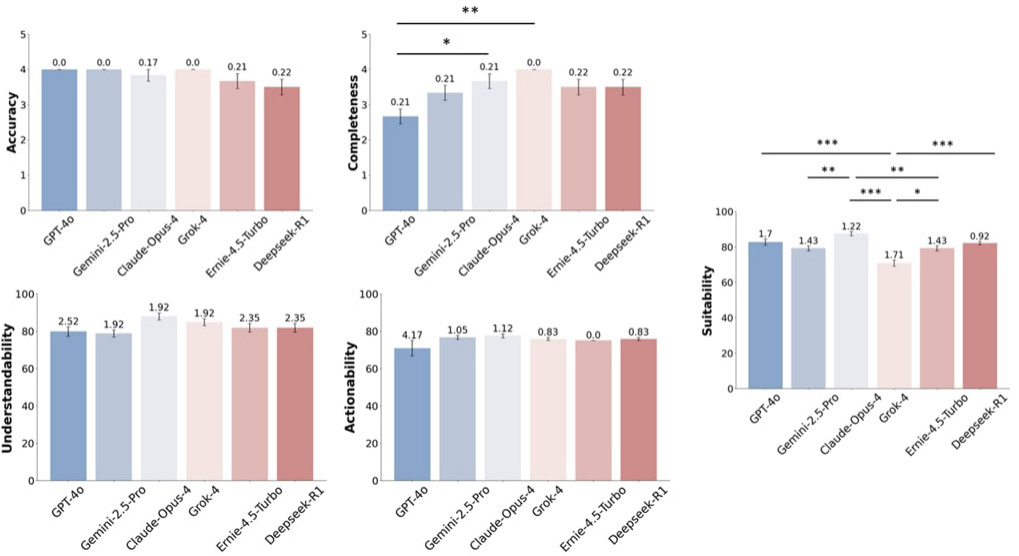
Quality assessment of PEMs generated by LLMs across 5 key dimensions. *p < 0.05, **p < 0.01, ***p < 0.001.

This study evaluated the quality of 36 preoperative PEMs generated by 6 LLMs. Before analyzing the results, in order to evaluate the inter-rater reliability of the subjective evaluation, we independently calculated the Fleiss’ Kappa coefficients for the two dimensions of accuracy and completeness. The Kappa coefficient for accuracy was 0.576, indicating moderate consistency, while for completeness, it was 0.692, indicating substantial consistency. Table 1 summarizes the descriptive statistics of the accuracy, completeness, understandability, actionability, and suitability of the 6 LLMs. Figure 2 presents a graphical summary of our comprehensive quality assessment across these 5 key dimensions.

**TABLE 1:** Descriptive statistics of LLMs on accuracy, completeness, understandability, actionability, and suitability.

Dimension and model	Mean	SD	Median	Variance	Min	Max	Friedman test [χ^2^(5), *P*]
Accuracy							χ^2^ = 10, *P* = 0.075
GPT-4o	4.00	0.00	4.0	0.00	4	4	
Gemini-2.5-Pro	4.00	0.00	4.0	0.00	4	4	
Claude-Opus-4	3.83	0.41	4.0	0.17	3	4	
Grok-4	4.00	0.00	4.0	0.00	4	4	
Ernie-4.5-Turbo	3.67	0.52	4.0	0.27	3	4	
Deepseek-R1	3.50	0.55	3.5	0.30	3	4	
Completeness							χ^2^ = 16.79, *P* = 0.005**
GPT-4o	2.67	0.52	3.0	0.27	2	3	
Gemini-2.5-Pro	3.33	0.52	3.0	0.27	3	4	
Claude-Opus-4	3.67	0.52	4.0	0.27	3	4	
Grok-4	4.00	0.00	4.0	0.00	4	4	
Ernie-4.5-Turbo	3.50	0.55	3.5	0.30	3	4	
Deepseek-R1	3.50	0.55	3.5	0.30	3	4	
Understandability							χ^2^ = 13.59, *P* = 0.018*
GPT-4o	79.80	6.16	77.78	37.96	72.73	90.91	
Gemini-2.5-Pro	78.79	4.70	81.82	22.04	72.73	81.82	
Claude-Opus-4	87.88	4.70	90.91	22.04	81.82	90.91	
Grok-4	84.85	4.70	81.82	22.04	81.82	90.91	
Ernie-4.5-Turbo	81.82	5.75	81.82	33.06	72.73	90.91	
Deepseek-R1	81.82	5.75	81.82	33.06	72.73	90.91	
Actionability							χ^2^ = 8.9560, *P* = 0.111
GPT-4o	70.83	10.21	75.0	104.17	50	75	
Gemini-2.5-Pro	76.67	2.58	75.0	6.67	75	80	
Claude-Opus-4	77.50	2.74	77.5	7.50	75	80	
Grok-4	75.83	2.04	75.0	4.17	75	80	
Ernie-4.5-Turbo	75.00	0.00	75.0	0.00	75	75	
Deepseek-R1	75.83	2.04	75.0	4.17	75	80	
Suitability							χ^2^ = 21.3433, *P* = 0.001***
GPT-4o	82.73	4.17	82.10	17.38	78.60	89.30	
Gemini-2.5-Pro	79.18	3.50	78.60	12.27	75.00	85.70	
Claude-Opus-4	87.50	3.01	89.30	9.07	82.10	89.30	
Grok-4	70.83	4.16	71.40	17.33	64.30	75.00	
Ernie-4.5-Turbo	79.15	3.49	80.35	12.17	75.00	82.10	
Deepseek-R1	82.12	2.25	82.10	5.04	78.60	85.70	

SD, standard deviation; Min, minimum; Max, maximum. *P < 0.05, **P < 0.01, ***P < 0.001.

### 3.1 Accuracy and completeness

As shown in Table 1, in terms of accuracy, GPT-4o, Gemini-1.5-Pro, and Grok-4 all performed equally well (Mean = 4.0, SD = 0), achieving high levels of accuracy and maintaining highly stable performance. Among the models, Deepseek-R1 performed the worst in terms of accuracy, with a mean score of 3.50, and had the highest variability among all the models (SD = 0.55). To assess whether there were statistically significant differences in the accuracy scores of each model, we conducted a Friedman test on the results. The result indicates that the overall differences among the models was not statistically significant [χ^2^(5) = 10, *P* = 0.075].

According to the descriptive statistics results, Grok-4 performed the best in terms of completeness (Mean = 4, SD = 0), followed by Claude-Opus-4 (Mean = 3.67, SD = 0.52). However, GPT-4o performed the worst (Mean = 2.67, SD = 0.52), indicating that the materials it generated had statistically significant deficiencies in completeness. The Friedman test results for the model’s completeness scores indicated statistically significant differences, χ^2^(5) = 16.79, *P* = 0.005. Further *post-hoc* tests employed the Conover’s test and were corrected using the Bonferroni method. The results showed that Grok-4 (*P* = 0.001) and Claude-Opus-4 (*P* = 0.042) scored statistically significantly higher in terms of completeness compared to GPT-4o.

### 3.2 Understandability and actionability

The preoperative PEMs generated by Claude-Opus-4 had the highest understandability (Mean = 87.88, SD = 4.70), followed by Grok-4 (Mean = 84.85, SD = 4.70). Gemini-2.5-Pro (Mean = 78.79, SD = 4.70) and GPT-4o (Mean = 79.80, SD = 6.16) had the lowest scores, but still met the qualified level (> 70%). The Friedman test’s assessment of the performance differences among the LLMs revealed that there were statistically significant differences in the understandability scores among the models [χ^2^(5) = 13.59, *P* = 0.018]. Subsequently, the Conover’s test with a Bonferroni correction did not indicate any statistically significant differences between any two of the models. This outcome might be due to the overly strict *post hoc* correction and the insufficient sample size.

The actionability assessment results indicated that the materials generated by all models are of similar actionability and all have reached the qualified level (> 70%). Among them, the best performance was shown by Claude-Opus-4 (Mean = 77.50, SD = 2.74), while the worst was GPT-4o (Mean = 70.83, SD = 10.21). The Friedman test, which assesses the performance differences between LLMs, showed that the actionability scores of the models did not show a statistically significant difference [χ^2^(5) = 8.9560, *P* = 0.111].

### 3.3 Suitability

For suitability, the best performer was Claude-Opus-4 (Mean = 87.50, SD = 3.01), while the worst performer was Grok-4 (Mean = 70.83, SD = 4.16), which still achieved a superior level (> 70%). The result of the Friedman test is χ^2^(5) = 21.3433, *P* = 0.001. The Conover’s test with a Bonferroni correction indicated that the suitability scores of Claude-Opus-4 were statistically significantly superior to Grok-4, Gemini-2.5-Pro, and Ernie-4.5-Turbo (all *P* < 0.005). Meanwhile, Grok-4’s suitability scores were statistically significantly inferior to GPT-4o, Claude-Opus-4, Ernie-4.5-Turbo, and Deepseek-R1 models (all *P* < 0.001).

### 3.4 Readability

**FIGURE 3:**
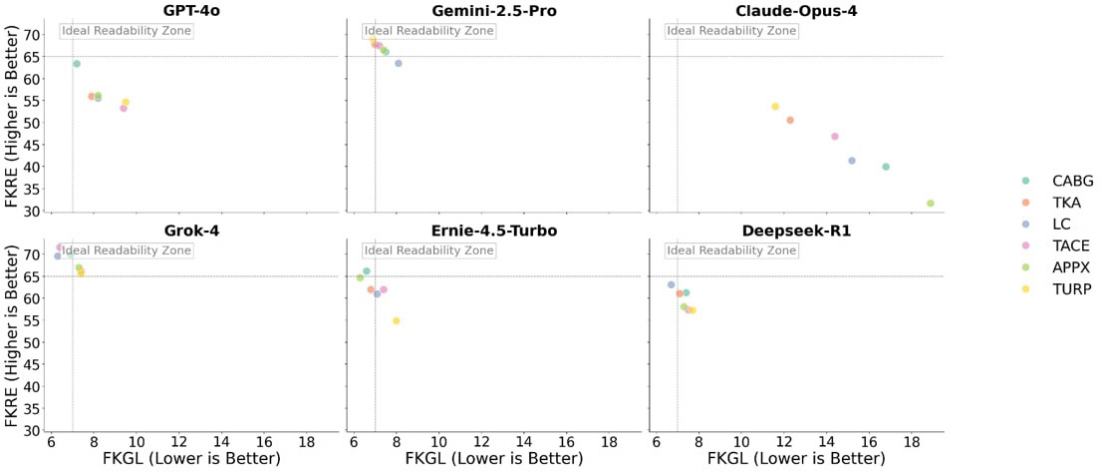
MMLs’ performance on readability: FKRE and FKGL.

The results showed that Grok-4 (FKRE: 65.6–71.50, FKGL: 6.3–7.4) and Gemini-2.5-Pro (FKRE: 63.4–69.0, FKGL: 6.9–8.1) perform the best, generating materials that are easily understandable by the general public. In contrast, The output content of Claude-Opus-4 is rather academic and complex, and is not suitable for readers who are not experts (FKRE: 31.6–53.6, FKGL: 11.6–18.9). Figure 3 shows the specific score results of the materials generated by each the LLMs. The Friedman test result for the FKRE score was χ^2^(5) = 26.95, *P* < 0.001. The Conover’s test results showed that the performance of Claude-Opus-4 was statistically significantly inferior to that of Deepseek-R1, Ernie-4.5-Turbo, Gemini-2.5-Pro, and Grok-4 (all *P* < 0.001). The Friedman test result for the FKGL score was χ^2^(5) = 20.3589, *P* = 0.001. The results of Conover’s test indicated that the FKGL score of Claude-Opus-4 was also statistically significantly higher than Deepseek-R1, Ernie-4.5-Turbo, Gemini-2.5-Pro, and Grok-4 (all *P* < 0.005).

### 3.5 Sentiment

**FIGURE 4:**
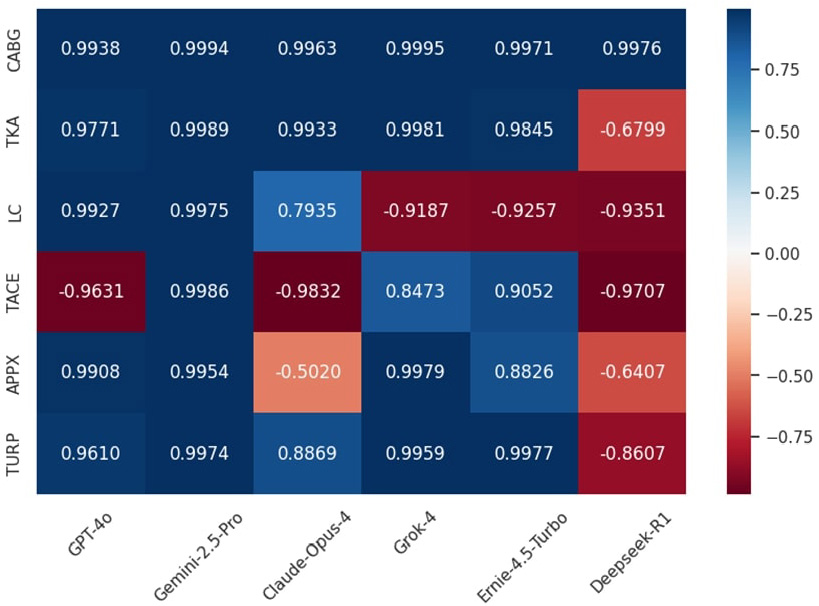
Heatmap of Sentiment Scores for LLM-Generated PEMs.

An emotional analysis was conducted to assess the emotional inclination of each LLM in all the surgeries, the results are presented in the form of a heatmap in Figure 4. The results indicated that Gemini-2.5-Pro is the only LLM that can provide positive emotions. We conducted a Friedman Test on the results generated by 6 LLMs, and the result was χ^2^(5) = 5.614, *P* = 0.345. The results of Conover’s test indicated that the sentiment score of GPT-4o was also statistically significantly higher than Claude-Opus-4, Ernie-4.5-Turbo, and Grok-4 (all *P* < 0.01).

### 3.6 Overall performance

**FIGURE 5:**
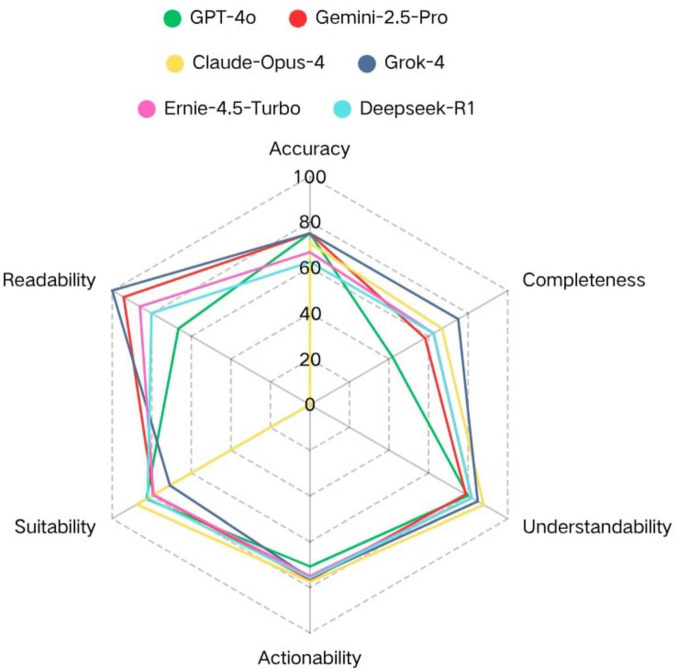
Multi-dimensional performance evaluation of six LLMs.

Figure 5 summarizes the overall performance of each model across all dimensions. The overall results indicated that each LLM can demonstrated high quality in most dimensions. Among them, the materials generated by Claude-Opus-4 had high accuracy, completeness, understandability, actionability, and suitability, but had the lowest score in readability. Grok-4 performed best in the completeness and readability of the content, but had the lowest score in suitability. GPT-4o excelled in accuracy, but performed the worst in the completeness and readability of the content. Gemini-2.5-Pro, Ernie-4.5-Turbo, and Deepseek-R1 had relatively balanced performance across all dimensions.

### 3.7 Subgroup analysis by surgery type

To investigate whether there are differences in the performance of LLMs when generating PEMs for different types of surgeries, we conducted subgroup analysis on six types of surgeries. We independently evaluated the differences in six types of surgeries across various dimensions using the Friedman Test, and the results showed that the LLMs performed quite robustly and consistently across most assessment dimensions, but there were significant differences in the understandability dimension among different types of surgeries (detailed descriptive statistics and results of the Friedman test are provided in Supplementary Table A.6 and Supplementary Figure A.1). No statistically significant differences were found among different types of surgeries in the dimensions of accuracy [χ^2^(5) = 8.18, *P* = 0.146], completeness [χ^2^(5) = 10.95, *P* = 0.052], actionability [χ^2^(5) = 7.5, *P* = 0.18], and suitability [χ^2^(5) = 5.614, *P* = 0.345]. However, there were statistically significant differences among different types of surgeries in the understandability dimension [χ^2^(5) = 11.47, *P* = 0.043]. Subsequently, the Conover’s test with Bonferroni correction indicated that the understandability score of TURP was statistically significantly better than that of CABG (*P* = 0.007).

Furthermore, the readability indicators calculated based on objective formulas, namely the FKRE [χ^2^(5) = 2.512, *P* = 0.775] and FKGL [χ^2^(5) = 2.308, *P* = 0.805], did not show significant differences among the six types of surgeries. In the dimension of sentiment, the Friedman test results for the six types of surgeries were χ^2^(5) = 16.57, *P* = 0.005. After Bonferroni correction, Conover’s test indicated that CABG surgery was significantly more emotionally positive than that for both LC and TACE surgeries (all *P* < 0.01).

## 4 Discussion

This study comprehensively evaluated and compared the quality of complete preoperative PEMs generated by common LLMs. The research results showed that these materials can all met the qualified standards (over 70%) in terms of understandability, actionability, and suitability, and can also reached a relatively high level in terms of accuracy and completeness. For readability, only Claude-Opus-4 and GPT-4o performed poorly. Through the assessment of the quality of preoperative PEMs, this study indicated that LLMs can be applied in the medical practice to help medical staff develop preoperative PEMs. However, we strongly recommend that they be subject to expert evaluation and further manual improvement before being disseminated to patients.

This study found that the preoperative PEMs generated by GPT-4o, Gemini-2.5-Pro, and Grok-4 all achieved a high level of accuracy. In addition, no serious errors that could endanger patient safety were found in all the materials. Only occasionally was some vague or outdated information provided. Most studies indicated LLMs provided accurate answers to patient inquiries (16, 22). A study also found that neither ChatGPT nor Google Bard made any errors or presented any dangerous information when answering common questions about obstructive sleep apnea (23). The results of this study represent an important first step in helping medical practitioners use LLMs as a source of preoperative PEMs. However, a key limitation was that the content generated by LLMs lacked evidence support. These materials do not provide references, which is consistent with previous observations (19). Considering the phenomenon that some LLMs often generate false references, the absence of references is not surprising.

The lack of completeness is a common problem with these materials. The PEMs generated by these models often lack some important parts, such as explanations of potential risks and complications. Moreover, specific details are occasionally overlooked; for instance, in the materials for CABG patients generated by these LLMs, it mentions preoperative smoking cessation, but fail to mentions preoperative alcohol cessation. These materials provide the time for removing chest drainage tubes and breathing tubes, but fail to mention the time for removing the urinary catheter. This “selective forgetting” of the models might be because the frequency of the text “smoking is harmful to health” in the training data is much higher than that of the specific phrase “preoperative alcohol abstinence,” thus failing to establish the correlation between the two in specific clinical scenarios. This further demonstrates the significance of human experts in reviewing and revising the PEMs generated by LLMs.

Studies have shown that low health literacy is correlated with deteriorating health status, and educational intervention can significantly improve patient prognosis (24, 25). Therefore, helping clinics create PEMs that are suitable for the American reading level (grades 7–8) might become a key factor influencing treatment outcomes (26, 27). In this study, Deepseek-R1, Ernie-4.5-Turbo, and Gemini-2.5-Pro were all able to generate content that is easily understandable for those who have reached the American junior high school graduation level. In previous studies, the content generated by the LLMs was mostly difficult to reach the reading level that junior high school students can understand (28). This change might be due to the continuous update and optimization of the algorithms and the repeated user feedback obtained through reinforcement learning. However, Claude-Opus-4 required a university or higher education level to understand the content. The high complexity of the content may limit its practical effect in patient education and consultation, highlighting the necessity for LLMs to further optimize the model to make the information more comprehensible to the general public (29, 30). A study utilized the preceding phrase “Please provide patient education materials at the level of a fifth-grade reader for the following questions” to prompt the system to generate PEMs that met the recommended fifth-grade reading level. The results showed that although it did not reach the fifth-grade reading level, there was still a significant improvement compared to responses without the preceding phrase (31). Another reason for not reaching a lower reading level might be that the readability assessment tools rely on vocabulary complexity and sentence length for analysis, and for medical materials using professional terms, the resulting readability score might be higher than the actual required true understanding level (32). Another drawback is that these LLMs generate only pure text and lack interactivity, which, to some extent, reduces the readability of these PEMs. However, with the rapid development of LLMs, we believe that in the future, these LLMs will be able to provide visual aids to improve readability.

Another finding was that currently, no model performs well across all dimensions. For instance, GPT-4o performed well on accuracy, but poorly on completeness. The high accuracy of GPT may stem from its lack of completeness; the text materials generated by GPT provide less information, so it makes fewer errors. Also, Claude-Opus-4 excelled in understandability, actionability, and suitability, but fell far behind other LLMs in readability. Given the limitations of a single model, perhaps improvements can be made through a model combination strategy, such as generating high-quality professional drafts using Claude-Opus-4 and then simplifying and improving readability using Grok-4 (33).

Grok-4 demonstrated a significant potential in customizing information based on individual needs and learning styles. The materials generated by Grok-4 have a strong personalized characteristic, such as providing corresponding post-operative home rehabilitation and work suggestions tailored to the patient’s occupation. The materials also provided specific recommendations for the patient’s underlying condition of diabetes. It mentioned that since the patient’s fasting blood sugar was 8.9 mmol/L, it was suggested that the patient adjust the medication or temporarily add insulin. This breaks through the limitation of traditional preoperative PEMs that cannot take into account each patient’s unique physiological conditions, life background, and cognitive level. However, this direct provision of medication suggestions has touched upon the core area of medical decision-making. This also raises concerns about the attribution of responsibility and ethics.

The subgroup analysis results of the six different surgical types have two important findings. Firstly, there is a significant disconnection between the subjective assessment of understandability and the objective readability indicators. Although there is no significant difference in the readability performance of all PEMs, the materials for TURP are significantly more understandable than those for CABG. The reason for this result may be that CABG involves highly abstract biomedical concepts, and explaining these concepts to patients requires more than just the use of simple vocabulary. In contrast, the patient education process for TURP is more programmatic, and the cognitive load of its concepts is relatively lower. This indicates that the superficial structure of the text (such as short sentences and common words) cannot guarantee that the deep medical concepts can be absorbed by patients. Therefore, when evaluating whether a text can truly be understood, the judgment of human experts is still indispensable. Additionally, LLMs shows significant differences in sentiment across different surgical types. This suggests that the output of LLMs are inherently embedded with the social and cultural biases in its training data. Since CABG is a major surgery aimed at saving lives or significantly improving quality of life, medical personnel strategically use positive and certain language when communicating to enhance patients’ confidence and alleviate anxiety. In contrast, LC is a more routine surgery, and its communication focus may be more on plain statements of efficiency and risks. This indicates that LLMs may appropriately convey warmth in some cases, but it may also introduce inappropriate optimism or pessimism when it is necessary to make an objective and neutral statement, thereby affecting patients’ informed decision-making.

In addition, there are still several issues that need to be addressed when using LLMs. For instance, the hallucination problem: LLMs may generate seemingly reasonable but factually incorrect or meaningless responses (34, 35). And the privacy issue: Both Google Bard and ChatGPT have explicitly stated that human AI trainers can access the generated conversation content, thus sensitive information should not be shared (31). LLMs also lack the capacity to take responsibility or abide by ethical and moral norms, so currently, their functions can only be limited to the “auxiliary tool” level (23). There are also other issues, such as legal liability and transparency (15, 36). Therefore, resolving these key issues plays a crucial role in the clinical application of LLMs.

This study has several limitations. Firstly, the assessment of the quality of PEMs may be influenced by reviewers’ subjective biases, also restricted by the small reviewer sample (3 reviewers for each material), all coming from the same institution. Secondly, this study only compares six LLMs’ generated PEMs, without comparing them with the PEMs written by surgeons. Therefore, there is no realistic reference for the quality of these materials. Thirdly, the assessment of the PEMs does not involve the participation of patients and lacks their real feedback. Fourthly, two-thirds of the LLMs used in this study require paid subscription services, which may limit their accessibility. Fifthly, the output of LLMs are highly variable, and this study does not test the consistency of the answers given by LLMs at different times. Moreover, the new version of the model is also being released frequently. These factors may affect the long-term relevance and applicability of the research conclusions. Future research should adopt a multi-iteration design to confirm the consistency of LLMs outputs. The next step should be to conduct a subsequent prospective study, where LLM-generated preoperative PEMs are directly compared with those written by medical teams through a larger and more diversified review panel in the real world. Patients should be invited to participate, and systematic evaluations of patients’ satisfaction, trust, and acceptance of AI-generated materials should be conducted. In addition, Future research should also systematically compare the general model with Med-LLM to explore whether Med-LLM can produce higher-quality PEMs.

## 5 Conclusion

This study evaluated and compared the quality of preoperative PEMs generated by various mainstream LLMs. The study revealed that these models could achieve a high level in multiple dimensions, but there was no perfect model. The most prominent limitation of these LLMs lies in their completeness, which needs further improvement. Currently, these models can serve as tools for medical staff to generate initial drafts of preoperative PEMs, helping medical staff effectively reduce the burden of writing standardized PEMs. Furthermore, we recommend the use of a model combination strategy, and before providing the materials generated by these models to the patients, they still need to undergo review and screening.

## Data Availability

The original contributions presented in this study are included in this article/Supplementary material, further inquiries can be directed to the corresponding author.

## Footnotes

1
https://www.webfx.com/tools/read-able/
