# Supplementary material for: Evaluating the quality of large language model-generated preoperative patient education material: a comparative study across models and surgery types
Source: Front Med (Lausanne). 2025 Dec 11;12:1701344. doi: 10.3389/fmed.2025.1701344 (PMC12738167; doi:10.3389/fmed.2025.1701344)
Supplement: Supplementary file 1 [file Supplementary_file_1.docx]

**supplementary materials**

[Table A.1: Basic Information of Large Language Models 2](#_Toc205226217)

[Table A.2: Patient information 2](#_Toc205226218)

[Table A.3: The preoperative education materials for patients generated by the large language model 11](#_Toc205226219)

[Table A.4: Accuracy - a 5-point Likert scale 143](#_Toc205226220)

[Table A.5: Completeness - a 5-point Likert scale 144](#_Toc205226221)

Table A.6: Subgroup analysis of PEM quality across

surgery types…………………………………………...………144

Figure A.1: Comparison of PEM scores across different

surgery types……………………..…………………………….147

Table A.1: Basic Information of Large Language Models

| Model Name | Developer | Headquarters |
| --- | --- | --- |
| GPT-4o | OpenAI | USA |
| Gemini-2.5-Pro | Google | USA |
| Claude-Opus-4 | Anthropic | USA |
| Grok-4 | xAI | USA |
| Ernie-4.5-Turbo | Baidu | China |
| Deepseek-R1 | DeepSeek AI | China |

Table A.2:Patient information

| 1.Case information: Patients scheduled for coronary artery bypass grafting (CABG) |
| --- |
| I. Basic Information  Name: Wang M.  Gender: Male  Age: 68 years old  Admission date: July 10th, 2025  Ethnicity: Han Chinese  Occupation: Retired engineer  Marital status: Married  Place of residence: A certain city  II. Chief Complaint  Recurrent pain behind the sternum for 2 years, aggravated with shortness of breath for 3 months.  III. Present Illness  The patient began to experience a squeezing pain behind the sternum without obvious inducement two years ago, radiating to the left shoulder and left arm, each episode lasting about 5 to 10 minutes, which could be relieved after rest and was effectively treated with sublingual nitroglycerin. The pain was often triggered by physical activity or emotional excitement. In the past three months, the frequency of chest pain attacks has increased, and it can occur even at rest, accompanied by exertional shortness of breath and one episode of nocturnal paroxysmal dyspnea (PND). An electrocardiogram performed at an outside hospital indicated ST-segment depression, while troponin levels were normal. Seeking further diagnosis and treatment, the patient came to our hospital. After admission, comprehensive examinations were completed, and coronary artery bypass grafting is planned.  IV. Past Medical History  Hypertension history: 15 years, with the highest blood pressure reaching 180/100 mmHg. Long-term oral antihypertensive drugs have been taken, but blood pressure control is not satisfactory.  History of type 2 diabetes: 10 years. Long-term oral hypoglycemic drugs have been taken, and blood sugar control is average.  History of hyperlipidemia: 5 years, no regular medication.  Smoking history: 40 years, averaging 20 cigarettes per day. Quit smoking one year ago.  History of alcohol consumption: Occasional drinking.  Allergy history: No known drug or food allergies.  Surgical history: None.  Family history: The patient's father had a history of coronary heart disease and died of myocardial infarction at the age of 58. The patient's mother has a history of hypertension and diabetes.  V. Physical Examination  T: 36.5℃ P: 72 beats/min R: 18 breaths/min BP: 145/88 mmHg  General condition: Conscious, in fair spirits, cooperative.  Skin and mucous membranes: No icterus, no rash, no petechiae.  Superficial lymph nodes: No enlargement was palpated.  Head: Normal development, no deformity.  Neck: No distension of the jugular vein, no enlargement of the thyroid gland.  Chest: Symmetrical thorax, clear breath sounds in both lungs, no dry or moist rales heard.  The precordial area shows no bulge. The apical impulse is located 0.5 cm inside the left midclavicular line at the fifth intercostal space. The cardiac silhouette is not enlarged. The heart rate is 72 beats per minute, and the rhythm is regular. No pathological murmurs are heard in the auscultation areas of each valve.  Abdomen: The abdomen is flat and soft, with no tenderness or rebound tenderness. The liver and spleen are not palpable below the costal margin. Bowel sounds are normal.  Spine and extremities: No deformity, normal movement, no edema in both lower extremities.  Nervous system: Physiological reflexes are present, pathological reflexes are not elicited.  VI. Auxiliary Examinations (Main Examinations after Admission)  Blood routine: WBC 7.5x10^9/L, Hb 130 g/L, Plt 250x10^9/L  Urinalysis: Proteinuria (+), glucosuria (+)  Comprehensive Biochemical Panel:  Fasting blood glucose: 8.9 mmol/L  Glycated hemoglobin (HbA1c): 7.8%  Total cholesterol: 5.8 mmol/L  Triglycerides: 2.6 mmol/L  Low-density lipoprotein cholesterol (LDL-C): 3.5 mmol/L  Creatinine: 85 umol/L  Liver function: Normal  Electrocardiogram (ECG): Sinus rhythm, ST segment depression of 0.1 mV in leads V4-V6.  Echocardiogram: Left ventricular ejection fraction (LVEF) 55%. Segmental hypokinesia of the left ventricular wall (anterior wall, anterior septum).  Coronary Angiography (CAG):  No significant stenosis was observed in the left main coronary artery (LM).  There is diffuse stenosis of 90% in the middle segment of the left anterior descending artery (LAD).  The proximal segment of the left circumflex artery (LCX) has a 75% stenosis.  The mid-segment of the right coronary artery (RCA) is 95% stenosed and the distal segment is occluded.  (Conclusion: Three-vessel disease, RCA is a chronic total occlusion lesion)  VII. Diagnosis  Coronary atherosclerotic heart disease  Unstable angina pectoris  Multivessel disease (LAD, LCX, RCA)  Grade 2 hypertension  Type 2 diabetes  Hyperlipidemia |
| 2.Case information: Patients scheduled for total knee arthroplasty (TKA) |
| I. Basic Information  Name: Li M.  Gender: Female  Age: 72 years old  Admission date: July 15, 2025  Ethnicity: Han Chinese  Occupation: Retired teacher  Marital status: Married  Place of residence: A certain city  II. Chief Complaint  Pain, deformity and limited mobility in the right knee joint for over 10 years, with aggravation and limping for 1 year.  III. Present Illness  The patient began to experience dull pain in the right knee joint without obvious cause over ten years ago. The pain worsened after activity and could be relieved by rest. At that time, it was not given much attention. In the past five years, the pain has gradually intensified, making it difficult to go up and down stairs and squat and stand. The walking distance has significantly shortened, and varus deformity of the right knee (O-shaped leg) has occurred. In the past year, the pain has continued to worsen, affecting sleep at night. The patient needs to use a cane to walk and has a limping gait, which seriously affects daily life. The patient has received treatments such as oral non-steroidal anti-inflammatory drugs, local physical therapy, and intra-articular injection of sodium hyaluronate, but the results were not satisfactory. To completely solve the pain and functional impairment of the knee joint, the patient came to our hospital for treatment. After admission, relevant examinations were completed, and total knee arthroplasty of the right knee joint is planned.  IV. Past Medical History  History of hypertension: 8 years. Oral antihypertensive drugs are taken and blood pressure is well controlled.  History of osteoporosis: 5 years. Regularly taking calcium tablets and vitamin D, and having regular bone density tests.  The patient denies a history of diabetes and coronary heart disease.  Allergy history: Allergic to penicillin (manifested as rash).  Surgical history: None.  Family history: Denies any special genetic disease history.  V. Physical Examination  T: 36.8℃ P: 78 beats/min R: 18 breaths/min BP: 130/80 mmHg  General condition: Conscious, in good spirits, with moderate nutrition.  Right knee joint:  Appearance: Obvious varus deformity (O-shaped legs), no obvious swelling of the knee joint, no local redness or heat. The patella has a fair range of motion.  Palpation: There is obvious tenderness at the edge of the patella, the joint space, the femoral condyle, and the tibial plateau.  Range of motion: Flexion range of motion is approximately 0-90° (normal is approximately 0-140°), extension is limited, and there is pain at the end of flexion.  Stability: The collateral ligaments and cruciate ligaments were basically stable upon examination.  Quadriceps femoris: There is some atrophy.  Gait: Limping gait.  Left knee joint: No swelling or tenderness, normal range of motion.  VI. Auxiliary Examinations (Main Examinations after Admission)  Blood routine: WBC 6.8x10^9/L, Hb 125 g/L, Plt 280x10^9/L  Urinalysis: Normal  Comprehensive Biochemical Panel:  Fasting blood glucose: 5.6 mmol/L  Liver and kidney functions: Normal  Electrolyte: Normal  Coagulation function: Normal  Electrocardiogram (ECG): Sinus rhythm, normal ECG.  Chest X-ray: Both lungs are clear, and the size of the cardiac shadow is normal.  Anteroposterior and lateral X-ray films of the right knee joint and weight-bearing position:  The joint space is narrowed (the medial space disappears).  Articular surface sclerosis and osteophyte formation.  Subchondral bone cysts of the femoral condyles and tibial plateau.  Obvious varus deformity, increased femoral-tibial angle.  Conclusion: Severe osteoarthritis of the right knee joint, genu varum deformity.  VII. Diagnosis  Osteoarthritis of the right knee joint (Kellgren-Lawrence grade: IV)  Inward deformity of the right knee joint  Osteoporosis  Hypertension |
| 3.Case information: Patients scheduled for laparoscopic cholecystectomy (LC) |
| I. Basic Information  Name: Zhang M.  Gender: Female  Age: 45 years old  Admission date: July 14, 2025  Ethnicity: Han ethnicity  Occupation: Accountant  Marital status: Married  Residence: A certain city in central China  II. Chief Complaint  Recurrent pain in the right upper abdomen has occurred for 2 years, accompanied by nausea and vomiting. The symptoms have worsened for 3 days.  III. Present Illness  Two years ago, the patient began to experience intermittent paroxysmal colic pain in the right upper abdomen without any obvious trigger. Each episode lasted from several minutes to several hours. It was often triggered by consuming greasy food. The patient also experienced nausea and vomiting of gastric contents. There was no fever or jaundice. An abdominal B-ultrasound examination at an external hospital indicated gallbladder stones. At that time, the symptoms were controllable and no surgical treatment was performed. In the past three days, the above symptoms have recurred and worsened. The pain is severe and does not subside. The patient also has obvious nausea and vomiting, with the vomitus being gastric contents. There is no fever, no chills, and no jaundice of the skin and sclera. To seek further diagnosis and treatment, the patient came to our emergency department. The emergency department completed relevant examinations and considered an acute attack of cholecystitis. The patient was admitted to the hospital for surgical treatment.  IV. Past Medical History  History of hypertension: No history.  Diabetes history: Denial.  History of chronic diseases: No.  Allergy history: No known history of drug or food allergies.  Surgical history: No history.  Family history: The mother has a history of gallbladder stones.  V. Physical Examination  T: 37.0℃ P: 85 beats/min R: 18 breaths/min BP: 125/75 mmHg  General condition: Conscious, with normal mental state and good cooperation.  Skin and sclera: No yellowing.  Abdomen: The abdomen is soft. There is obvious tenderness in the right upper abdomen, without rebound tenderness or muscle tension. The Murphy sign is positive. No mass is felt. No palpable liver or spleen below the ribs. The bowel sounds are normal.  Other system checks: No obvious abnormalities were found.  VI. Supplementary Examinations (Main Examinations after Admission)  Blood routine: WBC 10.5x10^9/L (neutrophil ratio 78%), Hb 135 g/L, Plt 260x10^9/L.  Urinalysis: Normal.  Liver function: Total bilirubin 15 umol/L, Direct bilirubin 4 umol/L, ALT 30 U/L, AST 25 U/L.  Blood amylase and lipase: Normal.  Electrocardiogram (ECG): Sinus rhythm, normal electrocardiogram.  Chest X-ray: Both lungs are clear, and the size of the heart is normal.  Abdominal ultrasound:  The gallbladder is enlarged, with a wall thickness of approximately 0.5 cm. Multiple strong echo masses can be seen in the cavity, the largest of which is about 1.5 cm, accompanied by posterior shadows.  The inner diameter of the common bile duct is approximately 0.6 cm, and no obvious dilation or stones are observed.  No obvious abnormalities were observed in the liver, pancreas, and spleen.  (Conclusion: Multiple gallbladder stones accompanied by acute cholecystitis)  VII. Diagnosis  Gallbladder stones  Acute cholecystitis |
| 4.Case information: Patients scheduled for transcatheter arterial chemoembolization (TACE) |
| I. Basic Information  Name: Ma M.  Gender: Male  Age: 62 years old  Admission date: July 05, 2025  Ethnicity: Han ethnicity  Occupation: Retired Worker  Marital status: Married  Residence: A certain city western China  II. Chief Complaint  Abdominal distension and pain have persisted for half a year, and have worsened over the past month, accompanied by fatigue and poor appetite.  III. Present Illness History  The patient began experiencing upper abdominal distension and pain half a year ago, without any obvious cause. The pain was persistent and dull, without any radiation. At that time, no attention was paid to it. In the past month, the upper abdominal pain has worsened, accompanied by significant fatigue, loss of appetite (nausea), and a weight loss of approximately 5 kilograms. There was no fever, jaundice, or nausea or vomiting. The patient had not achieved any effect from self-administered stomach medications. To clarify the diagnosis and for treatment, he came to our hospital for consultation. After admission, an abdominal CT examination revealed a liver lesion, and further liver biopsy confirmed hepatocellular carcinoma. Considering the patient's condition, TACE treatment was planned.  IV. Past Medical History  History of hepatitis B: 20 years, without regular antiviral treatment.  History of liver cirrhosis: 5 years. The diagnosis is clear and there is no obvious portal hypertension.  History of hypertension: No history.  Diabetes history: Denial.  Smoking history: 30 years, averaging 1 pack per day. Has been smoke-free for 2 years.  Alcohol consumption history: Denial.  Allergy history: No known history of drug or food allergies.  Surgical history: No history.  Family history: The father has a history of liver cancer.  V. Physical Examination  T: 36.6℃ P: 75 beats/min R: 18 breaths/min BP: 128/78 mmHg  General condition: Conscious, energetic, moderate nutrition, emaciated appearance.  Skin and sclera: No yellowing.  Abdomen: The abdomen is flat and soft. There is mild tenderness in the right upper abdomen, but no rebound tenderness or muscle tension. The liver can be felt below the ribs, with a firm texture and a blunt edge. The spleen is not palpable below the ribs. There are no signs of ascites. The bowel sounds are normal.  Both lower extremities: No edema.  Other system checks: No obvious abnormalities were found.  VI. Supplementary Examinations (Main Examinations after Admission)  Blood routine: WBC 6.5 x 10^9/L, Hb 110 g/L, Plt 120 x 10^9/L  Liver function:  Total bilirubin: 20 umol/L  Direct bilirubin: 6 umol/L  ALT: 70 U/L  AST: 85 U/L  Albumin: 35 g/L  Kidney function: Normal.  Coagulation function: International Normalized Ratio (INR) 1.2.  Alpha-fetoprotein (AFP): 2500 ng/mL  Hepatitis B virus markers: HBsAg (+), HBeAg (-), Anti-HBe (+), Anti-HBc (+), HBV-DNA 1.0x10^4 IU/mL.  Electrocardiogram (ECG): Sinus rhythm, normal electrocardiogram.  Chest X-ray: Both lungs are clear, and the size of the heart is normal.  Enhanced CT scan of the upper abdomen:  An irregular mass shadow can be observed in the S7 segment of the liver, with a size of approximately 5.0cm x 4.5cm. The enhanced scan shows a rapid entry and exit pattern, and it clears up in the delayed phase.  The liver shows diffuse changes, consistent with the manifestations of liver cirrhosis.  No clear tumor thrombus was observed in the main portal vein and its branches.  No obvious enlarged lymph nodes were observed in the abdominal cavity.  (Conclusion: A space-occupying lesion in the S7 segment of the liver, suspected to be hepatocellular carcinoma; liver cirrhosis)  Liver biopsy: The pathological result indicates hepatocellular carcinoma.  VII. Diagnosis  Hepatocellular carcinoma (HCC) (Barcelona Clinic Liver Cancer Staging BCLC: Stage B)  Hepatic cirrhosis related to hepatitis B (Child-Pugh grade A)  Chronic viral hepatitis B |
| 5.Case information: Patients scheduled for appendectomy |
| I. Basic Information  Name: Chen M.  Gender: Male  Age: 23 years old  Admission date: July 19, 2025  Ethnicity: Han ethnicity  Occupation: University student  Marital status: Unmarried  Residence: A certain city in central China  II. Chief Complaint  Severe right lower abdominal pain for 12 hours.  III. Present Illness  The patient experienced sudden upper abdominal pain without any obvious cause 12 hours before admission (approximately 21:00 last night). The pain was characterized as a persistent dull ache, accompanied by nausea, without vomiting, fever, or diarrhea. About 6 hours later (approximately 03:00 this morning), the pain gradually shifted to the right lower abdomen, changing to a persistent distending pain, which gradually intensified. The pain worsened with coughing or movement. There was still nausea, but no vomiting. Bowel movements were normal. There was no frequent urination, urgency, or pain during urination. In order to seek further treatment, the patient came to our hospital's emergency department. Emergency examinations were conducted, and acute appendicitis was considered. The patient was admitted to the hospital for surgical treatment.  IV. Past Medical History  History of chronic diseases: Denies having a history of chronic diseases such as hypertension, diabetes, and coronary heart disease.  History of infectious diseases: No.  Allergy history: No known history of drug or food allergies.  Surgical history: No history.  Family history: Denies any special genetic disease history.  Personal history: No smoking or drinking habits.  V. Physical Examination  T: 37.8℃ P: 98 beats per minute R: 20 breaths per minute BP: 110/70 mmHg  General condition: Conscious, normal mental state, no obvious signs of pain, able to cooperate with physical examination.  Skin and mucous membranes: No jaundice, no rashes.  Abdomen:  Visual examination: The abdomen is flat and there are no scars.  Palpation: The entire abdomen is soft. There is a significant tenderness at the McBurney's point in the right lower abdomen, accompanied by rebound tenderness and mild muscle tension. When there is pain in the left lower abdomen, pain also occurs in the right lower abdomen (Rovsing's sign is positive).  Percussion: The liver dullness border is normal and the fluid wave is negative.  Listening: Abdominal sounds are normal, approximately 4 times per minute.  Rectal examination: Slight tenderness on the right anterior wall of the rectum.  Other system checks: No obvious abnormalities were found.  VI. Supplementary Examinations (Main Examinations after Admission)  Blood routine: WBC 14.5x10^9/L (neutrophil ratio 85%), Hb 140 g/L, Plt 280x10^9/L.  Urinalysis: Normal.  Liver and kidney functions: Normal.  Electrolyte: Normal.  C-reactive protein (CRP): 35 mg/L  Abdominal ultrasound:  A blind tubular structure with a diameter of approximately 0.9 cm can be observed in the right lower abdominal area near the appendix. The wall of the tube is thickened, and a small amount of liquid-like dark area can be seen in the inner cavity. There is no significant deformation when the ultrasound probe is pressed. The local intestinal peristalsis is weakened.  (Conclusion: Considering acute appendicitis)  VII. Diagnosis  Acute appendicitis |
| 6.Case information: Patients scheduled for transurethral resection of the prostate (TURP) |
| I. Basic Information  Name: Meng M.  Gender: Male  Age: 75 years old  Admission date: July 18, 2025  Ethnicity: Han ethnicity  Occupation: Retired official  Marital status: Married  Residence: A certain city in central China  II. Chief Complaint  Progressive difficulty in urination for 5 years, which worsened with urinary retention for 2 days.  III. Present Illness History  The patient began experiencing difficulty in urination 5 years ago, mainly manifested as a thinner urine stream, difficulty in urination, hesitation in urination, and increased nocturia (2-3 times at night). No systematic treatment was given at that time. In the past 2 years, these symptoms have progressively worsened. The urine stream became thinner, the urination time prolonged, and it was necessary to exert a lot of force to completely empty the bladder. The nocturia increased to 3-4 times, affecting sleep. 2 days ago, the patient experienced complete inability to urinate, accompanied by bladder distension and pain, and was unable to urinate on his own. He was admitted to the hospital for emergency treatment under the accompaniment of his family. An emergency catheterization was performed, and approximately 1000ml of urine was drained. After admission, relevant examinations were completed. It was considered to be benign prostatic hyperplasia with acute urinary retention. A transurethral prostatectomy was planned.  IV. Past Medical History  History of hypertension: 10 years. Taking antihypertensive drugs, blood pressure is well controlled.  Diabetes history: Denial.  History of coronary heart disease: No history.  History of prostatitis: No.  Allergy history: No known history of drug or food allergies.  Surgical history: No history.  Family history: Denies any special genetic disease history.  V. Physical Examination  T: 36.7℃ P: 70 beats/min R: 18 breaths/min BP: 135/85 mmHg  General condition: Conscious, energetic, cooperative.  Urinary and reproductive system:  Kidney area: No bulge, no percussion pain.  Bladder area: No obvious bulge, no tenderness (urination was performed upon admission).  External genitalia: Normal.  Digital Rectal Examination (DRE):  The tension of the anal sphincter is normal.  The prostate enlargement is approximately degree II (about the size of a duck egg). The central sulcus has become shallower. The texture is firm and the surface is smooth. There are no nodules and no tenderness upon palpation.  The rectal probe has no blood staining.  Other system checks: No obvious abnormalities were found.  VI. Supplementary Examinations (Main Examinations after Admission)  Blood routine: WBC 7.0 x 10^9/L, Hb 135 g/L, Plt 260 x 10^9/L.  Urinalysis: Urine protein (-), urine glucose (-), microscopic examination shows 0-2 red blood cells per high-power field, 0-2 white blood cells per high-power field.  Renal function: Creatinine 90 umol/L, Urea Nitrogen 6.5 mmol/L (slightly elevated, possibly related to urinary retention).  Electrolyte: Normal.  Tumor marker: Prostate-specific antigen (PSA) Total PSA 4.5 ng/mL (slightly above the normal upper limit, but still within the range commonly seen in benign hyperplasia).  Electrocardiogram (ECG): Sinus rhythm, normal electrocardiogram.  Chest X-ray: Both lungs are clear, and the size of the heart is normal.  Urinary system ultrasound:  Prostate: Significantly enlarged, with a volume of approximately 60 ml (normal adult males have a volume of about 20 ml), and the internal echo is uniform.  Bladder: The wall shows no significant thickening and there is no residual urine (after catheterization).  Both kidneys and ureters: No obvious hydronephrosis.  (Conclusion: Prostate Enlargement)  VII. Diagnosis  Benign prostatic hyperplasia (BPH)  Acute urinary retention |

Table A.3: The preoperative education materials for patients generated by the large language model

| Prompt:now assume you are a medical professional, and your task is to provide comprehensive preoperative education for a patient about to undergo surgery. Please generate a complete preoperative education material based on the patient information and surgical details provided by me. Include all aspects of preoperative education and avoid using complex medical terms to ensure the readability of the material. |
| --- |
| GPT-4o |
| 1.Case information: Patients scheduled for coronary artery bypass grafting (CABG) |
| Answer:  **Preoperative Education Material for Coronary Artery Bypass Grafting (CABG)**  **Introduction**  Hello Mr. Wang,  You are scheduled to undergo coronary artery bypass grafting (CABG) to improve the blood flow to your heart. This surgery is important because it will help relieve your chest pain and reduce the risk of heart attack. Here’s what you need to know before your surgery.  **Understanding Your Condition**  You have coronary artery disease, which means the blood vessels that supply your heart are narrowed or blocked. This is causing your chest pain and shortness of breath. CABG will create new pathways for blood to reach your heart, bypassing the blocked areas.  **Preparing for Surgery**   1. **Before Surgery:**    - **Medications:** Continue taking your prescribed medications unless your doctor advises otherwise. You may need to stop certain medications like blood thinners a few days before surgery.    - **Diet:** Eat a balanced diet and avoid heavy meals the night before surgery. You will be asked not to eat or drink anything after midnight before your surgery day.    - **Smoking and Alcohol:** You have quit smoking, which is great. Avoid alcohol in the days leading up to your surgery.    - **Exercise:** Gentle exercise is good, but avoid strenuous activities that trigger chest pain. 2. **Pre-Surgery Tests:**    - You will undergo several tests such as blood tests, an ECG, and possibly a chest X-ray to ensure you are ready for surgery.   **Day of Surgery**   1. **Arrival:**    - Arrive at the hospital on time as instructed. You will be checked in and prepared for surgery. 2. **Preparation:**    - You will change into a hospital gown and your vital signs will be monitored.    - An intravenous (IV) line will be started to give you fluids and medications. 3. **Anesthesia:**    - You will receive anesthesia to ensure you are asleep and pain-free during the surgery.   **What Happens During Surgery**   - The surgeon will take a healthy blood vessel from another part of your body (like your leg or chest) and use it to bypass the blocked arteries in your heart. - The surgery usually takes 3 to 6 hours, and you will be closely monitored throughout.   **After Surgery**   1. **Recovery Room:**    - You will wake up in the recovery room where nurses will monitor you closely.    - You may have tubes to help with breathing and drainage, which is normal. 2. **Hospital Stay:**    - Expect to stay in the hospital for about 5 to 7 days. You will gradually start moving around and eating as you recover. 3. **Pain Management:**    - You will receive medications to manage any pain or discomfort. 4. **Activity:**    - You will be encouraged to walk and do breathing exercises to help your recovery.   **Going Home**   1. **Follow-Up Care:**    - You will have follow-up appointments to check your progress.    - Continue taking your medications as prescribed. 2. **Lifestyle Changes:**    - Adopt a heart-healthy lifestyle: balanced diet, regular exercise, and stress management.    - Keep your blood pressure, blood sugar, and cholesterol levels under control. 3. **Warning Signs:**    - Contact your doctor if you experience fever, increased pain, or any signs of infection.   **Conclusion**  Your surgery is an important step towards improving your heart health. Our team is here to support you every step of the way. Please feel free to ask any questions or express any concerns you may have.  Wishing you a smooth surgery and a speedy recovery!  Sincerely,  [Your Medical Team] |
| 2.Case information: Patients scheduled for total knee arthroplasty (TKA) |
| Answer:  **Preoperative Education Material for Li M.**  **Introduction:** Hello Li! As you prepare for your upcoming knee surgery, we want to ensure you have all the information you need to feel comfortable and confident. This guide will walk you through what to expect before, during, and after your total knee arthroplasty (TKA).  **Understanding Your Condition:** You have been experiencing pain and difficulty moving your right knee due to severe osteoarthritis. This condition has led to an inward deformity (O-shaped leg) and has affected your daily activities. The surgery aims to relieve pain and improve your knee function.  **About the Surgery:** Total knee arthroplasty involves replacing the damaged parts of your knee with artificial components. This will help restore movement and reduce pain. The surgery is planned for your right knee.  **Before Surgery:**   1. **Preoperative Tests:**    - You have already completed several tests, including blood work, X-rays, and an ECG. These help us ensure you are ready for surgery. 2. **Medications:**    - Continue taking your blood pressure medication as prescribed.    - Stop any medications that might affect blood clotting, as advised by your doctor.    - Inform your medical team about your penicillin allergy. 3. **Diet and Lifestyle:**    - Eat a balanced diet and stay hydrated.    - Avoid alcohol and smoking, as they can affect healing. 4. **Preparation:**    - Arrange for someone to help you at home after surgery.    - Prepare your home for easy movement, such as removing tripping hazards.   **Day of Surgery:**   1. **Arrival:**    - Arrive at the hospital on time. You will be checked in and prepared for surgery. 2. **Procedure:**    - You will receive anesthesia to ensure you are comfortable and pain-free during the surgery.    - The surgery typically lasts a few hours. 3. **Recovery Room:**    - After surgery, you will be taken to a recovery room where your vital signs will be monitored.   **After Surgery:**   1. **Hospital Stay:**    - Expect to stay in the hospital for a few days. You will receive pain management and begin physical therapy. 2. **Pain Management:**    - You will be given medications to manage pain. Inform your nurse if you experience discomfort. 3. **Physical Therapy:**    - Physical therapy is crucial for recovery. You will start exercises to improve knee movement and strength. 4. **Home Care:**    - Follow instructions for wound care and medication.    - Continue physical therapy exercises at home as directed. 5. **Follow-Up:**    - Attend all follow-up appointments to monitor your progress.   **Potential Risks:** While complications are rare, they can include infection, blood clots, or issues with the artificial knee. Your medical team will take steps to minimize these risks.  **Conclusion:** Your total knee arthroplasty is a significant step towards improving your quality of life. We are here to support you throughout the process. If you have any questions or concerns, please reach out to your medical team.  **Contact Information:**   - Hospital Phone Number: [Insert Number] - Your Doctor’s Name: [Insert Name] - Emergency Contact: [Insert Number]   We wish you a smooth surgery and a speedy recovery! |
| 3.Case information: Patients scheduled for laparoscopic cholecystectomy (LC) |
| Answer:  **Preoperative Education Material for Zhang M.**  **Introduction:**  Hello Zhang M.,  You are scheduled for a laparoscopic cholecystectomy, which is a surgery to remove your gallbladder. This procedure is recommended because you have gallbladder stones and acute cholecystitis, which have been causing you pain and discomfort. Our goal is to help you understand the surgery, what to expect, and how to prepare for it.  **Understanding Your Condition:**   1. **Gallbladder Stones:** These are small, hard deposits that form in your gallbladder. They can cause pain, especially after eating fatty foods, and lead to nausea and vomiting. 2. **Acute Cholecystitis:** This is an inflammation of the gallbladder, often caused by gallstones. It can result in severe pain and requires prompt treatment.   **About the Surgery:**   - **Laparoscopic Cholecystectomy:** This is a minimally invasive surgery where small cuts are made in your abdomen. A camera and special tools are used to remove your gallbladder. This method usually means less pain and a quicker recovery compared to traditional surgery.   **Preoperative Instructions:**   1. **Before Surgery:**    - **Fasting:** Do not eat or drink anything after midnight before your surgery day. This includes water, gum, and candy.    - **Medications:** Follow your doctor’s instructions regarding any medications you should take or avoid before surgery.    - **Arrival Time:** You will be informed about what time to arrive at the hospital. Make sure to be on time. 2. **Day of Surgery:**    - **Clothing:** Wear comfortable, loose-fitting clothes. You will change into a hospital gown for the procedure.    - **Personal Items:** Leave valuables at home. Bring only essentials like your ID and insurance card.    - **Support:** Arrange for someone to drive you home after the surgery, as you will not be able to drive yourself.   **What to Expect:**   1. **During Surgery:**    - You will receive anesthesia, so you will be asleep and pain-free during the procedure.    - The surgery typically lasts about 1 to 2 hours. 2. **After Surgery:**    - You will be taken to a recovery area where nurses will monitor you as you wake up from anesthesia.    - You may feel groggy and have some discomfort in your abdomen, which is normal. 3. **Recovery:**    - Most patients go home the same day, but some may need to stay overnight.    - You will receive instructions on managing pain, caring for your incisions, and when you can resume normal activities.   **Postoperative Care:**   1. **Pain Management:** You may be given medication to help manage any pain or discomfort. Follow the dosage instructions carefully. 2. **Activity:** Rest is important. Gradually increase your activity level as you feel better. Avoid heavy lifting or strenuous activities for a few weeks. 3. **Diet:** Start with light meals and gradually return to your regular diet. Avoid fatty foods initially to prevent discomfort. 4. **Follow-Up:** Attend any scheduled follow-up appointments to ensure proper healing and address any concerns.   **Questions and Concerns:**  If you have any questions or concerns before or after the surgery, please do not hesitate to contact your healthcare team. We are here to support you every step of the way.  **Conclusion:**  We are committed to providing you with the best care possible. Understanding the process and what to expect can help ease your mind and prepare you for a successful surgery and recovery. We look forward to helping you feel better soon.  Best wishes for your surgery and recovery,  [Your Healthcare Team] |
| 4.Case information: Patients scheduled for transcatheter arterial chemoembolization (TACE) |
| Answer:  **Preoperative Education Material for Ma M.**  **Introduction**  Hello Ma M.,  You are scheduled to undergo a procedure called Transcatheter Arterial Chemoembolization (TACE) to treat your liver cancer. This guide will help you understand what to expect before, during, and after the procedure. Our goal is to ensure you feel informed and comfortable with the upcoming treatment.  **Understanding Your Condition**  You have been diagnosed with hepatocellular carcinoma, a type of liver cancer, and liver cirrhosis related to hepatitis B. TACE is a treatment that targets the cancer directly in the liver, helping to shrink the tumor and reduce symptoms like abdominal pain and fatigue.  **What is TACE?**  TACE is a minimally invasive procedure that delivers chemotherapy directly to the liver tumor through the blood vessels. It helps block the blood supply to the tumor, which can slow its growth or shrink it.  **Before the Procedure**   - **Preoperative Tests:** You will undergo several tests to ensure you are ready for the procedure. These include blood tests, imaging studies, and possibly other assessments. - **Medications:** Your doctor will review your current medications. You may need to stop certain medications before the procedure. Follow your doctor’s instructions carefully. - **Diet:** You may be asked to avoid eating or drinking for a certain period before the procedure. This is important for your safety during anesthesia. - **Smoking and Alcohol:** Continue to avoid smoking and alcohol, as they can affect your liver and overall health.   **Day of the Procedure**   - **Arrival:** You will be asked to arrive at the hospital on the morning of the procedure. Make sure to bring any necessary documents and personal items. - **Preparation:** You will change into a hospital gown and be taken to the procedure room. A nurse will check your vital signs and start an IV line for fluids and medications. - **Anesthesia:** You will receive local anesthesia to numb the area where the catheter will be inserted. You may also receive medication to help you relax.   **During the Procedure**   - **Catheter Insertion:** A small tube (catheter) will be inserted into an artery in your groin and guided to the liver. You may feel some pressure but should not feel pain. - **Chemoembolization:** The chemotherapy and blocking agents are delivered through the catheter directly to the tumor. This part of the procedure usually takes 1-2 hours.   **After the Procedure**   - **Recovery:** You will be taken to a recovery area where nurses will monitor you closely. You may feel tired or have mild discomfort, which is normal. - **Hospital Stay:** You may need to stay in the hospital for a day or two for observation and recovery. - **Pain Management:** You will receive medication to manage any pain or discomfort. Let your healthcare team know if you experience any severe pain or other symptoms.   **At Home**   - **Rest:** Take it easy for a few days. Avoid strenuous activities until your doctor gives you the go-ahead. - **Follow-Up:** Attend all scheduled follow-up appointments. These are important to monitor your recovery and the effectiveness of the treatment. - **Diet and Lifestyle:** Continue to eat a balanced diet and avoid alcohol. Follow any specific dietary recommendations provided by your healthcare team.   **When to Contact Your Doctor**  Reach out to your doctor if you experience:   - Severe pain or swelling at the catheter site - Fever or chills - Yellowing of the skin or eyes - Difficulty breathing or chest pain   **Support and Resources**  Dealing with liver cancer can be challenging, but you are not alone. Consider joining a support group or speaking with a counselor to help manage stress and emotions.  **Conclusion**  We are committed to providing you with the best care possible. If you have any questions or concerns, please do not hesitate to ask your healthcare team. We are here to support you every step of the way.  Wishing you a smooth procedure and a speedy recovery.  Sincerely,  Your Healthcare Team |
| 5.Case information: Patients scheduled for appendectomy |
| Answer:  **Preoperative Education Material for Chen M.**  **Introduction:** Hello Chen, welcome to our hospital. We understand that you are scheduled for an appendectomy, which is a surgery to remove your appendix. This guide will help you understand what to expect before, during, and after your surgery. Our goal is to make sure you feel informed and comfortable about the process.  **Understanding Appendicitis:** The appendix is a small tube-like organ attached to the large intestine. Sometimes, it can become inflamed and infected, which is known as appendicitis. This can cause pain, especially in the lower right side of your abdomen, as you have experienced. Removing the appendix is a common and effective treatment.  **Before Surgery:**   1. **Preparation:**    - **Fasting:** You will need to stop eating and drinking for a certain period before the surgery. This is usually about 6-8 hours prior. Your nurse will inform you of the exact time.    - **Medications:** Inform your doctor about any medications you are taking. You may need to stop some of them temporarily.    - **Consent:** You will be asked to sign a consent form, which means you agree to the surgery after understanding the risks and benefits. 2. **Tests and Monitoring:**    - You have already undergone several tests, including blood tests and an ultrasound, which confirmed acute appendicitis. These tests help ensure you are ready for surgery.   **Day of Surgery:**   1. **Arrival:**    - You will be taken to the preoperative area where nurses will check your vital signs (temperature, pulse, and blood pressure) and ensure everything is ready. 2. **Anesthesia:**    - You will receive anesthesia, which means you will be asleep and pain-free during the surgery. The anesthesiologist will discuss this with you beforehand. 3. **Procedure:**    - The surgery usually takes about 1-2 hours. The surgeon will make a small cut in your abdomen to remove the appendix. Sometimes, they use a special camera and tools to do this through tiny cuts, which is called laparoscopic surgery.   **After Surgery:**   1. **Recovery:**    - You will wake up in the recovery room where nurses will monitor you closely. You might feel sleepy or groggy initially.    - Pain management is important. You will receive medication to help manage any discomfort. 2. **Hospital Stay:**    - Most patients stay in the hospital for a day or two after an appendectomy. You will be encouraged to move around gently to help your recovery. 3. **Diet:**    - You will start with liquids and gradually move to solid foods as your body adjusts. 4. **Activity:**    - Avoid strenuous activities for a few weeks. Walking and light activities are encouraged to help circulation and healing.   **Signs to Watch For:**   - Fever, increased pain, redness, or swelling around the incision site. - Difficulty breathing or persistent nausea.   **Follow-Up:**   - You will have a follow-up appointment to check your recovery progress. Your doctor will discuss any further care or restrictions.   **Conclusion:** We are here to support you throughout this process. If you have any questions or concerns, please feel free to ask any member of your healthcare team. Your health and comfort are our top priorities.  **Contact Information:**   - Hospital Phone Number: [Insert Number] - Emergency Contact: [Insert Number]   We wish you a smooth surgery and a quick recovery! |
| 6.Case information: Patients scheduled for transurethral resection of the prostate (TURP) |
| Answer:  **Preoperative Education Material for Meng M.**  **Introduction**  Hello Meng, and welcome to our hospital. We understand that you are scheduled for a procedure called transurethral resection of the prostate (TURP) to help with your urinary difficulties. This guide will provide you with all the information you need to prepare for your surgery and help you understand what to expect before, during, and after the procedure.  **Understanding Your Condition**  You have been diagnosed with benign prostatic hyperplasia (BPH), which means your prostate gland has grown larger than normal. This enlargement can block the flow of urine from the bladder, causing symptoms like a weak urine stream, difficulty starting urination, and frequent nighttime urination. Recently, you experienced acute urinary retention, meaning you couldn’t urinate at all, which required emergency treatment.  **What is TURP?**  TURP is a common surgical procedure used to treat BPH. During the surgery, the doctor will remove parts of your prostate that are blocking urine flow. This is done through the urethra, the tube that carries urine from the bladder out of the body, so there are no external cuts or stitches.  **Preparing for Surgery**   1. **Medications:** Continue taking your blood pressure medication as directed. Inform your doctor about all medications and supplements you are taking, as some may need to be stopped before surgery. 2. **Diet:** Follow any dietary instructions given by your healthcare team. You may be asked to avoid eating or drinking after midnight the night before your surgery. 3. **Preoperative Tests:** You have already completed necessary tests, including blood work and imaging studies. These help ensure you are ready for surgery. 4. **Arrival:** Plan to arrive at the hospital on July 18, 2025, as instructed. Bring any personal items you might need, such as comfortable clothing and toiletries.   **What to Expect on the Day of Surgery**   - **Check-in:** You will check in at the hospital and be taken to a preoperative area. - **Preparation:** Nurses will help you change into a hospital gown and may start an IV to give you fluids and medications. - **Anesthesia:** You will receive anesthesia to ensure you are comfortable and pain-free during the procedure. You will be asleep and won’t feel anything.   **After the Surgery**   - **Recovery Room:** After the procedure, you will be taken to a recovery room where nurses will monitor you as you wake up from anesthesia. - **Catheter:** You will have a catheter in place to help drain urine from your bladder. This is temporary and will be removed once you are able to urinate on your own. - **Pain Management:** You may experience some discomfort or mild pain, which can be managed with medication. - **Hospital Stay:** Expect to stay in the hospital for a short period to ensure you are recovering well.   **Going Home**   - **Instructions:** You will receive detailed instructions on how to care for yourself at home, including managing any discomfort and recognizing signs of complications. - **Follow-up:** A follow-up appointment will be scheduled to check your progress and remove the catheter if it hasn’t been done already.   **Tips for Recovery**   - **Rest:** Allow yourself time to rest and recover. Avoid heavy lifting or strenuous activities for a few weeks. - **Hydration:** Drink plenty of water to help flush your urinary system. - **Diet:** Eat a balanced diet to support healing.   **When to Contact Your Doctor**  Reach out to your healthcare team if you experience:   - Fever or chills - Severe pain not relieved by medication - Difficulty urinating after the catheter is removed - Blood in your urine that doesn’t improve   **Conclusion**  We are here to support you every step of the way. If you have any questions or concerns, please do not hesitate to ask your healthcare team. We wish you a smooth surgery and a speedy recovery. |
| Gemini-2.5-Pro |
| 1.Case information: Patients scheduled for coronary artery bypass grafting (CABG) |
| Answer:  Of course. Based on the detailed information provided for Mr. Wang, here is a comprehensive preoperative education guide tailored to his specific condition and upcoming Coronary Artery Bypass Grafting (CABG) surgery. The language is kept simple and clear to ensure he and his family can easily understand the important information.  **Your Guide to Preparing for Heart Bypass Surgery**  **Patient:** Mr. Wang M. **Procedure:** Coronary Artery Bypass Grafting (Heart Bypass Surgery) **Admission Date:** July 10th, 2025  **Location:** [Hospital Name and Address to be inserted here] **Your Scheduled Arrival Time:** [Time to be inserted here]  **A Personal Message for You, Mr. Wang**  Hello Mr. Wang,  This guide has been prepared especially for you and your family. We know that preparing for heart surgery can feel overwhelming, and our goal is to give you clear information to help you feel confident and ready. Your health and safety are our highest priorities.  The purpose of your upcoming surgery is to restore healthy blood flow to your heart muscle. Think of it as creating new pathways, or “bypasses,” around the blockages in your heart’s arteries. This will help relieve your chest pain, reduce shortness of breath, and allow you to be more active.  Please read this guide carefully with your wife and family. Write down any questions you have, and we will be happy to answer them.  **Section 1: Understanding Your Health and Its Importance for Surgery**  Your medical history shows you have managed several conditions for many years. Getting these under the best possible control before surgery is the most important thing you can do for a successful recovery.  **1. Managing Your Blood Sugar (Diabetes)**   - **Why it’s important:** Stable blood sugar is critical for healing. High blood sugar can slow down how your body repairs itself and increases the risk of infection after surgery, especially at your chest incision. - **What you need to do:**   - Follow the diet plan discussed with your doctors very carefully.   - Take your diabetes medication exactly as prescribed.   - Monitor your blood sugar levels as instructed. Please keep a log of your readings to share with us.   - We will have specific instructions for your diabetes medication for the morning of surgery.   **2. Controlling Your Blood Pressure (Hypertension)**   - **Why it’s important:** Keeping your blood pressure stable before, during, and after surgery reduces stress on your heart and blood vessels. - **What you need to do:**   - Continue taking your blood pressure medication as prescribed. We will tell you exactly which ones to take on the morning of surgery.   - Try to incorporate relaxing activities into your day, such as listening to music or spending quiet time with your family.   **3. Preparing Your Lungs (Your history as a smoker)**   - **A special note:** Quitting smoking last year was one of the best things you could have done for your health and for this surgery. Congratulations! This has already made your surgery safer. - **What you need to do now:** To prevent lung problems like pneumonia after surgery, we need to practice “lung exercises” now.   - **Deep Breathing and Coughing:** Several times a day, take 5-10 deep, slow breaths. On the last breath, hold it for a few seconds, then give a strong, firm cough. This helps keep your lungs clear.   - **Incentive Spirometer:** We will give you a simple plastic breathing device. Your nurse will show you how to use it. The goal is to inhale slowly and deeply to raise the piston inside. Using this 10 times every hour you are awake will help keep your lungs strong.   **Section 2: The Days Leading Up to Your Surgery**  **Medication Plan – VERY IMPORTANT** Your nurse will provide you with a detailed list of which medications to **STOP** and which to **CONTINUE**.   - **Blood Thinners:** Patients are often asked to stop taking blood-thinning medications (like aspirin, clopidogrel, etc.) 5-7 days before surgery to reduce bleeding. - **Blood Pressure and Heart Medications:** You will likely continue some of these. - **Diabetes Medications:** We will give you very specific instructions for your oral medications or insulin. - **It is critical that you follow these instructions exactly.** Please bring a complete list of all your medications, including doses, with you to the hospital.   **Nutrition and Hydration**   - Eat balanced, healthy meals. Focus on protein (fish, chicken, eggs, beans) to help build strength for recovery. - If you have diabetes, stick to your recommended diet. - Drink plenty of water to stay well-hydrated.   **Planning for Your Hospital Stay**   - **What to Pack:**   - Your national ID card and any insurance information.   - A list of your medications and allergies.   - Personal toiletries (toothbrush, comb, etc.).   - Comfortable, loose-fitting clothes for when you are able to walk around and for going home. A button-up or zip-up shirt is often easiest.   - Eyeglasses, hearing aids, or dentures with their cases. - **What to Leave at Home:**   - Jewelry, watches, and any other valuables.   - Large amounts of cash.   **Section 3: The Day Before and Morning of Your Surgery**  **Skin Preparation to Prevent Infection** Your skin has normal bacteria on it. To prevent this bacteria from causing an infection in your incision, you must wash with a special antiseptic soap.   - **The Night Before:** Shower or bathe using the antiseptic soap we provide. Pay special attention to your entire chest, from your neck to your belly button. - **The Morning of Surgery:** Take a second shower or bath with the same soap. - **DO NOT** apply any lotions, powders, or deodorants after your final shower. Do not shave your chest; we will do this for you in the hospital if needed.   **Eating and Drinking – A Critical Safety Rule** Your stomach must be completely empty for anesthesia to be safe.   - You will be told when to have your last light meal. - **After Midnight on the night before your surgery, you must not eat or drink anything.** This includes water, tea, gum, or candy. - If you have been told to take certain medications on the morning of surgery, you may take them with **only a small sip of water.** - **If you do not follow this rule, your surgery will be cancelled for your safety.**   **Section 4: A Look at Your Hospital Stay After Surgery**  It is helpful to know what to expect. This is a major operation, and your recovery will happen in stages.  **1. The Intensive Care Unit (ICU)**   - Immediately after your surgery, you will be taken to the ICU. This is normal for all heart surgery patients. Here, you will have one-on-one nursing care. - **Monitors and Tubes:** You will wake up connected to several machines and tubes. This can be startling, but they are all there to help you.   - You will have a **breathing tube** in your mouth connected to a machine that helps you breathe. You will not be able to talk with it in. It is usually removed within a few hours of surgery, once you are awake enough to breathe well on your own.   - You will have an IV line for fluids and medication, a tube to monitor your blood pressure, and stickers on your chest to monitor your heart.   - You will have one or more **chest tubes** coming from below your incision. These drain normal fluid from around your heart and are usually removed in 1-2 days. - Your family will be able to visit you in the ICU once you are settled.   **2. Moving to the Regular Cardiac Ward**   - After 1-2 days, when you are stable, you will move to the regular hospital floor. The nurses here are also experts in caring for heart surgery patients.   **3. Your Recovery in the Hospital**   - **Pain Management:** You will have pain, especially at your incision. It is important to tell your nurse about your pain so we can give you medication to keep you comfortable. Being comfortable will help you breathe deeply and start moving. - **Getting Up and Walking:** This is one of the most important parts of your recovery. We will help you sit in a chair the day after surgery and start walking soon after. Walking helps prevent blood clots, pneumonia, and builds your strength. - **Cardiac Rehabilitation:** A team will begin working with you in the hospital to start you on the path to a healthy, active life. This will continue after you go home.   **Final Checklist for You and Your Family**  [ ] We have discussed our questions with the doctors and nurses. [ ] We understand the plan for blood sugar and blood pressure control. [ ] We have the instructions for which medications to stop and continue. [ ] We understand the rules for eating and drinking before surgery. [ ] We have planned for the hospital stay and packed the necessary items.  **Our Commitment to You** Mr. Wang, you have a strong heart, and this surgery is the next step toward a healthier future. Your entire care team is here to support you and your family through every phase of this journey. Do not hesitate to ask us anything.  We look forward to taking care of you.  Sincerely, **Your Surgical and Cardiology Care Team** |
| 2.Case information: Patients scheduled for total knee arthroplasty (TKA) |
| Answer: Of course. Based on the detailed information provided for Ms. Li M., here is a comprehensive and patient-friendly preoperative education guide for her upcoming Total Knee Replacement surgery.  **A Guide to Preparing for Your Right Knee Replacement**  **Prepared for:** Ms. Li M. **Date of Guide:** July 15, 2025  Hello Ms. Li,  This guide has been prepared by your healthcare team to help you understand and prepare for your upcoming total knee replacement surgery. We know you have been living with significant knee pain for a long time, and we are committed to helping you on this journey to better mobility and less pain.  Knowing what to expect can help you feel more comfortable and confident. Please read this information carefully and write down any questions you have. We are here to support you.  **1. Your Surgical Procedure at a Glance**   - **Your Procedure:** Total Knee Replacement of your **Right Knee**. - **Purpose of Your Surgery:** To replace the worn-out, painful surfaces of your knee joint with new, artificial parts. This is designed to relieve the severe pain you’ve been experiencing, correct the “O-shaped” bowing of your leg, and help you walk more easily and get back to your daily life. - **Your Surgeon:** [Surgeon's Full Name] - **Date of Surgery:** [Date of Surgery] - **Scheduled Arrival Time:** [Time, e.g., 6:00 AM] - **Please note, this is NOT your surgery time.** This is the time you need to arrive at the hospital for check-in and preparation. - **Location:** [Hospital/Surgical Center Name] - **Address:** [Full Address of Facility] - **Check-in Location:** [Specific location, e.g., "Main Admitting Desk, 1st Floor"]   **2. Preparing in the Weeks and Days Before Surgery**  Proper preparation is key to your safety and a successful outcome.  **Medications & Important Health Information**  It is **critically important** that we have a complete picture of your health.   - **Your Penicillin Allergy:** We have on record that you are **allergic to penicillin**. We will make sure you do not receive penicillin or similar antibiotics. Please remind the nurses and doctors you see on the day of surgery about this allergy. - **Blood Pressure Medicine:** **Continue taking your blood pressure medication** exactly as prescribed. On the morning of your surgery, you should take it with a small sip of water. Keeping your blood pressure controlled is very important for a safe surgery. - **Bone Health:** Continue taking your calcium and Vitamin D tablets as you normally do. This is important for your overall bone strength. - **Medications to STOP:** Your doctor will give you a specific list of medications to stop taking before surgery, usually 5-7 days prior. These often include:   - Pain relievers like Ibuprofen (Advil, Motrin) and Naproxen (Aleve).   - Blood thinners (your doctor will give you specific instructions if you take any).   - Some vitamins and herbal supplements like Vitamin E and Fish Oil. **ACTION REQUIRED:** Please confirm with our office which medications you should stop and when.   **Diet and Lifestyle**   - **Nutrition:** In the weeks before surgery, focus on eating a balanced diet with plenty of protein (found in meat, fish, eggs, and beans). Good nutrition helps your body prepare for surgery and heal afterward. - **Alcohol:** Stop drinking all alcoholic beverages at least 48 hours before your surgery. - **Smoking/Vaping:** If you smoke, quitting now is the single best thing you can do for your recovery. Smoking can increase the risk of infection and breathing problems and slow down bone and skin healing.   **Planning for Your Recovery at Home**   - **Transportation:** You will not be able to drive after your surgery. You **must** arrange for a responsible adult (like your husband or another family member) to drive you home. - **Support at Home:** For the first 1-2 weeks, you will need help with cooking, cleaning, and shopping. Please arrange for this support ahead of time. - **Prepare Your Home:**   - Remove tripping hazards like loose rugs, cords, and clutter.   - Consider setting up a “recovery station” on the main floor to avoid stairs.   - Place a firm chair with armrests in your main living area to make it easier to stand up.   - Place frequently used items within easy reach.   **3. The Day Before and Morning of Your Surgery**  **The “Nothing by Mouth” Rule - For Your Safety!**  This is the most important rule. If you do not follow it, your surgery will be cancelled to ensure your safety.   - **NO solid food, milk, or juice with pulp after Midnight** the night before your surgery. - **You MAY drink clear liquids up to [Time, e.g., 4:00 AM] on the day of surgery.** Clear liquids are liquids you can see through. Examples include:   - Water   - Black coffee or plain tea (NO milk or creamer)   - Apple juice - **After [Time, e.g., 4:00 AM], DO NOT EAT OR DRINK ANYTHING.** This includes water, gum, and mints. The only exception is the small sip of water needed to take your blood pressure pill.   **Getting Ready**   - **Showering:** Please shower the night before or the morning of surgery with an antibacterial soap. Wash your whole body, paying special attention to your right leg and knee. Do not apply any lotions, deodorants, or perfumes after your shower. - **Do NOT shave your right leg** for at least two days before surgery. Shaving can cause tiny nicks in the skin that can increase the risk of infection. - **What to Wear:** Wear loose, comfortable clothing like sweatpants and a button-up shirt.   **4. At the Hospital**   1. **Preparation Area:** You will change into a hospital gown. A nurse will check your blood pressure and pulse. An IV (a small, soft tube) will be placed in your arm to give you fluids and medications. 2. **Meeting the Team:** You will meet the anesthesiologist, who will discuss the plan to keep you comfortable and pain-free. Your surgeon will also see you to answer any last-minute questions and mark your right knee for surgery. 3. **Anesthesia:** You will be given medicine to make you fall asleep. You will be completely comfortable and unaware during the procedure. 4. **Recovery Room:** After surgery, you will wake up in the recovery room. A nurse will be with you at all times. It is normal to feel sleepy and a bit groggy.   **5. Your Recovery in the Hospital**   - **Pain Management:** We will work with you to keep your pain under control with medication. Good pain control is essential for you to be able to rest and participate in physical therapy. - **Physical Therapy:** This is a very important part of your recovery! A physical therapist will likely visit you **on the same day as your surgery** to help you stand and take a few steps. They will teach you exercises to regain your movement and strengthen the muscles around your new knee. - **Preventing Complications:**   - **Blood Clots:** To prevent blood clots in your legs, you may have special compression stockings or sleeves on your legs. You will also be given a blood-thinning medication and encouraged to do foot and ankle pump exercises. Getting up and walking is the best prevention.   - **Pneumonia:** You will be encouraged to take deep breaths and cough regularly to keep your lungs clear. - **Your Incision (Wound):** Your surgical cut will be covered with a dressing. Nurses will monitor it. Please do not touch it.   **6. Going Home and Recovering at Home**   - **Discharge Instructions:** You will receive detailed written instructions before you go home covering wound care, medications, and your physical therapy plan. - **Activity:**   - Use your walker or cane as instructed by your physical therapist.   - Avoid lifting anything heavier than a gallon of milk (about 8-10 lbs) for the first 6 weeks.   - You will not be able to drive for several weeks, until your surgeon says it is okay. - **Follow-up Appointment:** Your first follow-up visit is scheduled for:   - **Date & Time:** [Date and Time]   - **With:** [Doctor's Name]   - **At:** [Location of Clinic]   **7. When to Call Your Doctor or Seek Emergency Care**  Please call our office immediately if you experience any of the following:   - A fever higher than 101.5°F (38.6°C). - Pain in your right knee that gets worse and is not controlled by your medication. - Increased redness, swelling, or warmth around the incision. - Any pus or foul-smelling drainage from your incision.   **Go to the nearest Emergency Room or call 911 for:**   - **Sudden chest pain.** - **Sudden difficulty breathing.** - **Pain, tenderness, or significant swelling in your calf (lower leg). This could be a sign of a blood clot.**   **8. Important Contact Information**   - **Surgeon’s Office:** [Phone Number]   - **Hours:** [Office Hours] - **After-Hours/On-Call Number:** [Phone Number]   Ms. Li, you have made an important decision to improve your quality of life. Our entire team is here to ensure you have a safe and successful surgery and recovery. We look forward to seeing you walk with more comfort and ease.  Sincerely,  **Your Care Team at [Clinic/Hospital Name]** |
| 3.Case information: Patients scheduled for laparoscopic cholecystectomy (LC) |
| Answer:  Of course. Based on the detailed information you have provided for Ms. Zhang, here is a comprehensive preoperative education guide tailored specifically for her. The material is written in simple, clear language to ensure she feels informed and prepared for her laparoscopic cholecystectomy.  **Your Guide to Preparing for Your Gallbladder Surgery**  **Patient:** Ms. Zhang M. **Procedure:** Laparoscopic Cholecystectomy (Gallbladder Removal Surgery) **Scheduled Date of Surgery:** July 15, 2025 *(Note: This date is an estimate, please confirm with the scheduling office)* **Surgeon:** [Surgeon’s Name] **Location:** [Name of Hospital/Surgical Center], [Department/Ward Name]  **A Personal Message for You, Ms. Zhang,**  Hello,  We have prepared this guide to help you understand and prepare for your upcoming gallbladder surgery. We know that this is your first time having surgery, and it’s completely normal to have questions or feel a little anxious. Our entire team is here to support you and provide the best possible care.  The reason you have been feeling that severe pain in your upper abdomen, along with nausea, is due to gallstones causing a painful inflammation of your gallbladder. This is called “acute cholecystitis.” The good news is that your tests show you are otherwise in good health, and this surgery is expected to resolve your symptoms completely.  Please read this guide carefully. It will walk you through everything from now until after your surgery.  **Section 1: Understanding Your Surgery**  The surgery you will have is called a **Laparoscopic Cholecystectomy**.   - **What does this mean in simple terms?** It means we will remove your gallbladder. - **How is it done?** Instead of one large cut, the surgeon will make a few very small incisions (cuts) in your abdomen. A tiny camera and special tools are passed through these small openings to remove the gallbladder. - **What are the benefits of this method?** This “keyhole” surgery usually means less pain after the operation, smaller scars, and a much faster recovery time compared to traditional open surgery. - **Why is this surgery necessary?** Removing the gallbladder will stop the painful attacks and prevent future problems that gallstones can cause. You can live a perfectly normal, healthy life without your gallbladder.   **Section 2: Preparing in the Days Before Surgery**  Your careful preparation is a key part of a smooth and successful surgery.  **Medical Preparations:**   - **Your Tests:** The blood tests, EKG (heart check), and X-ray you have already completed show that you are well-prepared for the procedure. The slightly elevated white blood cell count is expected, as it simply shows your body is fighting the inflammation in your gallbladder. - **Medications:** You have no history of taking blood thinners, which is excellent. However, as a general rule, please **avoid taking any over-the-counter pain relievers like Aspirin, Ibuprofen (Advil), or Naproxen (Aleve)** in the days leading up to surgery, as they can increase bleeding risk. Also, please stop any herbal supplements if you are taking them.   **Lifestyle Preparations:**   - **Healthy Eating:** In the days before your surgery, try to eat light, low-fat meals. This will be gentler on your gallbladder and help you feel more comfortable. Focus on fruits, vegetables, and lean proteins. - **Arrange Your Ride and Support:** You will not be able to drive yourself home after surgery. Your husband or another responsible adult must be available to take you home. It is also very important to have someone stay with you for the first 24 hours after you return home.   **Home Preparations:**   - **Make Your Home Recovery-Friendly:**   - Place things you use often (like your phone charger, remote control, and books) where you can easily reach them without stretching.   - Clear walkways of any loose rugs or cords to prevent tripping.   - Consider preparing a few simple meals and putting them in the refrigerator or freezer.   **Section 3: The Day Before Your Surgery (July 14, 2025)**  **This is a very important day for preparation!**  **Eating and Drinking Rules - CRITICAL:**   - **You must not eat or drink anything after midnight (12:00 AM) on the night before your surgery.** This rule is for your safety. An empty stomach is necessary to prevent any food or liquid from entering your lungs while you are under anesthesia. - This rule includes **all food, water, coffee, tea, candy, and gum.**   **Bathing:**   - Please take a shower or bath the evening before your surgery. Use a regular, gentle soap. A clean body helps reduce the risk of infection. - Do not apply any lotions, creams, deodorants, or perfumes after your shower.   **What to Pack for the Hospital:**   - Your national ID card and any hospital registration cards. - Comfortable, loose-fitting clothes and flat, slip-on shoes to wear home. - Personal items like your toothbrush and eyeglasses case. - **Please leave all valuables at home,** including jewelry, watches, and large amounts of cash.   **Section 4: The Day of Your Surgery (July 15, 2025)**   - **Arrival:** You will already be in the hospital. The nursing staff will inform you of the plan for the morning. - **Getting Ready:** You will be asked to change into a hospital gown. A nurse will review your information and check your blood pressure, pulse, and temperature. - **Meeting Your Team:** Before you go to the operating room, you will meet the surgeon and the anesthesiologist (the doctor in charge of keeping you comfortable and asleep during surgery). They will answer any final questions you have. - **IV Line:** A nurse will place a small, soft plastic tube called an IV into a vein in your arm or hand. This is how you will receive fluids and medication during the surgery. It may feel like a small pinch.   **Section 5: After Your Surgery**   - **Waking Up:** You will wake up in a recovery area called the PACU (Post-Anesthesia Care Unit). A nurse will be right there with you. It is common to feel sleepy, a bit groggy, and maybe a little cold. - **Managing Pain:** We will work to keep you comfortable. Please tell your nurse if you are feeling pain. We will ask you to rate your pain on a scale of 0 (no pain) to 10 (the worst pain) so we can give you the right medicine. - **A Note on Shoulder Pain:** It is very common to feel some pain in your right shoulder after this type of surgery. This is caused by the gas used to inflate your abdomen during the procedure and is normal. It will go away on its own in a day or two. Walking around can help. - **Going Back to Your Room:** Once you are fully awake and stable, you will be taken back to your hospital room. The nurses will encourage you to take deep breaths and to get up and walk (with assistance) as soon as you are able. This is very important to help your lungs recover and prevent blood clots.   **Section 6: When to Call for Help After You Go Home**  Your safety is our top priority. Please contact us or seek medical help if you experience any of the following signs.  **Go to the nearest Emergency Room immediately for:**   - Sudden chest pain or difficulty breathing. - Fainting or feeling like you will pass out. - Severe abdominal pain that gets worse and is not helped by your pain medicine.   **Call our hospital ward or your surgeon’s office for:**   - A fever higher than 38.5°C (101.5°F). - Signs that your small incisions might be infected: increasing redness, swelling, warmth, or thick, yellow/green liquid draining from them. - Vomiting that doesn’t stop and prevents you from keeping liquids down. - Pain that is not controlled by the medication you were given. - Yellowing of your skin or the whites of your eyes (jaundice).   **Our Ward/Office Phone Number:** [Insert Hospital Ward Phone Number] **After-Hours Contact:** [Insert After-Hours Phone Number]  We are here to ensure you have a safe surgery and a smooth recovery. Please do not hesitate to ask any member of our team any questions you may have. We look forward to caring for you.  Sincerely,  **Your Medical Care Team at [Name of Hospital/Surgical Center]** |
| 4.Case information: Patients scheduled for transcatheter arterial chemoembolization (TACE) |
| Answer:  Of course. Based on the detailed information provided for Mr. Ma, here is a comprehensive preoperative education guide tailored specifically for him and his upcoming TACE procedure. This guide is written in clear, simple language to ensure he and his family can fully understand the process.  **Your Guide to Preparing for Your TACE Procedure**  **A Personal Message for You, Mr. Ma**  Hello Mr. Ma,  This guide has been prepared especially for you by your medical team. We understand that preparing for a medical procedure can bring up many questions and concerns. Our goal is to walk you through every step of the process so you feel informed, comfortable, and confident in the care you are about to receive.  We see you as our partner in your health. Please read this information carefully, and feel free to write down any questions you have. We are here to answer them.  **1. Your Procedure Information**   - **Patient Name:** Ma M. - **Procedure:** **Transcatheter Arterial Chemoembolization (TACE)** - **Physician:** Dr. [Physician's Name] - **Date of Procedure:** [Date of Procedure] - **Scheduled Arrival Time:** [Time, e.g., 7:00 AM] - **Location:** Interventional Radiology Department, [Hospital Name] - **Address:** [Hospital Address] - **Check-in Location:** [Specific location, e.g., Radiology Admissions, Main Lobby]   **2. Understanding Your Procedure: What is TACE?**  You might be wondering what “TACE” actually is. It is not a traditional surgery with a large incision. Instead, it is a highly targeted way to treat the tumor in your liver.  Think of it like this:   1. **Creating a Road Map:** We will give you a special dye that lights up your blood vessels on an X-ray. This creates a detailed “road map” of the arteries that supply blood to your liver and, most importantly, to the tumor. 2. **Navigating to the Target:** A specialist doctor (an Interventional Radiologist) will make a very small puncture, usually in the large artery in your groin area (at the top of your leg). Through this tiny opening, they will guide a very thin, flexible tube (called a catheter) through your arteries, following the road map directly to the tumor in your liver. 3. **Delivering Treatment:** Once the tube is in the perfect spot, two things happen:    - A high dose of **cancer-fighting medicine (chemotherapy)** is delivered directly to the tumor.    - Tiny particles, like microscopic beads, are used to **block the blood flow** feeding the tumor.   By cutting off its food and blood supply and delivering medicine at the same time, this procedure attacks the tumor while having very little effect on the rest of your body and the healthy parts of your liver.  **3. Preparing in the Days Before Your Procedure**  Your existing health conditions, like hepatitis B and cirrhosis, are important factors we are managing. Your liver is scarred but still functioning well (Child-Pugh Grade A), which is good news for this procedure.  **Medications:**   - You do not currently take any blood thinners, which is helpful. - Please **do not** take any new over-the-counter medications, like pain relievers (Aspirin, Advil) or herbal supplements, without speaking to us first. - Bring a list of any and all medications you might take, even if it’s rare, to the hospital with you.   **Important Health Habits:**   - **Continue Not Smoking:** It is excellent that you have already quit smoking. This will greatly help your breathing and recovery. - **Nutrition and Fluids:** In the days leading up to the procedure, try to drink plenty of fluids and eat balanced meals to keep your body as strong as possible.   **Planning for Your Recovery:**   - **Arrange for a Ride Home:** You will be given sedation (medicine to make you sleepy and relaxed) and will **not** be able to drive yourself home. Please arrange for a family member or trusted friend to drive you. - **Arrange for Help at Home:** This same person should plan to stay with you for the first 24 hours after you are discharged to help you and watch for any issues.   **4. The Day Before Your Procedure**  **The “Nothing by Mouth” Rule - This is for Your Safety!**   - **DO NOT EAT OR DRINK ANYTHING AFTER MIDNIGHT ([12:00 AM])** the night before your procedure. - This includes: all solid foods, water, coffee, juice, gum, and mints. - **Why?** The medicine we give you to relax can temporarily stop your body’s reflexes. If your stomach isn’t empty, its contents could go into your lungs, causing a serious infection. An empty stomach is a safe stomach.   **Hygiene:**   - Please shower or bathe the night before your procedure. Use regular soap and water. - Do not apply any lotions, deodorants, or powders to your body after showering.   **5. The Day of Your Procedure**  **At Home:**   - You may brush your teeth, but be careful not to swallow any water. - Wear loose, comfortable clothing that is easy to change out of. - **Do not wear or bring valuables.** Please leave all jewelry, rings, and watches at home.   **What to Bring:**   - Your photo ID and insurance information. - Your list of medications. - Eyeglasses or dentures (and their cases). - The name and phone number of your driver/caregiver for the day.   **At the Hospital:**   1. **Check-in:** You will register at the front desk. 2. **Getting Ready:** A nurse will take you to a preparation area, check your blood pressure and temperature, and ask you to change into a hospital gown. 3. **IV Line:** The nurse will place a small, soft plastic tube called an IV into a vein in your arm. This is how you will receive fluids and the relaxation medicine. 4. **Meeting Your Team:** You will meet the Interventional Radiologist who will perform the TACE, as well as the nurses and anesthesia staff who will be looking after you. This is the perfect time to ask any final questions. They will review your plan and ask you to confirm your name and the procedure you are having. 5. **Consent Form:** You will be asked to sign a form that says you understand the TACE procedure, its purpose, risks, and benefits. Please do not sign it until you feel all your questions have been answered.   **6. During and Immediately After Your TACE**  You will be taken to the procedure room and will lie on a special X-ray table. You will be given medicine through your IV to make you feel very sleepy and relaxed, a state called “conscious sedation” or “twilight sleep.” You will be comfortable and may not remember much, but you will not be under full general anesthesia. The team will be monitoring your heart, breathing, and blood pressure the entire time.  After the TACE is finished (it usually takes 1-2 hours), you will be moved to a recovery area.   - **Bed Rest:** You will need to lie flat for **[e.g., 4 to 6] hours**. This is very important to allow the small puncture site in your groin to heal and prevent any bleeding. - **Monitoring:** A nurse will be with you, checking on you frequently as you wake up.   **7. What to Expect After the Procedure (Potential Side Effects)**  It is very common to experience a group of symptoms called **“post-embolization syndrome.”** While they can feel like the flu, this is actually a sign that the treatment is working and the tumor tissue is breaking down. These symptoms can last for a few days to a week and may include:   - Abdominal pain or discomfort - Nausea or vomiting - Fever - Fatigue   We will give you medication to manage these symptoms and keep you comfortable.  **8. When to Call Us: Important Warning Signs**  Please call our office if you experience any of the following after you go home:   - A fever higher than 38.5°C (101.5°F) that is not helped by medication. - Pain or nausea that is severe and not controlled by your prescribed medicine. - Bleeding, swelling, or increasing pain at the puncture site in your groin.   **Go to the nearest Emergency Room if you have:**   - Sudden, severe shortness of breath or chest pain. - Heavy, uncontrolled bleeding from the groin site. - Sudden, severe abdominal pain that is different or worse than before. - Any signs of confusion or extreme weakness.   **Contact Information:**   - **For questions or concerns (daytime):** Interventional Radiology Clinic at [Clinic Phone Number] - **For urgent issues after hours:** Call the hospital operator at [Hospital Phone Number] and ask for the Interventional Radiologist on call.   Mr. Ma, we are fully committed to providing you with the best possible care. We will see you soon.  Sincerely,  Your Care Team at [Hospital Name] |
| 5.Case information: Patients scheduled for appendectomy |
| Answer:  Of course. Based on the detailed information provided for Mr. Chen, here is a comprehensive preoperative education guide tailored to his specific situation. This guide is written in clear, simple language, acknowledging the urgent nature of his surgery and his status as a first-time surgical patient.  **Your Guide to Preparing for Your Appendectomy**  **Patient Name:** Chen M. **Procedure:** Appendectomy (Surgical Removal of the Appendix) **Surgeon:** [Surgeon’s Name will be provided] **Location:** [Hospital Name and Floor/Unit]  Hello Mr. Chen,  We understand that coming to the hospital with severe pain and learning you need surgery can be stressful, especially when it happens so quickly. Please know that you are in excellent hands. Our entire team is here to ensure you are safe, comfortable, and well-informed.  An appendectomy is a very common and safe procedure performed to treat acute appendicitis, which is what our examinations show you have. This guide will walk you through what to expect in the hours ahead and after your surgery.  **1. What is Happening Now: Preparing for Your Urgent Surgery**  Because your surgery is urgent, we have already completed many of the initial steps in the Emergency Department.   - **Your Health Check:** The blood tests, ultrasound, and physical exam you already had were to confirm the diagnosis of appendicitis and ensure you are ready for surgery. All your results show that you are in good health, apart from the appendicitis. - **Your Personal History:** We see that you have no known allergies and do not smoke or drink. This is excellent and will help you recover more quickly. - **Fasting (Eating and Drinking):** This is the **most important safety rule** before surgery. To prevent complications while you are under anesthesia, **you must not eat or drink anything from this point forward.** A nurse will confirm with you the last time you had anything to eat or drink.   **2. About Your Surgery: The Appendectomy**  **What is happening:** Your appendix, a small tube attached to your large intestine, has become inflamed and infected. An appendectomy is the surgery to remove it, which will cure the infection and relieve your pain.  **Anesthesia:** You will be given **general anesthesia**. This means you will be completely asleep during the entire procedure. A specialist doctor, the Anesthesiologist, will give you medicine through your IV line and monitor you closely the entire time. You will not feel or remember the surgery.  **How the Surgery is Done:** Your surgeon will likely perform a **laparoscopic appendectomy**. This is a minimally invasive technique.   - **What it involves:** The surgeon will make 2-3 very small incisions (cuts) in your abdomen. A tiny camera (laparoscope) is inserted through one incision to see your appendix on a screen. Special, long, thin tools are used through the other incisions to carefully remove the appendix. - **Benefits:** This method usually means less pain, a shorter hospital stay, and a faster overall recovery. - **Alternative (Open Surgery):** In some cases, if the appendix is very swollen or has burst, the surgeon may need to make one larger incision in your lower right abdomen to remove it. Your surgeon will choose the safest and best method for you.   **3. Right Before You Go to the Operating Room**   1. **Final Preparations:** A nurse will help you get ready. You will be in a hospital gown. Please give any personal items like your phone, wallet, or watch to a trusted friend or family member for safekeeping. If you wear glasses, we will keep them safe for you. 2. **IV Line:** You will have an IV (a small, thin plastic tube) in your arm or hand. This is used to give you fluids to keep you hydrated and to administer the anesthesia medicine. 3. **Meeting Your Team:**    - **Your Surgeon** will visit you to answer any final questions you have. They will confirm the surgery plan with you and will likely use a special pen to mark the area of your surgery on your abdomen. This is a standard safety check.    - **Your Anesthesiologist** will also meet you. They will review your medical information and explain how they will keep you comfortable and asleep during the operation. 4. **The “Time-Out”:** When you arrive in the operating room, the entire team will pause for a safety check called a “time-out.” They will confirm your name, the procedure you are having, and the correct surgical site one last time. This happens for every single patient, every single time.   **4. After Your Surgery: In the Recovery Room**   - **Waking Up:** You will wake up in a special area called the Post-Anesthesia Care Unit (PACU), or the recovery room. A nurse will be right there with you, monitoring your breathing, heart rate, and blood pressure very closely. - **How You’ll Feel:** It’s normal to feel sleepy, groggy, and a bit chilly. Your throat might feel a little sore from the breathing tube used during anesthesia. - **Pain Management:** You will have some pain at the incision sites. This is expected. Your nurse will ask you to rate your pain on a scale from 0 (no pain) to 10 (the worst pain imaginable). **Please be honest about your pain.** We have medications to keep you comfortable, and it’s much easier to manage pain if we treat it early.   **5. Your Recovery in the Hospital and Going Home**   - **Moving to Your Room:** Once you are more awake and your condition is stable, you will be moved to a hospital room to continue your recovery. - **Eating and Drinking:** You will start with small sips of water. As you feel better, you will be able to have other clear liquids and then progress to solid food. - **Walking:** A nurse will help you get out of bed and walk as soon as it’s safe. Walking is very important—it helps prevent blood clots, reduces gas pain, and speeds up your recovery. - **Going Home:** Most patients can go home 1 or 2 days after an appendectomy. To be discharged, you must be able to drink fluids, have your pain controlled with oral medication, and be able to walk. - **Your Ride Home:** You **must** have a responsible adult drive you home. You will also need someone to stay with you for the first 24 hours after you leave the hospital. Please make arrangements with a family member or friend.   **6. Important Instructions for Your Recovery at Home**   - **Pain:** You will be given a prescription for pain medicine. Take it as directed. It’s often helpful to take it before the pain becomes severe. - **Incision Care:** Your small incisions will be covered with small bandages or surgical glue.   - Keep the area clean and dry. You can usually shower 24-48 hours after surgery. Let the water run over the incisions but do not scrub them. Pat them dry gently with a clean towel.   - Do not soak in a bathtub, hot tub, or go swimming until your doctor says it’s okay (usually after your follow-up appointment). - **Activity:**   - **Rest** when you feel tired.   - **Walk** a little more each day.   - **Do not lift** anything heavy (more than 5 kg or 10 lbs—about the weight of a large bag of rice) for at least 2-4 weeks.   - You can usually return to school or light desk work in about 1-2 weeks. Avoid strenuous exercise and sports for 4-6 weeks. Your surgeon will give you specific timelines. - **Diet:** You can return to your normal diet as you feel up to it. If your stomach feels upset, try bland, low-fat foods like rice, toast, or soup.   **When to Call Your Doctor or Seek Medical Help**  It is important to watch for signs of a possible complication. Please call your surgeon’s office or return to the hospital if you experience:   - **Fever** higher than 38.5°C (101°F). - **Increasing pain** in your abdomen that is not helped by your pain medication. - **Redness, swelling, or pus-like drainage** from your incisions. - **Vomiting** or being unable to keep liquids down. - **No bowel movement** or unable to pass gas for more than 3 days after surgery.   Mr. Chen, we are here to support you. Please do not hesitate to ask any nurse or doctor any questions you have.  **For any questions, please contact:**   - **Surgeon’s Office:** [Office Phone Number] - **After-Hours/Urgent Line:** [On-Call Number]   We are committed to providing you with the best possible care. |
| 6.Case information: Patients scheduled for transurethral resection of the prostate (TURP) |
| Answer:  Of course. Based on the detailed information you have provided for Mr. Meng, here is a comprehensive and easy-to-understand preoperative education guide.  **A Guide to Preparing for Your Prostate Surgery**  **Patient:** Mr. Meng M. **Procedure:** Transurethral Resection of the Prostate (TURP) **Admission Date:** July 18, 2025 **Location:** [Please Insert Hospital Name Here]  **A Personal Message for You, Mr. Meng**  Hello, Mr. Meng. This guide has been prepared by your medical team to help you and your family understand and prepare for your upcoming prostate surgery. We know that the last few years, and especially the last few days with the complete blockage, have been very difficult and uncomfortable for you. Our goal with this surgery is to relieve these symptoms so you can urinate freely again and improve your quality of life.  This is your first surgery, and it’s completely normal to have questions or feel a little anxious. We are here to support you every step of the way. Please read this guide carefully, and feel free to ask us any questions you may have.  **1. Understanding Your Surgery: What is a TURP?**  The formal name of your surgery is **Transurethral Resection of the Prostate (TURP)**. Let’s break down what that means in simple terms:   - **The Problem:** Your prostate gland, which sits just below the bladder, has grown larger over time. Think of your prostate as a donut and the tube you urinate through (the urethra) as the hole in the middle. As the “donut” has gotten bigger, it has squeezed the “hole” shut, making it very hard for urine to pass through. This is what caused your difficulty with urination and the recent blockage. - **The Solution (Your Surgery):** The surgeon will not make any cuts on the outside of your body. Instead, they will pass a special thin instrument through the natural opening of your penis into the urethra. This instrument has a light, a camera, and a small cutting loop. The surgeon will use this loop to carefully trim away the excess prostate tissue that is blocking the flow of urine, effectively making the “hole” in the “donut” wider again.   The main goal of this surgery is to create a clear channel for urine to pass, relieving your symptoms and preventing future blockages.  **2. The Weeks and Days Before Your Surgery**  **Your Medications:** It is very important that we know all the medications you take.   - **Blood Pressure Medicine:** You have a 10-year history of high blood pressure, and it is very well-controlled with your medication. This is excellent. **You should take your blood pressure medication on the morning of your surgery as usual, with just a small sip of water.** Keeping your blood pressure stable is important for your safety. - **Blood Thinners:** Please confirm with your nurse or doctor if you are taking any daily aspirin or other medications that thin the blood. If so, you will receive specific instructions on when to stop taking them before surgery. - **Other Pills and Supplements:** Please stop taking any herbal supplements, fish oil, or high-dose vitamins one to two weeks before surgery, as they can sometimes affect bleeding.   **Your Health and Well-being:**   - **Diet:** In the days leading up to surgery, focus on eating healthy meals and drinking plenty of water. This helps your body stay strong. - **Alcohol:** Please avoid drinking any alcohol for at least 48 hours before your surgery. - **Planning with Your Family:** You will be in the hospital for a few days. After you go home, you will need to rest and avoid strenuous activity. Please make arrangements with your family for help with transportation from the hospital and for support at home during your first week of recovery.   **3. The Day Before Your Surgery**  **Eating and Drinking - The Golden Rule:** This is the most important instruction for your safety. **DO NOT EAT OR DRINK ANYTHING AFTER MIDNIGHT the night before your surgery.** This includes water, tea, soup, candy, and gum.   - **Why is this so important?** An empty stomach is necessary for safe anesthesia. If there is food or liquid in your stomach, it could get into your lungs while you are asleep, causing very serious complications. For your safety, we must follow this rule strictly.   **Bathing:** You may be asked to shower with a special antibacterial soap the night before and/or the morning of your surgery. This helps reduce germs on your skin and lowers the risk of infection.  **4. The Day of Your Surgery: What to Expect**  **When You Arrive:**   - Please arrive at the hospital at the scheduled time. You will check in, and a nurse will then take you to a “pre-op” room. - You will change into a hospital gown. The nurse will check your blood pressure, heart rate, and temperature. - An IV (intravenous) line will be placed in your arm. This is a small, soft plastic tube that we use to give you fluids and medications to keep you comfortable and hydrated.   **Meeting Your Team:** Before you go to the operating room, you will meet the key people taking care of you:   - **Your Surgeon:** They will see you one last time to confirm the plan and answer any final questions. - **Your Anesthesiologist:** This is the doctor who will keep you safe and comfortable during the surgery. For a TURP, it is common to have **spinal anesthesia**. This involves an injection of numbing medicine into your lower back. It makes you completely numb from the waist down, so you feel no pain. You may be awake but relaxed and sleepy, or you may be given medicine to make you sleep lightly through the procedure. The anesthesiologist will discuss the best and safest plan with you.   **5. After Your Surgery: In the Hospital**  **In the Recovery Room:**   - After the surgery, you will be taken to a recovery room where a nurse will be with you constantly as the anesthesia wears off. You will feel sleepy. This is normal.   **The Urinary Catheter and Bladder Rinsing:** This is a very important part of your recovery.   - **You will wake up with a urinary catheter in place.** This is a soft tube that goes through your penis into your bladder to drain urine. It is essential because it allows the area to heal without you having to strain to urinate. - **Your urine will be pink or red.** This is completely normal and expected. It is from the small amount of bleeding where the prostate tissue was removed. - You will likely have **“bladder irrigation.”** This means a bag of sterile water will be connected to your catheter to gently and continuously rinse your bladder. This prevents blood clots from forming and blocking the catheter. You will see fluid flowing into and out of your catheter bag.   **Pain and Bladder Spasms:** It is common to feel some discomfort or a feeling like you need to urinate, even though the catheter is draining your bladder. These are called bladder spasms. Please tell your nurse if you are uncomfortable. We have medication that can help with this.  **Hospital Stay:** Most patients stay in the hospital for 1 to 3 days after a TURP. We will monitor you closely, manage the bladder irrigation, and decide when it is safe to remove the catheter.  **6. Going Home and Your Recovery**  **Activity:**   - **Rest is key.** Avoid any heavy lifting (nothing heavier than a jug of water), pushing, or straining for several weeks. - **Walking is good.** Short, gentle walks are encouraged to help your circulation and prevent blood clots. Avoid long car rides if possible.   **Urinating After the Catheter is Removed:**   - At first, you may experience some burning, a frequent need to urinate, or even leak a little urine. This is normal as your bladder re-learns how to control things. This will improve over the next few weeks. - You may still see some pink-tinged urine or small blood clots from time to time, especially after activity. This is normal.   **Diet and Fluids:**   - **Drink plenty of water!** This is very important. Aim for 8-10 glasses a day. It helps flush out your bladder, keeps your urine clear, and prevents infection. - Eat a diet high in fiber (fruits, vegetables, whole grains) to avoid constipation. Straining during a bowel movement can cause bleeding.   **7. When to Call Your Doctor or Seek Help**  Please call your doctor’s office if you experience:   - A fever over 101°F (38.3°C). - Your urine becomes thick with blood (like tomato soup) or you pass many large blood clots. - You are **completely unable to urinate** after the catheter is removed. - Pain that is severe and not controlled by your prescribed medication. - Cloudy or foul-smelling urine, which could be a sign of infection.   **Go to the Emergency Room if you have sudden chest pain, shortness of breath, or severe pain and swelling in one of your legs.**  **My Questions for the Team:**  Please use this space to write down anything you want to ask us.  Mr. Meng, we are all committed to providing you with the best possible care. We are confident that this procedure will bring you significant relief. We look forward to seeing you on your admission day. |
| Claude-Opus-4 |
| 1.Case information: Patients scheduled for coronary artery bypass grafting (CABG) |
| **Preoperative Education Guide for Your Heart Bypass Surgery**  **Dear Mr. Wang,**  You are scheduled for heart bypass surgery (CABG - Coronary Artery Bypass Grafting) to improve blood flow to your heart. This guide will help you understand and prepare for your surgery. Please read it carefully and ask us any questions you may have.  **What is Heart Bypass Surgery?**  Your heart needs blood to work properly. The blood vessels that supply your heart (coronary arteries) have become narrow due to buildup of fatty deposits. This is causing your chest pain and breathing problems.  During bypass surgery, your surgeon will:   - Take healthy blood vessels from another part of your body (usually your leg or chest) - Use these vessels to create new pathways around the blocked arteries - This allows blood to “bypass” the blockages and flow freely to your heart   **Before Your Surgery**  **Two Weeks Before Surgery**  **Medications to Review:**   - Continue taking your blood pressure medications as prescribed - Continue your diabetes medications as directed - We will give you specific instructions about which medications to stop and when - Do NOT stop any medications without talking to us first   **Important Health Steps:**   - Maintain good blood sugar control (keep checking your levels) - Continue avoiding smoking - you’re doing great having quit! - Eat healthy meals and get light exercise as tolerated - Get plenty of rest   **One Week Before Surgery**  **Pre-Surgery Testing** (if not already completed):   - Blood tests - Chest X-ray - Additional heart tests if needed   **Prepare Your Home:**   - Arrange for someone to stay with you for the first week after discharge - Place frequently used items at waist level to avoid bending - Remove throw rugs to prevent tripping - Prepare a comfortable sleeping area (you may prefer a recliner initially)   **Day Before Surgery**  **Eating and Drinking:**   - Eat a normal dinner (before 8 PM) - NO food or drinks after midnight - This includes water, candy, gum, or mints   **Personal Hygiene:**   - Take a shower using the special antibacterial soap we provide - Wash your entire body, especially the chest area - Do not use lotions, powders, or deodorant after showering - Sleep in clean clothes and clean bedding   **What to Pack:**   - Comfortable, loose-fitting clothes for going home - Slippers with non-slip soles - Glasses (not contact lenses) - Hearing aids if you use them - List of current medications - This education guide - Personal items (toothbrush, comb)   **Do NOT Bring:**   - Jewelry (including wedding rings) - Large amounts of money - Valuables   **Day of Surgery**  **Morning of Surgery**  **At Home:**   - Take another shower with the antibacterial soap - Do not eat or drink anything - Take only the medications we specifically told you to take with a tiny sip of water - Wear comfortable, loose clothing to the hospital   **Arrival at Hospital:**   - Arrive at: [Time will be provided] - Check in at: [Location will be provided] - Family members may accompany you   **Pre-Surgery Preparation**  **What Will Happen:**   1. You’ll change into a hospital gown 2. An IV (thin tube) will be placed in your arm for fluids and medications 3. We’ll check your vital signs (blood pressure, temperature, heart rate) 4. You’ll meet your surgical team 5. We’ll mark the surgical site 6. You’ll receive medications to help you relax   **For Your Family:**   - They can stay with you until you go to surgery - They’ll be directed to a waiting area - Surgery typically takes 3-5 hours - The doctor will update them when surgery is complete   **During Surgery**   - You’ll be completely asleep and won’t feel anything - A breathing tube will help you breathe - The surgical team will monitor you constantly - Your family will receive updates during the procedure   **After Surgery**  **Immediately After Surgery**  **Recovery Room (ICU):**   - You’ll wake up in the Intensive Care Unit - You’ll have several tubes and monitors - this is normal:   - Breathing tube (usually removed within 24 hours)   - Chest tubes to drain fluid   - Urinary catheter   - IV lines   - Heart monitor - You may feel groggy and confused at first - Pain medication will keep you comfortable   **What You Can Do:**   - Rest and let your body heal - Squeeze our hand or nod to communicate while the breathing tube is in - Take deep breaths when asked - Move your feet and legs as instructed   **First Few Days**  **Day 1-2:**   - Breathing tube removed (you’ll be able to talk) - Start sitting up in bed - Begin breathing exercises - May start clear liquids   **Day 3-4:**   - Most tubes removed - Start walking short distances - Regular diet as tolerated - Move to regular hospital room   **Day 5-7:**   - Walking in hallways - Preparing for discharge - Learning home care instructions   **Managing Your Recovery**  **Pain Control**  **What to Expect:**   - Some pain is normal after surgery - Pain is usually worst in the first few days - We’ll regularly ask about your pain level (0-10 scale)   **Pain Management:**   - Take pain medication as prescribed - Don’t wait until pain is severe - Use the breathing pillow we provide when coughing - Tell us if pain medication isn’t helping   **Breathing Exercises**  **Why They’re Important:**   - Prevent pneumonia - Help your lungs expand fully - Speed recovery   **How to Do Them:**   1. Take a slow, deep breath through your nose 2. Hold for 3 seconds 3. Slowly breathe out through your mouth 4. Do this 10 times every hour while awake 5. Use your incentive spirometer as directed   **Movement and Activity**  **In the Hospital:**   - Start slowly with help from staff - Gradually increase walking distance - Always call for help before getting up alone   **Wound Care:**   - Keep incisions clean and dry - We’ll teach you how to check for signs of infection - Don’t soak in a bath until cleared by your doctor   **Discharge Planning**  **Before You Go Home**  **We Will:**   - Review all medications - Teach wound care - Provide follow-up appointments - Answer all questions - Ensure you have help at home   **You Should Know:**   - Activity restrictions - Dietary guidelines - When to call the doctor - Cardiac rehabilitation schedule   **At Home**  **First Week:**   - Rest with light activity - Short walks several times daily - No lifting over 5 pounds - Someone should be with you   **Medications:**   - Take exactly as prescribed - Don’t skip doses - Keep a medication schedule - Continue monitoring blood sugar   **Diet for Heart Health:**   - Low salt (less than 2000mg daily) - Low fat, especially saturated fat - Fresh fruits and vegetables - Whole grains - Lean proteins - Control portion sizes - Monitor blood sugar levels   **Warning Signs - Call Your Doctor Immediately**  **Chest Issues:**   - Severe chest pain not relieved by medication - Difficulty breathing or shortness of breath - Coughing up blood   **Wound Problems:**   - Increased redness, swelling, or drainage - Wound opening up - Fever over 38°C (100.4°F) - Pus or bad smell from incision   **Other Concerns:**   - Irregular heartbeat or palpitations - Dizziness or fainting - Sudden weight gain (more than 1kg in a day) - Swelling in legs or feet - Confusion or changes in thinking   **Emergency:** Call 120 immediately for severe chest pain, difficulty breathing, or loss of consciousness.  **Frequently Asked Questions**  **Q: How long will I be in the hospital?** A: Usually 5-7 days if everything goes well.  **Q: When can I drive?** A: Not for 4-6 weeks, and only when cleared by your doctor.  **Q: When can I return to work?** A: This depends on your job. Desk work: 6-8 weeks. Physical work: 3 months.  **Q: Will I need cardiac rehabilitation?** A: Yes, this supervised exercise program will help you recover safely.  **Q: Can I climb stairs?** A: Yes, but go slowly and rest if needed. Limit to once or twice daily initially.  **Contact Information**  **Pre-Surgery Questions:**   - Surgical Coordinator: [Phone number] - Hours: Monday-Friday, 8:00-17:00   **After Discharge:**   - Cardiac Surgery Unit: [Phone number] - Available 24/7 for urgent concerns   **Follow-up Appointments:**   - Will be scheduled before discharge - Usually 1-2 weeks after surgery   **Notes Section**  Use this space to write down questions or important information:  **Remember:**   - We are here to help you through this process - No question is too small - Your recovery is our priority - Thousands of people have this surgery successfully each year   **We look forward to helping you return to a more active, comfortable life!**  *The Cardiac Surgery Team*  *This guide was prepared specifically for Mr. Wang M. on [Date]. Please bring this guide with you on your surgery day.* |
| 2.Case information: Patients scheduled for total knee arthroplasty (TKA) |
| Answer:  **Preoperative Education Guide for Your Knee Replacement Surgery**  **Dear Ms. Li,**  Welcome! This guide will help you prepare for your right knee replacement surgery (Total Knee Arthroplasty) scheduled at our hospital. We understand this may feel overwhelming, but we’re here to support you every step of the way.  **Understanding Your Surgery**  **What is Knee Replacement Surgery?**  Your surgeon will replace your damaged knee joint with an artificial joint made of metal and plastic. This will help:   - Relieve your knee pain - Improve your ability to walk - Correct the O-shaped curve in your leg - Help you return to daily activities without pain   **Why Do You Need This Surgery?**  After 10 years of knee problems and trying other treatments (medications, injections, physical therapy), surgery is now the best option to restore your quality of life and eliminate the need for a walking cane.  **Before Your Surgery - Important Preparation Steps**  **Two Weeks Before Surgery:**   1. **Continue Your Regular Medications:**    - Keep taking your blood pressure medicine as usual    - Continue your calcium tablets and vitamin D for osteoporosis    - Your doctor will tell you if any medications need to be stopped 2. **Home Preparation:**    - Remove loose rugs and clutter to prevent falls    - Arrange furniture for easy walking with a walker    - Set up a recovery area on the first floor if possible    - Install grab bars in the bathroom    - Get a raised toilet seat or shower chair 3. **Health Preparation:**    - Stay as active as your knee allows    - Eat healthy foods to boost healing    - Don’t smoke (if applicable)    - Avoid getting sick - wash hands frequently   **One Week Before Surgery:**   1. **Pre-Surgery Tests:**    - Complete any additional blood tests if requested    - Attend your pre-surgery appointment 2. **Plan Your Support:**    - Arrange for someone to stay with you for the first few days after surgery    - Organize transportation home from the hospital    - Prepare easy-to-make meals or arrange meal delivery   **The Day Before Surgery:**   1. **Bathing Instructions:**    - Take a shower with the special soap provided by the hospital    - Do not apply lotion, powder, or perfume after bathing    - Wear clean clothes to bed 2. **Eating and Drinking Rules:**    - Eat a normal dinner before 8 PM    - **DO NOT eat or drink anything after midnight** (including water, candy, or gum) 3. **Final Preparations:**    - Pack your hospital bag (see checklist below)    - Remove nail polish and jewelry    - Get a good night’s sleep   **Surgery Day Instructions**  **Morning of Surgery:**   1. **Hygiene:**    - Take another shower with the special soap    - Brush your teeth but don’t swallow any water    - Wear comfortable, loose clothing 2. **Medications:**    - Take ONLY the medications your doctor specifically told you to take with a tiny sip of water    - Bring all your regular medications to the hospital 3. **What to Bring:**    - ID card and insurance information    - List of medications and allergies (especially your penicillin allergy)    - Comfortable, loose shorts or pants    - Non-slip slippers with backs    - Glasses/hearing aids if you use them    - Walking cane    - Phone and charger    - This education guide 4. **What NOT to Bring:**    - Jewelry or valuables    - Large amounts of money    - Contact lenses (wear glasses instead)   **At the Hospital:**   1. **Check-in Process:**    - Arrive at the time given by your surgeon    - You’ll change into a hospital gown    - The nurse will start an IV line    - You’ll meet your anesthesia team 2. **Important Reminders:**    - Tell staff about your penicillin allergy    - Ask questions if you’re unsure about anything    - Your family can wait in the designated area   **What to Expect During Surgery**   1. **Anesthesia:**    - You’ll receive medicine to keep you comfortable    - You won’t feel any pain during surgery    - The surgery takes about 1-2 hours 2. **During Surgery:**    - Your surgical team will monitor you closely    - Your family will receive updates   **After Surgery - Recovery**  **Immediately After Surgery:**   1. **Recovery Room:**    - You’ll wake up with your new knee bandaged    - Nurses will monitor your vital signs    - You may feel groggy - this is normal 2. **Pain Management:**    - You’ll have pain medication to keep you comfortable    - Tell nurses if you have pain - don’t wait    - Ice packs will help reduce swelling   **First 24-48 Hours:**   1. **Movement:**    - Physical therapy starts the day after surgery    - You’ll learn to use a walker    - Getting up and moving prevents complications 2. **Daily Care:**    - Nurses will help you wash and dress    - You’ll wear special stockings to prevent blood clots    - Breathing exercises prevent lung problems   **Going Home - Usually 2-3 Days After Surgery**  **Discharge Criteria:**   - Walking safely with a walker - Pain controlled with pills - No signs of infection - Can do basic exercises   **Home Care Instructions:**   1. **Wound Care:**    - Keep bandage clean and dry    - Watch for signs of infection    - Follow specific wound care instructions 2. **Activity:**    - Continue exercises as taught    - Gradually increase walking    - No driving until doctor approves 3. **Medications:**    - Take pain medicine as directed    - Continue blood pressure medicine    - Take prescribed blood thinners to prevent clots   **Warning Signs - Call Your Doctor Immediately If:**   - **Fever over 38°C (100.4°F)** - **Increasing redness, warmth, or drainage from wound** - **Severe pain not relieved by medication** - **Chest pain or trouble breathing** - **Calf pain, redness, or swelling** - **Sudden increase in knee swelling** - **Feeling confused or unusually tired**   **Follow-Up Care**   - **First appointment:** 10-14 days after surgery (remove stitches) - **Physical therapy:** 2-3 times per week for several weeks - **Regular check-ups:** 6 weeks, 3 months, 6 months, then yearly   **Recovery Timeline**   - **Week 1-2:** Focus on wound healing and basic movement - **Week 3-6:** Increase activity, may stop using walker - **Week 6-12:** Return to most normal activities - **3-6 months:** Full recovery for most patients   **Frequently Asked Questions**  **Q: Will I have pain after surgery?** A: Yes, but we’ll manage it with medication. Most patients say the surgery pain is less than their arthritis pain.  **Q: When can I shower?** A: Usually 2-3 days after surgery with wound protection. Your nurse will show you how.  **Q: When can I drive?** A: Usually 4-6 weeks, when you can bend your knee enough and aren’t taking strong pain medicine.  **Q: How long will my new knee last?** A: Most knee replacements last 15-20 years or longer with good care.  **Contact Information**  **Hospital Main Number:** [Will be provided] **Orthopedic Clinic:** [Will be provided] **Emergency Department:** [Will be provided]  **Call your doctor’s office for:**   - Questions about medications - Concerns about your wound - Problems with pain control   **Go to Emergency Room for:**   - Chest pain or trouble breathing - Signs of blood clot - High fever with chills   **Final Reminders**   1. **Stop eating and drinking after midnight before surgery** 2. **Tell us about your penicillin allergy** 3. **Bring this guide to the hospital** 4. **Ask questions - we’re here to help!**   **Notes Section** (Write your questions here):  We look forward to helping you through this journey to a pain-free, more active life. Your surgical team is experienced and committed to your safety and success.  **See you on your surgery day!**  *The Orthopedic Surgery Team* |
| 3.Case information: Patients scheduled for laparoscopic cholecystectomy (LC) |
| Answer:  **Preoperative Education Guide for Your Gallbladder Surgery**  **Patient Name:** Zhang M. **Surgery Date:** To be scheduled **Surgery Type:** Laparoscopic Cholecystectomy (Minimally Invasive Gallbladder Removal)  **Welcome, Ms. Zhang!**  This guide will help you understand and prepare for your upcoming gallbladder surgery. We want to make sure you feel comfortable and well-informed about what to expect. Please read through this information carefully and don’t hesitate to ask questions.  **Why Do You Need This Surgery?**  You have been diagnosed with:   - **Gallstones** (stones in your gallbladder) - **Acute cholecystitis** (inflammation of your gallbladder)   Your gallbladder has multiple stones, with the largest being about 1.5 cm. These stones are causing your recurring right upper belly pain, nausea, and vomiting. Surgery is the best way to prevent future attacks and complications.  **About Your Surgery**  **What is Laparoscopic Cholecystectomy?**   - A minimally invasive surgery to remove your gallbladder - Uses 3-4 small incisions (cuts) instead of one large incision - A tiny camera and special instruments are used - Usually takes 30-60 minutes - Most patients go home the same day or next day   **Benefits of This Approach:**   - Less pain after surgery - Smaller scars - Faster recovery - Shorter hospital stay - Quick return to normal activities   **Before Your Surgery Day**  **7 Days Before Surgery:**   - Continue your normal medications unless told otherwise - Stop smoking if you smoke (this helps healing) - Avoid alcohol - Stay active but avoid strenuous exercise - Eat healthy foods to strengthen your body   **2 Days Before Surgery:**   - Shower daily with regular soap - Remove nail polish and artificial nails - Arrange for someone to drive you home after surgery - Prepare your home for recovery (easy-to-reach items, comfortable resting area)   **1 Day Before Surgery:**   - You may eat normally until midnight - After midnight: **NO food or drink** (not even water) - Get a good night’s sleep - Shower with antibacterial soap if provided   **Day of Surgery**  **What to Bring:**   - Your ID card and insurance information - List of current medications - Comfortable, loose-fitting clothes - Slip-on shoes (no laces) - Glasses instead of contact lenses - Leave jewelry, valuables, and money at home   **Morning of Surgery:**   - Do NOT eat or drink anything - Take only approved medications with a tiny sip of water - Shower but do not use lotions, perfumes, or deodorants - Arrive at the hospital 2 hours before your scheduled time   **What Will Happen:**   1. **Check-in and Preparation** (1-2 hours)    - Change into a hospital gown    - Nurses will check vital signs    - IV line placed in your arm    - Meet your surgical team    - Sign consent forms 2. **Anesthesia**    - You’ll receive general anesthesia (you’ll be asleep)    - A breathing tube will be placed after you’re asleep    - You won’t feel or remember anything 3. **Surgery** (30-60 minutes)    - Small incisions made in your belly    - Gallbladder carefully removed    - Incisions closed with stitches or skin glue 4. **Recovery Room** (1-2 hours)    - Wake up gradually    - Nurses monitor you closely    - May feel groggy or have mild discomfort    - Once stable, move to regular room   **After Surgery**  **In the Hospital:**   - Start with ice chips and clear liquids - Gradually progress to regular food - Walk as soon as possible (prevents complications) - Pain medication available as needed - Most patients go home same day or next morning   **At Home - First Week:**   - **Pain Management:**   - Take prescribed pain medications as directed   - Use ice packs on incisions (20 minutes at a time)   - Rest when tired - **Activity:**   - Walk several times daily (start with 5-10 minutes)   - No lifting over 5 kg (11 pounds)   - No driving while taking pain medication   - Can shower after 24-48 hours - **Diet:**   - Start with light, low-fat foods   - Eat small, frequent meals   - Avoid greasy, fried, or spicy foods initially   - Drink plenty of water   - Gradually return to normal diet   **Incision Care:**   - Keep incisions clean and dry - Shower normally after 48 hours - Pat dry gently - No baths or swimming for 2 weeks - Small strips on incisions will fall off naturally   **Recovery Timeline**   - **Day 1-3:** Rest at home, light activities - **Day 4-7:** Increase walking, light household tasks - **Week 2:** Return to work (office job), normal daily activities - **Week 3-4:** Resume exercise gradually - **Week 4-6:** Full recovery for most people   **Warning Signs - Call Your Doctor If You Have:**  **Call Immediately for:**   - Fever over 38°C (100.4°F) - Severe pain not helped by medication - Persistent vomiting - Yellowing of skin or eyes - Difficulty breathing - Chest pain   **Also Contact for:**   - Increasing redness or drainage from incisions - Swelling that gets worse - No bowel movement for 3 days - Severe bloating - Any concerns or questions   **Living Without a Gallbladder**   - Your liver will still make bile for digestion - Most people have no long-term problems - Some may have looser stools initially - Can eat normally after recovery - No special diet required long-term   **Questions to Ask Your Surgical Team:**   1. How many of these surgeries have you performed? 2. What are the specific risks for me? 3. When can I return to work? 4. What pain medications will I receive? 5. When is my follow-up appointment?   **Important Phone Numbers:**  **Hospital Main Number:** [To be provided] **Surgical Department:** [To be provided] **Emergency Department:** [To be provided] **After-Hours Nurse Line:** [To be provided]  **Final Reminders:**  ✓ Stop eating and drinking at midnight before surgery ✓ Arrange transportation home ✓ Follow all preoperative instructions ✓ Bring this guide with you ✓ Ask questions if anything is unclear  **Notes Section:**  *Use this space to write down questions or important information from your surgical team*  **We are committed to providing you with excellent care. Your safety and comfort are our top priorities. We look forward to helping you feel better soon!**  *This guide was prepared specifically for Ms. Zhang M. based on her medical condition and planned surgery. Please follow all specific instructions given by your surgical team.* |
| 4.Case information: Patients scheduled for transcatheter arterial chemoembolization (TACE) |
| Answer:  **Preoperative Education Guide for TACE Procedure**  **Patient Name:** Ma M. **Date of Procedure:** To be scheduled **Procedure:** Transcatheter Arterial Chemoembolization (TACE)  **Welcome, Mr. Ma**  This guide will help you understand and prepare for your upcoming TACE procedure. We’ve written everything in simple terms to make it easy to understand. Please read through this carefully, and don’t hesitate to ask questions.  **What is TACE?**  TACE is a treatment for your liver tumor. Here’s how it works:   - A thin tube (catheter) is inserted through a small cut in your groin area - The doctor guides this tube to the blood vessels that feed your tumor - Medicine to fight cancer and tiny particles are delivered directly to the tumor - This blocks the tumor’s blood supply and delivers medicine right where it’s needed   **Why TACE for you?** Based on your test results, TACE is a good treatment option because:   - Your tumor is located in one area of your liver - Your liver is still working reasonably well despite the cirrhosis - This treatment can help control the tumor and improve your symptoms   **Before Your Procedure**  **Two Weeks Before**   - **Continue your regular medications** unless we tell you otherwise - **Maintain good nutrition** - eat balanced meals to keep your strength up - **Stay hydrated** - drink plenty of water daily   **One Week Before**   - **Blood tests** - You’ll need to come in for blood work to check:   - Blood cell counts   - Liver function   - Blood clotting ability - **Stop certain medications** if instructed (we’ll give you a specific list) - **Arrange transportation** - You’ll need someone to drive you home after the procedure   **Two Days Before**   - **Confirm your ride** home - **Prepare your home** for recovery:   - Stock easy-to-prepare meals   - Have extra pillows for comfortable positioning   - Clear walkways to prevent falls   **Day Before Your Procedure**  **Eating and Drinking:**   - Eat normally during the day - Have a light dinner before 8 PM - **STOP eating at midnight** - no solid food after 12:00 AM - You may have clear liquids (water, clear tea, clear broth) until 4 hours before your procedure   **Medications:**   - Take your regular medications with a small sip of water unless told otherwise - If you take medications for hepatitis B, continue taking them   **Personal Care:**   - Take a shower using regular soap - Remove nail polish and jewelry - Get a good night’s sleep   **Day of Your Procedure**  **What to Bring**  ✓ Your ID card and insurance information ✓ List of all your medications ✓ Comfortable, loose clothing ✓ Slippers or comfortable shoes ✓ Your glasses (remove contact lenses) ✓ Any medical devices you use (hearing aids, dentures)  **What NOT to Bring**  ✗ Jewelry or valuable items ✗ Large amounts of money ✗ Your own medications (we’ll provide what you need)  **Morning of Procedure**   1. **Don’t eat or drink anything** (unless instructed otherwise) 2. Take only medications approved by your doctor with tiny sips of water 3. Shower with regular soap 4. Wear comfortable, loose clothing 5. Arrive at the hospital at your scheduled time   **During Your Procedure**  **What Will Happen**   1. **Check-in** - The nursing staff will:    - Check your vital signs (blood pressure, temperature, heart rate)    - Start an IV line for fluids and medications    - Ask you to change into a hospital gown 2. **Preparation**    - You’ll lie on a special X-ray table    - The groin area will be cleaned and numbed    - You’ll receive medicine to help you relax (conscious sedation)    - You’ll be awake but drowsy and comfortable 3. **The Procedure**    - Takes about 1-2 hours    - You may feel some pressure but shouldn’t feel pain    - The doctor will insert a thin tube through your groin    - X-ray images guide the tube to your liver    - Medicine is delivered directly to the tumor 4. **After Completion**    - The tube is removed    - Pressure is applied to the insertion site to prevent bleeding    - You’ll need to lie flat for 4-6 hours   **After Your Procedure**  **Immediate Recovery (First 24 Hours)**   - **Stay in bed** - Lie flat for 4-6 hours to prevent bleeding - **Keep your leg straight** where the tube was inserted - **Expect some discomfort**:   - Mild pain at the insertion site   - Some nausea or stomach discomfort   - Low-grade fever (up to 101°F/38.3°C)   - Fatigue   **Going Home**  Most patients go home the next day. Before leaving:   - We’ll check your vital signs - The insertion site will be examined - You’ll receive detailed home care instructions - Prescriptions for any needed medications   **Home Recovery Instructions**  **First Week at Home**  **Activity:**   - Rest is important - your body needs energy to heal - No heavy lifting (nothing over 10 pounds/5 kg) for one week - No strenuous activities or exercise - Short walks are encouraged to prevent blood clots - Gradually increase activity as you feel better   **Diet:**   - Start with light, easy-to-digest foods - Eat small, frequent meals if you have nausea - Drink plenty of fluids - Avoid alcohol completely - Limit salty foods   **Wound Care:**   - Keep the bandage on for 24 hours - After removing the bandage, keep the area clean and dry - You may shower after 48 hours - Don’t soak in a bathtub or swim for one week   **Medications:**   - Take prescribed pain medication as needed - Continue your hepatitis B medication - Take anti-nausea medication if prescribed - Don’t take aspirin or blood thinners unless approved   **Common Side Effects (Normal)**  These symptoms are common and usually improve within a week:   - Mild fever (up to 101°F/38.3°C) - Fatigue and weakness - Poor appetite - Mild nausea - Slight pain in the upper right abdomen - Small bruise at the insertion site   **When to Call Your Doctor**  **Call Immediately If You Have:**  🚨 **Emergency Signs:**   - High fever (over 101.5°F/38.6°C) - Severe abdominal pain - Persistent vomiting - Bleeding that won’t stop at the insertion site - Large, growing bruise at the groin - Leg becomes cold, numb, or changes color - Difficulty breathing or chest pain - Confusion or difficulty staying awake - Yellow skin or eyes (jaundice)   **Call During Office Hours For:**   - Mild symptoms that don’t improve after 3 days - Questions about medications - Concerns about your recovery   **Emergency Contact:** [Hospital Emergency Number] **Doctor’s Office:** [Office Number]  **Follow-Up Care**  **First Follow-Up Visit (1-2 weeks)**   - Physical examination - Blood tests to check liver function - Discussion of how you’re feeling - Wound check   **Imaging Follow-Up (4-6 weeks)**   - CT or MRI scan to check treatment response - Additional blood tests including AFP levels   **Long-Term Follow-Up**   - Regular imaging every 3-4 months - Blood tests to monitor liver function - Assessment for additional treatments if needed   **Frequently Asked Questions**  **Q: Will I feel pain during the procedure?** A: You’ll receive numbing medicine and sedation. Most patients feel pressure but not pain.  **Q: How long until I can return to normal activities?** A: Most patients return to light activities within a week and normal activities within 2-3 weeks.  **Q: Will I need more than one treatment?** A: Many patients need 2-3 TACE treatments. We’ll evaluate after your first treatment.  **Q: Can I drive after the procedure?** A: No driving for 24 hours after sedation. Avoid driving for 2-3 days while recovering.  **Q: What about my hepatitis B?** A: Continue your hepatitis B monitoring and medications. TACE doesn’t cure hepatitis B.  **Q: Will my hair fall out?** A: TACE delivers chemotherapy directly to the tumor, so hair loss is very rare.  **Q: Can I eat normally afterward?** A: Start with light foods and gradually return to your normal diet as tolerated.  **Important Reminders**  ✓ **Stop eating at midnight** before your procedure ✓ **Arrange transportation** - you cannot drive after sedation ✓ **Follow medication instructions** carefully ✓ **Keep all follow-up appointments** ✓ **Call if you have concerns** - we’re here to help  **Notes Section**  Use this space to write down questions for your healthcare team:  **We’re here to support you through this treatment. Don’t hesitate to contact us with any questions or concerns.**  **Your Healthcare Team** [Hospital Name] [Department] [Contact Information]  *This guide was prepared specifically for Mr. Ma M. on [Date]. Please bring this guide with you on the day of your procedure.* |
| 5.Case information: Patients scheduled for appendectomy |
| Answer:  **PREOPERATIVE EDUCATION GUIDE**  **Patient Name:** Chen M. **Surgery:** Appendectomy (Appendix Removal) **Date:** To be scheduled  **Welcome, Chen M.**  You are scheduled for surgery to remove your appendix. This guide will help you understand what to expect before, during, and after your surgery. Please read this carefully and ask us any questions you may have.  **WHAT IS AN APPENDECTOMY?**  An appendectomy is a surgery to remove your appendix - a small, finger-shaped pouch attached to your large intestine in the lower right side of your belly. Your appendix has become inflamed and infected (appendicitis), which is why you’ve been experiencing pain. Removing it will eliminate the infection and prevent serious complications.  **BEFORE YOUR SURGERY**  **Pre-Surgery Testing ✓**  Your blood work and other tests have been completed. No additional testing is needed unless your doctor orders new tests.  **Medications**   - **STOP** taking any blood-thinning medications if you take them (like aspirin) - your doctor will tell you when to stop - **CONTINUE** taking your regular medications unless told otherwise - **INFORM** us about ALL medications and supplements you take   **The Night Before Surgery**   - **Shower** with regular soap - **Do NOT eat or drink anything after midnight** (not even water, gum, or candy) - **Get a good night’s sleep** - **Remove** all jewelry, including piercings   **The Morning of Surgery**   - **Do NOT eat or drink anything** - **Brush your teeth** but don’t swallow any water - **Shower** again if instructed - **Wear** comfortable, loose clothing that’s easy to remove - **Leave at home:** jewelry, money, credit cards, and valuables   **DAY OF SURGERY**  **What to Bring**   - Your ID card - Insurance information - This education packet - Glasses (no contact lenses) - A family member or friend to drive you home   **What Will Happen**   1. **Check-in** at the surgical reception desk 2. **Change** into a hospital gown 3. **Meet** your surgical team (surgeon, anesthesiologist, nurses) 4. **IV line** will be placed in your arm for fluids and medications 5. **Move** to the operating room   **About Your Anesthesia**  You will receive **general anesthesia**, which means:   - You’ll be completely asleep during surgery - You won’t feel any pain - You won’t remember the surgery - A breathing tube will help you breathe while asleep   **Surgery Details**   - **Duration:** About 30-60 minutes - **Method:** Usually done through small incisions (laparoscopic) or one larger incision - Your surgeon will decide the best approach for you   **AFTER YOUR SURGERY**  **In the Recovery Room**   - You’ll wake up gradually (30-60 minutes) - Nurses will monitor your vital signs - You may feel groggy, have a sore throat, or feel nauseated - Pain medication will be given as needed - Once fully awake, you’ll return to your room   **Pain Management**   - **Expect** some pain at the incision site(s) - **Pain scale:** We’ll ask you to rate your pain from 0-10 - **Medications:** We’ll give you pain medicine to keep you comfortable - **Tell us** if your pain isn’t controlled   **Before Going Home**  You must be able to:   - Wake up easily - Drink fluids without vomiting - Urinate - Walk with assistance - Have your pain under control   **HOME CARE INSTRUCTIONS**  **Activity**   - **Rest** for the first 24-48 hours - **Walk** short distances several times daily to prevent blood clots - **No heavy lifting** (over 10 pounds) for 2 weeks - **No strenuous exercise** for 2 weeks - **Return to school** when you feel ready (usually 1-2 weeks)   **Incision Care**   - Keep incisions **clean and dry** - **Shower** after 24-48 hours (let water run over incisions, pat dry) - **No baths or swimming** for 2 weeks - **Small bandages** may be used if there’s drainage - **Stitches/staples** will dissolve or be removed at follow-up   **Diet**   - **Start with** clear liquids (water, tea, broth) - **Progress to** soft foods as tolerated - **Resume normal diet** gradually - **Avoid** spicy or greasy foods initially - **Drink plenty of water**   **Medications at Home**   - Take prescribed pain medications as directed - You may also use over-the-counter pain relievers as recommended - Complete any prescribed antibiotics   **WHEN TO CALL YOUR DOCTOR**  Call immediately if you experience:   - **Fever** over 38.5°C (101.3°F) - **Severe pain** not relieved by medication - **Persistent vomiting** - **Redness, swelling, or pus** at incision sites - **Bleeding** from incisions - **Difficulty urinating** - **Shortness of breath** or chest pain - **Leg pain or swelling**   **FOLLOW-UP CARE**   - **First appointment:** 1-2 weeks after surgery - **Location:** Surgical clinic - **Purpose:** Check incisions, remove stitches/staples if needed - **Bring:** List of questions or concerns   **CONTACT INFORMATION**  **Your Surgical Team:**   - Weekdays (8:00-17:00): [Hospital phone number] - After hours/weekends: [Emergency number] - Emergency: Call 120 or go to nearest emergency room   **Important Reminders:**   - This surgery is very common and safe - Most people recover completely within 2-4 weeks - Following these instructions helps ensure the best recovery   **QUESTIONS FOR YOUR HEALTHCARE TEAM**  Use this space to write down questions:  **We are here to help you through this process. Please don’t hesitate to ask questions or express any concerns you may have.**  **Signature of patient indicating understanding:**  Chen M. Date: ___________  **Healthcare provider signature:**  Provider Name Date: ___________ |
| 6.Case information: Patients scheduled for transurethral resection of the prostate (TURP) |
| Answer:  **Preoperative Education Guide for Your TURP Surgery**  **Patient Name:** Mr. Meng M. **Surgery:** Transurethral Resection of the Prostate (TURP) **Scheduled Date:** To be confirmed  **Dear Mr. Meng,**  This guide will help you understand and prepare for your upcoming prostate surgery. Please read it carefully and share it with your family. If you have any questions, please ask your healthcare team.  **What is TURP Surgery?**  TURP is a procedure to treat your enlarged prostate. Your doctor will remove part of your prostate gland that is blocking your urine flow. This is done through your urethra (the tube you urinate through), so there are no cuts on the outside of your body.  **Why do you need this surgery?**   - Your prostate has grown larger over the years (like a duck egg instead of a walnut) - This has blocked your ability to urinate normally - The surgery will help you urinate freely again   **Before Your Surgery**  **One Week Before Surgery:**   1. **Continue your blood pressure medication** as usual 2. **Tell your doctor** about all medications you take 3. **Stop blood-thinning medications** if instructed (your doctor will tell you which ones) 4. **Arrange transportation** - you cannot drive after surgery   **Day Before Surgery:**   1. **Eating and Drinking:**    - Eat a normal dinner before 8 PM    - NO food or drink after midnight (including water, candy, or gum) 2. **Personal Hygiene:**    - Take a shower with regular soap    - Trim your fingernails    - Remove nail polish if you wear it 3. **At Home:**    - Get a good night’s sleep    - Set two alarms to wake up on time   **Day of Surgery**  **What to Bring:**   - Your ID card and insurance information - List of your medications - Comfortable, loose clothing - Slippers with non-slip soles - Personal items (toothbrush, comb) - This education guide   **What NOT to Bring:**   - Jewelry, watches, or valuables - Large amounts of money - Contact lenses (wear glasses instead)   **Morning of Surgery:**   1. Take your blood pressure medication with a small sip of water (only if instructed) 2. Brush your teeth but don’t swallow water 3. Arrive at the hospital 2 hours before your scheduled time   **What Happens During Surgery**   1. **Check-in:** Nurses will check your vital signs and start an IV line 2. **Anesthesia:** You’ll receive medication to make you sleep during surgery 3. **The Procedure:** Takes about 60-90 minutes    - A thin instrument is passed through your penis    - Extra prostate tissue is removed    - Your catheter (urine tube) stays in place after surgery 4. **Recovery Room:** You’ll wake up here with nurses monitoring you   **After Your Surgery**  **In the Hospital (1-2 days):**   - You’ll have a catheter draining urine from your bladder - The urine may be pink or have some blood - this is normal - You’ll receive pain medication as needed - Start drinking water when allowed - Begin walking as soon as possible   **Going Home:**  You can go home when:   - Your urine is clearing up - You’re drinking and eating normally - Your pain is controlled - Your blood pressure is stable   **Recovery at Home**  **First 2 Weeks:**  **DO:**   - Drink 8-10 glasses of water daily - Walk around your home regularly - Take prescribed medications - Rest when tired - Shower normally   **DON’T:**   - Lift anything heavier than 5 kg (about a watermelon) - Drive until cleared by your doctor - Strain during bowel movements - Drink alcohol - Have sexual activity   **Catheter Care (if you go home with one):**   - Keep the drainage bag below your bladder level - Empty the bag when it’s half full - Wash your hands before and after handling - Keep the catheter taped to your leg   **What to Expect**  **Normal After Surgery:**   - Pink or light red urine for several weeks - Burning sensation when urinating - Frequent urge to urinate - Small blood clots in urine - Feeling tired   **Call Your Doctor If You Have:**   - Bright red bleeding that doesn’t stop - Fever above 38°C - Cannot urinate - Severe pain not helped by medication - Large blood clots blocking your catheter - Chest pain or trouble breathing   **Follow-up Care**   - **First appointment:** Usually 1-2 weeks after surgery - **What to expect:** Catheter removal (if still in place) - **Bring:** List of questions, medication list   **Long-term Recovery**  **Weeks 3-6:**   - Gradually increase activities - May return to work if it’s not physically demanding - Continue drinking plenty of water   **After 6 weeks:**   - Most normal activities can resume - Sexual activity may resume (discuss with doctor) - Heavy lifting and strenuous exercise allowed   **Important Reminders**   1. **Your blood pressure medication:** Continue taking as prescribed 2. **Preventing infection:** Keep your genital area clean 3. **Bowel movements:** Avoid straining - use stool softeners if needed 4. **Follow-up PSA test:** Will be scheduled in 3-6 months   **Contact Information**  **Hospital Ward:** [To be provided] **Emergency Number:** [To be provided] **Your Surgeon:** [To be provided] **Ward Nurse Station:** [To be provided]  **Call immediately for:**   - Heavy bleeding - High fever - Severe pain - Cannot urinate - Chest pain or breathing problems   **Questions to Ask Your Doctor**  Write your questions here:  **Notes for Your Family**  Your family plays an important role in your recovery:   - Help remind about medications - Encourage drinking water - Assist with daily activities - Watch for warning signs - Provide emotional support   **Remember:** This surgery has helped many men return to normal urination. Following these instructions will help ensure your best recovery.  **We are here to help you. Please don’t hesitate to ask questions!**  *This guide was prepared specifically for Mr. Meng M. Please bring it with you on the day of surgery.*  *Date prepared: [Current date]* |
| Grok-4 |
| 1.Case information: Patients scheduled for coronary artery bypass grafting (CABG) |
| Answer:  **Preoperative Education Guide for Your Heart Bypass Surgery**  Dear Mr. Wang,  Hello! My name is [Your AI Assistant acting as a medical educator], and I’m here to help you understand what to expect before, during, and after your heart bypass surgery (also called CABG, which stands for coronary artery bypass grafting). This guide is based on your health details, like your age (68 years old), your history of heart issues, high blood pressure, diabetes, high cholesterol, and your past smoking. We’ve made this easy to read, using simple words instead of hard medical terms. Remember, this is general information to help you prepare—it’s not a replacement for talking to your doctor or surgeon. Always follow their specific advice, and ask them any questions you have.  We’ll cover everything step by step: why you’re having this surgery, how to get ready, what happens on surgery day, the surgery itself, possible risks, recovery, and tips for a healthier life afterward. Let’s get started!  **1. Why You Need This Surgery and What It Is**  Your heart needs good blood flow to work well. Over time, the tubes that carry blood to your heart (called arteries) can get narrow or blocked because of buildup from things like high cholesterol, smoking, diabetes, and high blood pressure. This has caused you chest pain, shortness of breath, and other problems for the last two years.   - **Your specific situation**: Tests show three of your heart’s main arteries are narrowed or blocked (one is fully blocked at the end). This is why you’ve had pain even when resting, and why your heart isn’t pumping as strongly as it could (it’s at about 55% strength, which is okay but needs help). - **What the surgery does**: The surgeon will create new paths for blood to flow around the blocked areas. They’ll use healthy blood vessels from other parts of your body (like your chest, arm, or leg) to “bypass” the blocks. This helps your heart get more oxygen and reduces pain and risks like heart attacks. - **Why now?** Your symptoms are getting worse, and medicines or other treatments aren’t enough. This surgery can improve your daily life, help you breathe easier, and lower your chance of future heart problems.   **2. Getting Ready Before Surgery (Preoperative Preparation)**  Preparation starts now and goes until the day before surgery. It helps make the surgery safer and your recovery smoother. Based on your history (like diabetes, high blood pressure, and past smoking), we’ll focus on controlling those.  **Health Checks and Tests**   - You’ve already had tests like blood work, heart scans, and artery imaging. If needed, you might have more, like lung checks or another heart rhythm test. - **For your diabetes**: Your blood sugar is a bit high (fasting level at 8.9 and long-term measure at 7.8%). Work with your doctor to keep it steady—maybe adjust your pills or add insulin temporarily. - **For high blood pressure**: It’s at 145/88 now, which is better than your past highs, but keep taking your pills. Aim for calm activities to help lower it. - **For high cholesterol**: Levels are elevated—talk to your doctor about starting or changing medicines. - **Other checks**: Tell your team about any allergies (you have none noted), recent illnesses, or family history (like your dad’s heart issues and mom’s conditions—these raise your risks, so they’re watching closely).   **Medicines and What to Stop**   - Keep taking your usual pills for blood pressure, diabetes, and heart unless told otherwise. - You might need to stop blood-thinning medicines (if you’re on any) a few days before to reduce bleeding risks. - If you have pain, use the under-tongue spray (nitroglycerin) as before, but tell your doctor about any changes. - No new over-the-counter drugs, vitamins, or herbs without asking—some can affect surgery.   **Lifestyle Changes**   - **Smoking**: Great job quitting a year ago! Stay smoke-free—it helps your lungs and heart heal better. - **Alcohol**: Keep it to occasional or none, as it can interact with medicines. - **Diet**: Eat light, healthy meals low in salt, sugar, and fats. Focus on fruits, veggies, lean proteins, and whole grains to manage your diabetes and cholesterol. Avoid big meals the night before surgery. - **Exercise**: Light walks if you can, but nothing strenuous. Rest if you feel chest pain or short of breath. - **Weight**: If overweight, even small losses can help, but don’t start a strict diet now—talk to your doctor. - **Hygiene**: Shower with antibacterial soap the night before and morning of surgery to lower infection risks. - **Fasting**: No food or drink after midnight before surgery day (except sips of water with pills if allowed).   **Emotional and Practical Prep**   - It’s normal to feel worried. Talk to family, friends, or a counselor. Relaxation techniques like deep breathing can help. - Plan for help at home after surgery—your wife or family can assist. - Pack a bag with comfy clothes, toiletries, glasses, and any needed devices (like hearing aids). - Arrange transport to/from the hospital.   **3. What to Expect on Surgery Day**   - Arrive early (on July 10th or as scheduled). You’ll check in, change into a gown, and meet the team. - An IV line (small tube in your arm) will give fluids and medicines. - You’ll get medicine to relax you, then anesthesia to make you sleep deeply and feel no pain during surgery. - Family can wait in a designated area.   **4. During the Surgery**   - It usually takes 3-6 hours, depending on how many bypasses you need (likely 3 based on your artery blocks). - The surgeon makes a cut down the middle of your chest to reach your heart. They might stop your heart briefly and use a machine to pump blood (heart-lung machine), or do it while your heart beats. - They’ll take healthy vessels and attach them to bypass the blocks. - After, your chest is closed with stitches or staples, and you’ll go to a special care unit.   **5. Possible Risks and Complications**  All surgeries have risks, but your team will watch closely. Based on your age, diabetes, high blood pressure, and heart condition, some risks are higher, but steps are taken to minimize them.   - Common: Pain, tiredness, or swelling where cuts were made. - Others: Infection, bleeding, irregular heartbeat, breathing issues, or kidney strain (your kidneys look okay now). - Rare but serious: Heart attack, stroke, or problems with anesthesia. - Your smoking history and family background increase some risks, so quitting and good control of blood sugar/pressure help a lot. - If issues arise, they’re treated quickly—most people do well.   **6. Recovery After Surgery (Postoperative Care)**  **In the Hospital (First 5-7 Days)**   - You’ll wake up in intensive care with tubes for breathing, draining fluid, and monitoring. - Pain medicine will help—tell nurses if it hurts. - Start moving soon: sitting up, then walking with help to build strength. - Breathing exercises to keep lungs clear. - Manage diabetes with checks and insulin if needed. - Eat light foods when ready.   **At Home (First Few Weeks)**   - Rest a lot, but walk daily to improve circulation. - Care for your chest wound: Keep it clean and dry; watch for redness or fever (call doctor if seen). - No heavy lifting or driving for 4-6 weeks. - Take medicines as prescribed for pain, blood thinning, blood pressure, diabetes, and cholesterol. - Follow-up visits: See your doctor 1-2 weeks after discharge for checks.   **Long-Term Recovery (Months Ahead)**   - Cardiac rehab: Join a program with guided exercise, diet tips, and support—it’s great for your heart strength (currently at 55%). - Watch for signs like chest pain, swelling, or fever—call emergency if severe.   **7. Lifestyle Tips for a Healthier Future**  To keep your new bypasses working well and manage your conditions:   - **Diet**: Low-fat, low-sugar meals. Control portions for diabetes. - **Exercise**: Aim for 30 minutes of walking most days, building up slowly. - **Medicines**: Take them daily; don’t skip. - **Check-ups**: Regular tests for blood pressure, sugar, and cholesterol. - **Quit bad habits**: No smoking or heavy drinking. - **Stress management**: Hobbies, family time, or meditation. - **Weight and health goals**: Work toward better control of your conditions to live longer and feel better.   Thank you for reading this guide, Mr. Wang. Surgery can be a big step, but it often leads to a better quality of life. Write down questions and discuss them with your doctor. Wishing you a smooth surgery and quick recovery!  If you need more details or changes to this guide, let me know. Stay well! |
| 2.Case information: Patients scheduled for total knee arthroplasty (TKA) |
| Answer:  **Preoperative Education Guide for Your Knee Replacement Surgery**  Dear Ms. Li M.,  Hello! I’m here to help you understand everything about your upcoming right knee replacement surgery, also called total knee arthroplasty (TKA). As a medical professional, my goal is to make this information easy to read and understand. We’ll use simple words and avoid complicated medical terms. This guide is tailored to your situation: you’re a 72-year-old retired teacher with a history of high blood pressure, weak bones (osteoporosis), and an allergy to penicillin. You’ve had pain and trouble walking in your right knee for over 10 years, and treatments like pills, therapy, and injections haven’t helped enough. The surgery is planned to replace your damaged knee joint with an artificial one to reduce pain and improve movement.  This guide covers what to expect before, during, and after surgery. It’s important to follow these steps to make your surgery as safe and successful as possible. If you have questions, please ask your doctor or nurse. Let’s get started!  **Why You Need This Surgery**  Your right knee has severe wear and tear from osteoarthritis, which has caused pain, a bowed leg (varus deformity), and limited movement. This makes walking, sleeping, and daily activities hard. The surgery will remove the damaged parts of your knee and replace them with metal and plastic parts that act like a new joint. This should help you walk better without as much pain. Based on your X-rays and exams, your knee is at a very advanced stage, so this is a good option to improve your quality of life.  Remember, you have high blood pressure and osteoporosis, which we’ll manage carefully. Your allergy to penicillin means we’ll avoid that medicine and use safe alternatives if needed.  **Preparing for Surgery (What to Do Before Your Operation)**  Getting ready helps reduce risks and makes recovery smoother. Start these steps as soon as possible.  **Health Check-Ups and Tests**   - You’ve already had blood tests, urine tests, heart checks (ECG), chest X-ray, and knee X-rays. These show everything is stable, like your blood pressure (130/80 mmHg) and no major heart or lung issues. - If needed, we may do more checks closer to surgery to ensure your high blood pressure is under control and your bones are as strong as possible. - Tell your doctor about all medicines you’re taking, including your blood pressure pills, calcium tablets, and vitamin D for osteoporosis. You might need to stop or adjust some before surgery.   **Medicines and Allergies**   - Continue your blood pressure medicine unless told otherwise. Well-controlled blood pressure (like yours) lowers risks during surgery. - For osteoporosis, keep taking calcium and vitamin D as usual, but discuss any changes with your doctor. - Avoid penicillin due to your allergy (which causes rashes). We’ll use other antibiotics if needed to prevent infection. - Stop any blood-thinning medicines (like aspirin) at least 7-10 days before surgery if your doctor says so, to reduce bleeding risks. - Don’t take new over-the-counter medicines, herbs, or supplements without asking.   **Lifestyle Changes**   - **Stop Smoking and Limit Alcohol**: If you smoke, try to quit at least 2 weeks before surgery—it helps healing. Limit alcohol to avoid complications. - **Eat Healthy**: Focus on balanced meals with fruits, vegetables, proteins (like fish or eggs), and calcium-rich foods (like milk or yogurt) to support your bones and overall health. - **Exercise Gently**: Do light activities like walking with your cane to keep your muscles strong, but avoid anything that hurts your knee. - **Weight Management**: If you’re overweight, losing a little weight can help your new knee last longer, but don’t start a strict diet now—talk to your doctor. - **Home Setup**: Arrange your home for after surgery. Put things you need within easy reach, remove trip hazards like rugs, and consider a raised toilet seat or shower chair. Since you’re married, ask your spouse or family for help.   **The Day Before Surgery**   - Fast (no food or drink) after midnight, or as instructed, to keep your stomach empty for anesthesia. - Shower with antibacterial soap to clean your skin and lower infection risk. - Pack comfortable clothes, your cane, glasses, hearing aids if needed, and a list of your medicines. - Get a good night’s sleep.   If you feel unwell (like a fever or cold), call your doctor right away—surgery might need to be delayed.  **What to Expect on the Day of Surgery**  Your surgery is scheduled after your admission on July 15, 2025. Here’s a simple overview:   - **Arrival**: Come to the hospital early. You’ll change into a gown and meet the team (surgeon, nurses, anesthesiologist). - **Anesthesia**: You’ll get medicine to make you sleep or numb the area (spinal or general anesthesia). This is safe, and we’ll monitor your blood pressure and heart closely. - **The Procedure**: It usually takes 1-2 hours. The surgeon will make a cut on your knee, remove damaged bone and cartilage, and put in the new joint parts. They’ll fix your bowed leg to make it straighter. - **After Surgery**: You’ll wake up in a recovery room. We might use a tube to drain fluid from the knee temporarily. You’ll start pain medicine right away.   You won’t feel pain during surgery, and we’ll make sure you’re comfortable.  **Possible Risks and Complications**  All surgeries have some risks, but we take steps to minimize them, especially with your age and health history. We’ll watch for:   - Infection: Rare, but we’ll give antibiotics (not penicillin) and keep everything clean. - Blood Clots: Your legs might be still after surgery, so we’ll use stockings, pumps, or medicine to prevent clots. - Bleeding: Your blood tests are normal, so this is low risk. - Pain or Stiffness: Common at first, but improves with therapy. - Issues from High Blood Pressure or Osteoporosis: We’ll control your blood pressure and protect your bones during surgery. - Allergy Reactions: Avoided by not using penicillin. - Rare Problems: Like the new joint loosening over time (many last 15-20 years) or nerve issues.   If something doesn’t feel right after surgery, tell us immediately. Most people do well, especially with good preparation.  **After Surgery: Care and Recovery**  Recovery takes time, but following these steps will help.  **In the Hospital (First 3-5 Days)**   - **Pain Control**: You’ll get medicines (pills or IV) to manage pain. It’s normal to feel sore, but tell us if it’s too much. - **Moving Around**: Start walking with help the day after surgery using a walker or crutches. This prevents clots and builds strength. Your quadriceps (thigh) muscle is a bit weak now, so we’ll work on that. - **Wound Care**: Keep the bandage clean and dry. Nurses will change it and check for redness or swelling. - **Breathing and Eating**: Do deep breaths to keep lungs clear. Eat light foods when ready. - **Managing Your Conditions**: Continue blood pressure checks and osteoporosis meds.   **At Home (First Few Weeks)**   - **Rest and Ice**: Elevate your leg and use ice packs to reduce swelling. - **Exercises**: A physical therapist will teach simple moves to bend and straighten your knee. Do them daily to reach better movement (aim for 0-120° flexion eventually). - **Medicines**: Take pain relievers, blood thinners (if prescribed), and your usual pills. Watch for side effects like stomach upset. - **Daily Life**: Avoid driving until cleared (usually 4-6 weeks). Use your cane or walker. No heavy lifting. - **Diet**: Eat foods high in protein, vitamins, and calcium for healing and bone health. Stay hydrated. - **Signs to Watch For**: Call your doctor if you have fever, increasing pain, swelling, redness, or trouble breathing.   **Long-Term Recovery (Months to a Year)**   - Full recovery can take 3-6 months, but you’ll notice improvements weekly. - Attend follow-up visits for X-rays and checks. - Gradually return to activities: Walking without help in 4-6 weeks, stairs in 1-2 months. Avoid high-impact sports. - For your osteoporosis, keep up with bone health routines to protect your new knee. - Most people walk without pain and have better mobility than before.   **Emotional Support and Questions**  Surgery can be stressful, especially at 72. It’s okay to feel anxious—talk to family, friends, or a counselor. You’re in good spirits now, which is great!  Remember:   - Follow all instructions for the best results. - Contact your doctor anytime with concerns. - Emergency? Go to the hospital or call for help.   We’re here to support you through this. With your positive attitude and our care, you’ll be back to enjoying life soon. Wishing you a smooth surgery and quick recovery!  If you need this in another format or more details, let us know.  Best regards, Your Healthcare Team |
| 3.Case information: Patients scheduled for laparoscopic cholecystectomy (LC) |
| Answer:  **Preoperative Education Guide for Your Gallbladder Removal Surgery**  Hello, Ms. Zhang. My name is Dr. [Your Name], and I’m here to help you understand everything about your upcoming surgery. You’re scheduled for a laparoscopic cholecystectomy, which is a common and safe way to remove your gallbladder. This guide is written in simple language to make it easy to read and understand. We’ll cover what your condition means, why surgery is recommended, how to prepare, what happens during and after the procedure, and tips for recovery. If you have any questions, please ask your doctor or nurse.  This information is based on your health details: You’re a 45-year-old woman with no known allergies, no history of other major illnesses like high blood pressure or diabetes, and no previous surgeries. Your mother had gallbladder stones, which can sometimes run in families. Your recent checks show gallbladder stones causing inflammation (acute cholecystitis), leading to pain, nausea, and vomiting, especially after fatty foods.  Remember, this is general advice tailored to your case. Follow your doctor’s specific instructions, as they know your situation best.  **1. Understanding Your Condition**  Your gallbladder is a small organ under your liver that stores bile, a fluid that helps digest fats. You have stones in your gallbladder, which are like hard pebbles. These stones can block the flow of bile, causing sharp pain in the upper right side of your belly, nausea, and vomiting. This has been happening to you for about 2 years, and it got worse recently.  The stones have also caused some swelling and irritation in the gallbladder (called acute cholecystitis). Without treatment, this could lead to more pain or infection. Removing the gallbladder stops these problems. Most people live normally without a gallbladder because the liver takes over making bile.  **2. Why This Surgery and Its Benefits**  Surgery is recommended because your symptoms are getting worse, and tests show the stones and swelling. The goal is to remove the gallbladder to prevent future attacks, pain, and complications like infection or blockage in nearby areas.  **Benefits:**   - Relief from pain, nausea, and vomiting. - Lower risk of future health issues from the stones. - Quick recovery for most people – you can often go back to work as an accountant in a few weeks. - This is a minimally invasive surgery (done through small cuts), so there’s less pain and scarring than traditional open surgery.   **Possible Risks:** Like any surgery, there are small risks. These include:   - Infection at the surgery site (treated with medicine). - Bleeding (rare and usually minor). - Reaction to the sleep medicine (anesthesia) – but your checks show you’re healthy, so this is low risk. - Rarely, injury to nearby organs like the bile tubes or intestines. - Some people feel temporary shoulder pain from the gas used in surgery. - Since you have no allergies, the risk of medicine reactions is low.   Your doctor will explain these in more detail and answer questions. The benefits usually outweigh the risks for someone like you with ongoing symptoms.  **3. Preparing for Surgery**  Your surgery is planned after your admission on July 14, 2025. Here’s how to get ready:  **A. At Home Before Admission**   - **Diet:** For a few days before surgery, eat light, non-greasy foods to avoid triggering pain. Avoid fatty meals, as they worsen your symptoms. - **Medications:** Tell your doctor about any pills, vitamins, or herbs you take. You may need to stop some (like blood thinners) a few days before. Since you have no known medicines, this might not apply, but confirm. - **Lifestyle:** Stop smoking if you do (it helps healing). Limit alcohol. Stay active but avoid heavy lifting if it causes pain. - **Fasting:** You’ll be told not to eat or drink after midnight the night before surgery. This is important for safety. - **Arrangements:** Plan for someone to drive you home after discharge (usually 1-2 days after surgery). As a married accountant living in central China, arrange help with home tasks or work for the first week. - **Personal Items:** Pack comfortable clothes, toiletries, and any needed items like glasses. Leave valuables at home.   **B. Tests and Checks**  You’ve already had blood tests, urine tests, ultrasound, ECG, and chest X-ray, which look good. No major issues like high white blood cells or liver problems. If needed, more tests will be done before surgery.  **C. Emotional Preparation**  It’s normal to feel worried. Talk to family or friends. Relaxation techniques like deep breathing can help. Since you’re cooperative and in good spirits, that’s a great start.  **4. What to Expect on the Day of Surgery**   - **Arrival:** You’ll check in early. Nurses will check your vital signs (like temperature 37.0°C, pulse 85, breathing 18, blood pressure 125/75 – all normal for you). - **Preparation:** You’ll change into a hospital gown. An IV line (small tube in your arm) will give fluids and medicines. You’ll meet the anesthesia team for sleep medicine (likely general anesthesia, where you sleep fully). - **The Procedure:** It takes about 1-2 hours. The surgeon makes 3-4 small cuts in your belly. They use a tiny camera and tools to remove the gallbladder. Gas is used to inflate your belly for better view (this goes away after). Since your ultrasound shows enlarged gallbladder with stones but no major complications, it should be straightforward. - **In the Operating Room:** You’ll be asleep and won’t feel anything. The team monitors you closely.   **5. Right After Surgery**   - **Recovery Room:** You’ll wake up here, monitored for a few hours. You might feel groggy, have a sore throat from the breathing tube, or mild pain in your belly or shoulder. - **Pain Control:** Medicine will help with discomfort. Your abdomen is soft with tenderness, so expect some soreness, but it fades quickly. - **Hospital Stay:** Most people stay 1-2 days. You’ll start with clear liquids, then light food. Walk soon after to prevent clots. - **What You Might Feel:** Nausea (from anesthesia), but it passes. No eating right away to let your body adjust.   **6. Recovery at Home**  You’ll likely go home when you’re eating, walking, and pain is managed.   - **First Few Days:** Rest a lot. Mild pain is normal – use prescribed pain relievers. Avoid driving until off strong meds. - **Wound Care:** Keep small cuts clean and dry. Watch for redness, swelling, or pus (signs of infection). - **Diet:** Start with bland, low-fat foods. Over time, eat normally, but limit greasy foods to avoid loose stools (common after gallbladder removal). - **Activity:** No heavy lifting for 4-6 weeks. Walk daily to build strength. As an accountant, you can do light desk work soon, but take breaks. - **Follow-Up:** See your doctor in 1-2 weeks for a check. Report any fever over 38°C, severe pain, yellow skin, or vomiting. - **Full Recovery:** Most feel back to normal in 2-4 weeks. Since you’re healthy with no other issues, you should recover well.   **7. When to Call Your Doctor**  Contact your doctor or go to the emergency if:   - Fever, chills, or worsening pain. - Red, swollen, or draining wounds. - Persistent nausea, vomiting, or yellow skin/eyes. - Trouble breathing or chest pain. - Any new symptoms that worry you.   **8. Lifestyle Tips for Long-Term Health**   - Eat a balanced diet with fruits, veggies, and lean proteins. Watch portion sizes to maintain healthy weight. - Stay active – aim for 30 minutes of walking most days. - Since your mother had this, encourage family to watch for symptoms. - Regular check-ups keep you healthy.   This surgery should greatly improve your quality of life by ending the pain and discomfort. You’re in good hands with a skilled team. If anything is unclear, or you need this in another format, let us know. Wishing you a smooth surgery and quick recovery! |
| 4.Case information: Patients scheduled for transcatheter arterial chemoembolization (TACE) |
| Answer:  **Preoperative Education Guide for Your TACE Procedure**  Dear Mr. Ma M.,  Hello! My name is [Your Name or AI Assistant as Medical Educator], and I’m here to help you prepare for your upcoming Transcatheter Arterial Chemoembolization (TACE) procedure. This guide is designed to explain everything in simple, clear language so you can feel informed and ready. We’ll cover what the procedure is, why you’re having it, how to prepare, what happens on the day, possible risks, recovery tips, and more. If anything is unclear or you have questions, please talk to your doctor or nurse right away.  This information is based on your health details: You’re a 62-year-old man with liver cancer (hepatocellular carcinoma), liver scarring from hepatitis B (cirrhosis), and ongoing hepatitis B. You’ve had symptoms like belly pain, tiredness, and weight loss. Your tests show the cancer is in one part of your liver, and your overall health is stable enough for this treatment (Child-Pugh grade A). You have no known allergies, no history of diabetes or high blood pressure, and you’ve quit smoking, which is great!  Remember, TACE is not a full open surgery—it’s a less invasive way to treat your liver cancer using a thin tube inserted through a blood vessel. The goal is to help control the cancer and ease your symptoms. Let’s go through this step by step.  **1. Understanding Your Condition and Why TACE Is Recommended**   - **Your Condition**: You have liver cancer that started from long-term hepatitis B and liver scarring. The cancer is in a specific area of your liver (about 5 cm in size) and hasn’t spread to other major parts. You’ve lost some weight and feel tired with belly pain, but your other tests (like blood work, heart, and lungs) look okay. - **What Is TACE?**: TACE is a treatment where doctors use a small tube (catheter) to deliver medicine and tiny particles directly to the cancer in your liver. This blocks the blood supply to the tumor, helping to shrink it or slow its growth. It’s like targeting the problem area without affecting the rest of your body as much. - **Why This Treatment?**: Based on your tests, TACE is a good option because your liver function is still strong, and the cancer is at a stage where this can help (BCLC Stage B). It may improve your symptoms like pain and tiredness. It’s often done instead of surgery if the cancer can’t be fully removed right away.   **2. How to Prepare Before the Procedure**  Getting ready helps everything go smoothly and reduces risks. Start these steps as soon as possible, and follow your doctor’s specific advice.   - **Medications and Supplements**:   - Keep taking your regular medicines unless your doctor says to stop. Since you have hepatitis B, you might need antiviral pills—ask if you should start or continue them.   - Avoid blood-thinning medicines (like aspirin) for a few days before, if told. Don’t take any new over-the-counter pills, herbs, or vitamins without checking first. - **Eating and Drinking**:   - You may need to stop eating and drinking (fast) for 6-8 hours before the procedure. Usually, no food after midnight if it’s scheduled for the morning.   - If you have any stomach issues, eat light meals in the days leading up. - **Lifestyle Changes**:   - Stay smoke-free—great job quitting 2 years ago! Avoid alcohol completely, as it can harm your liver more.   - Rest well and eat nutritious foods to build strength. Focus on easy-to-digest meals like rice, vegetables, lean meats, and fruits to help with your appetite and weight loss.   - If you’re feeling very tired, ask family for help with daily tasks. - **Tests and Check-Ups**:   - You’ve already had blood tests, scans, and a biopsy. You might need one more blood check or ECG close to the procedure day.   - Tell your doctor about any new symptoms, like fever, more pain, or yellow skin/eyes. - **Practical Preparations**:   - Arrange a ride home—TACE is often outpatient (you go home the same day or after 1-2 days), but you can’t drive yourself.   - Pack comfortable clothes, ID, insurance info, and any needed medicines. Bring a family member for support.   - If you live far (like in western China), plan for travel and possible overnight stay. - **Emotional Preparation**:   - It’s normal to feel worried. Talk to your family or a counselor. Knowing your father had liver cancer might bring up feelings—share this with your care team.   **3. What to Expect on the Day of the Procedure**  TACE usually takes 1-2 hours and is done in a special room with X-ray guidance. You’ll be awake but relaxed with medicine.   - **Arrival and Check-In**:   - Arrive early (e.g., 1-2 hours before). You’ll change into a gown and have an IV line placed for fluids and medicines.   - Your vital signs (like blood pressure, 128/78 mmHg last time) will be checked. - **During the Procedure**:   - You’ll lie on a table. The doctor numbs the skin in your groin or arm and inserts a thin tube into a blood vessel.   - Using X-rays, they guide the tube to your liver, inject cancer-fighting medicine and blocking particles into the tumor’s blood supply.   - You might feel some warmth or mild pain, but pain relief medicine will help. It’s not like open surgery—no big cuts. - **Right After**:   - You’ll rest in a recovery area for a few hours. Nurses will watch for any issues like bleeding or pain.   - You might feel groggy from the relaxing medicine.   **4. Possible Risks and Side Effects**  Like any procedure, there are risks, but your team will watch closely. Your good liver function (Child-Pugh A) lowers some risks.   - **Common Side Effects**: Belly pain, fever, nausea, or tiredness for a few days (called post-embolization syndrome). These usually go away with rest and medicine. - **Less Common Risks**: Infection, bleeding at the tube site, liver irritation, or allergic reaction to the medicine (but you have no known allergies). - **Rare but Serious**: Damage to healthy liver parts, blood clots, or worsening hepatitis B— that’s why monitoring is key. - **What Reduces Risks**: Your stable health (normal blood counts, no edema, etc.) helps. Follow all prep instructions.   If you notice severe pain, high fever, yellow skin, vomiting blood, or swelling, call your doctor immediately.  **5. Recovery and Aftercare**  Recovery is usually quick, but your liver needs time to heal.   - **Right After Going Home**:   - Rest for 1-2 days. Avoid heavy lifting or driving for a week.   - Drink plenty of fluids and eat small, healthy meals to help with nausea and appetite.   - Take pain medicine as prescribed. Keep the tube site clean and dry. - **Longer-Term Care**:   - Follow-up visits: You’ll have scans and blood tests (like AFP levels, which were high at 2500 ng/mL) to check how well it worked.   - Manage hepatitis B: Stick to antiviral treatment to protect your liver.   - Lifestyle: Eat well, stay active gently (like walking), and avoid smoking/alcohol. Aim to regain your 5 kg weight loss slowly.   - Watch for signs: Report any new pain, fatigue, or swelling. - **When to Get Help**:   - Emergency: Severe chest pain, trouble breathing, heavy bleeding, or confusion—go to the hospital.   - Non-emergency: Mild fever or questions—call your doctor.   **6. Healthy Living Tips After TACE**   - **Diet**: Choose foods that are kind to your liver, like veggies, fruits, whole grains, and low-fat proteins. Avoid fatty or spicy foods if they upset your stomach. - **Activity**: Start with light walks to fight tiredness. Build up slowly. - **Support**: Lean on your family (you’re married, so involve your spouse). Join a support group for liver cancer patients. - **Follow-Up Treatments**: TACE might be repeated, or other options discussed based on results.   **7. Questions to Ask Your Doctor**   - How many times might I need TACE? - What if I feel worse after? - Can I take my usual stomach medicines? - How will this affect my daily life as a retired worker?   You’re taking a positive step for your health, Mr. Ma. This procedure can help manage your cancer and improve how you feel. If you need more details or changes to this guide, just let me know. Stay strong!  Best regards, [Your Name or AI Assistant as Medical Educator] Date: [Current Date]  *Note: This is general education based on your info. Always follow your doctor’s personalized advice.* |
| 5.Case information: Patients scheduled for appendectomy |
| Answer:  **Preoperative Education Guide for Your Appendectomy Surgery**  Hello, Mr. Chen. My name is [Your Name or AI Assistant acting as a Medical Professional], and I’m here to help you understand everything about your upcoming surgery for appendicitis. This guide is written in simple, everyday language to make it easy to read and understand. We’ll cover what appendicitis is, why you need surgery, how to prepare, what happens during and after the procedure, possible risks, and tips for recovery. If you have any questions or need something explained differently, please ask your doctor or nurse.  This information is based on your specific situation: You’re a 23-year-old university student with sudden right lower belly pain that started about 12 hours ago. Tests show you have acute appendicitis, which means your appendix is inflamed and needs to be removed to prevent it from getting worse. You have no known allergies, no past health issues, and you’re otherwise healthy. Surgery is planned soon to help you feel better quickly.  **1. Understanding Your Condition: What is Appendicitis?**   - Your appendix is a small, finger-shaped pouch attached to the start of your large intestine in the lower right side of your belly. - Appendicitis happens when this pouch gets blocked or infected, causing swelling, pain, and sometimes fever. In your case, the pain started in the upper belly and moved to the lower right side, with nausea and tenderness when touched. - If not treated, it can burst and cause a serious infection in your belly. That’s why surgery to remove the appendix (called an appendectomy) is the best way to fix it. This is a common surgery, especially for young adults like you, and most people recover fully without problems.   **2. Why Surgery and What Type?**   - Surgery is needed to remove the inflamed appendix and stop the pain and infection from spreading. - Based on your tests (like blood work showing high white blood cells and an ultrasound confirming the swelling), this is the right step. - The surgery is usually done with small cuts (keyhole surgery or laparoscopic appendectomy), which means quicker recovery. If needed, it might be done with a larger cut (open surgery), but your doctor will decide based on how things look inside. - The goal is to get you back to your studies and normal life as soon as possible.   **3. Preparing for Surgery: What You Need to Do Beforehand**  Getting ready helps make the surgery safer and smoother. Here’s a step-by-step guide:   - **Fasting (No Food or Drink):** You won’t be able to eat or drink anything for at least 6-8 hours before surgery. This is to keep your stomach empty and reduce risks during anesthesia (the medicine that makes you sleep). Your doctor will tell you the exact time to stop. - **Medications:** Tell your team about any medicines, vitamins, or supplements you’re taking. Since you have no allergies or chronic conditions, this should be straightforward. You might need to stop certain things, but your doctor will guide you. If you feel pain, they can give you safe relief before surgery. - **Hygiene and Personal Items:** Take a shower with mild soap the night before or morning of surgery. Remove any jewelry, nail polish, or makeup. Wear comfortable clothes and bring a small bag with essentials like ID, insurance info, and glasses if you need them. Leave valuables at home. - **Tests and Checks:** You’ve already had blood tests, urine tests, and an ultrasound, which look good except for signs of infection. You might have a quick check of your heart and lungs before surgery. - **Lifestyle Prep:** Since you don’t smoke or drink, that’s great—it helps with healing. Get a good night’s rest if possible, and arrange for a family member or friend to help you get home after (you can’t drive yourself). - **Mental Preparation:** It’s normal to feel nervous. Talk to your doctor about any worries. Breathing exercises or listening to calm music can help.   **4. What Happens on the Day of Surgery?**   - You’ll arrive at the hospital (you were admitted on July 19, 2025) and change into a hospital gown. - A nurse will check your vital signs (like temperature, heart rate, and blood pressure—yours are stable with a slight fever at 37.8°C). - You’ll meet the anesthesia team, who will explain how they’ll make you comfortable and asleep during the surgery. This is usually through a vein in your arm. - In the operating room, the surgery takes about 30-60 minutes. The surgeon removes the appendix through small cuts, cleans the area, and closes everything up. - You’ll wake up in a recovery area where nurses monitor you closely.   **5. After Surgery: What to Expect in the Hospital and at Home**   - **Right After:** You might feel groggy, have a sore throat from the breathing tube, or mild pain in your belly or shoulders (from the gas used in keyhole surgery). Pain medicine will help. You’ll start with clear liquids and move to soft foods as your belly settles. - **Hospital Stay:** For a simple case like yours, you might go home the same day or after 1-2 days. Nurses will check for any issues like fever or infection. - **Recovery at Home:**   - **Pain Management:** Expect some soreness for a few days. Use prescribed pain relievers, and apply ice packs if needed.   - **Activity:** Rest for the first few days, but walk short distances to prevent blood clots. Avoid heavy lifting or strenuous activity (like sports) for 1-2 weeks. As a student, take it easy with studying—give yourself time to heal.   - **Wound Care:** Keep the small cuts clean and dry. Change dressings as instructed. Watch for redness, swelling, or pus—call your doctor if you see this.   - **Diet:** Start with light, easy-to-digest foods like soup, rice, and fruits. Avoid greasy or spicy foods at first. Drink plenty of water to help with bowel movements, as pain meds can cause constipation.   - **Follow-Up:** You’ll have a check-up in about 1-2 weeks to ensure everything is healing well. - **Full Recovery Time:** Most people like you are back to normal in 1-3 weeks. You can return to university classes gradually, but listen to your body.   **6. Possible Risks and Complications**  All surgeries have some risks, but appendectomy is very safe, especially for a healthy young person like you. We’ll watch closely to minimize them:   - Common: Mild pain, bruising, or constipation. - Less Common: Infection at the cut sites, bleeding, or reaction to anesthesia (nausea or dizziness). - Rare: The appendix bursting before removal, or issues like a leak in the intestine. If something feels off (like worsening pain, high fever over 38.5°C, vomiting, or swelling), contact your doctor right away—don’t wait. - Your good health (no smoking, normal blood pressure) lowers these risks.   **7. Lifestyle Tips for a Smooth Recovery and Future Health**   - **Prevent Future Issues:** Eat a balanced diet with fiber to keep your bowels healthy. Stay active to maintain your weight and energy. - **When to Seek Help:** Call your doctor if you have severe pain, fever, redness around wounds, or can’t keep food down. In emergencies, go to the hospital. - **Emotional Support:** Surgery can be stressful. Talk to friends, family, or a counselor if needed. As a student, consider light activities like reading to stay positive. - **Long-Term:** Removing the appendix doesn’t affect your daily life—you won’t miss it!   Remember, this guide is general and based on your details, but your doctor will give personalized advice. Follow their instructions closely. You’re in good hands, and we expect a quick recovery. If you need more details or have questions, let us know. Wishing you a smooth surgery and fast healing! |
| 6.Case information: Patients scheduled for transurethral resection of the prostate (TURP) |
| Answer:  **Preoperative Education Guide for Your Prostate Surgery**  Hello, Mr. Meng. My name is [Your Name or AI Assistant], and I’m here to help you understand everything about your upcoming surgery. You’re scheduled for a procedure called transurethral resection of the prostate (TURP). This is a common surgery to help with your prostate problem, which is causing trouble with urination. I’ll explain everything in simple, easy-to-understand words. We’ll cover what to expect before, during, and after the surgery, how to prepare, and tips for a smooth recovery. If you have any questions, please ask your doctor or nurse.  This guide is based on your health details: You’re 75 years old, have had high blood pressure for 10 years (which is well controlled with medicine), and no allergies or other major health issues. Your prostate is enlarged (called benign prostatic hyperplasia or BPH), leading to urinary retention, and that’s why this surgery is planned. The goal is to remove part of the prostate to make urination easier and relieve your symptoms like difficulty starting to urinate, weak stream, and frequent nighttime trips to the bathroom.  Let’s break it down step by step.  **1. Understanding Your Condition and Why Surgery Is Needed**   - **Your prostate issue**: The prostate is a small gland near your bladder that helps with making semen. In many older men, it grows larger over time (this is BPH, not cancer). This growth can squeeze the tube that carries urine from your bladder, making it hard to urinate. You’ve had this for 5 years, and it recently got worse with complete blockage (urinary retention), causing pain and swelling in your bladder. - **Why TURP?**: This surgery removes the extra prostate tissue that’s blocking your urine flow. It’s done through a small tube inserted into the penis—no big cuts on your belly. It should help you urinate normally again and reduce nighttime bathroom trips. Based on your tests (like ultrasound and blood work), your prostate is enlarged but looks normal otherwise, and your overall health is good for this procedure.   **2. Preparing for Surgery (What to Do Before the Big Day)**  Getting ready helps make the surgery safer and your recovery easier. Start these steps as soon as possible, and follow your doctor’s specific instructions.   - **At home before admission**:   - **Medications**: Keep taking your blood pressure medicine as usual unless your doctor says to stop. Tell your doctor about any other pills, vitamins, or herbal supplements you’re using. If you take blood thinners (you don’t, based on your history), they might need adjusting.   - **Diet and lifestyle**: Eat a balanced diet with plenty of fruits, vegetables, and fiber to avoid constipation. Stay active with light walks if possible, but avoid heavy lifting. Quit smoking if you smoke—it helps healing. Limit alcohol.   - **Fasting**: Don’t eat or drink anything after midnight the night before surgery. This includes water, gum, or candy. If your surgery is later in the day, your doctor might allow clear liquids earlier—ask them.   - **Hygiene**: Take a shower the night before or morning of surgery with regular soap. Don’t shave the surgery area yourself.   - **Arrange help**: Plan for someone (like a family member) to drive you home after discharge and help at home for a few days. Since you’re married and live in a city, make sure your spouse or family knows your schedule. - **What to bring to the hospital**:   - Comfortable clothes, slippers, toiletries, glasses, and any hearing aids.   - Your ID, insurance info, and a list of your medicines.   - Leave valuables at home. - **Tests and checks**: You’ve already had blood tests, urine tests, heart checks (ECG), chest X-ray, and ultrasound—all look good. Your kidney function is slightly off but should improve after surgery. If needed, you might have one more check before surgery. - **Special notes for you**: Since you have high blood pressure, your team will monitor it closely. You’ve had a catheter (tube to drain urine) placed— it might stay in until after surgery to keep your bladder empty.   Stop any activities that could cause injury, like heavy exercise, a few days before.  **3. What to Expect on the Day of Surgery**   - **Arrival**: You’ll check in at the hospital (your admission is July 18, 2025). A nurse will review your info, check your vital signs (like temperature: yours was 36.7°C, pulse: 70, breathing: 18, blood pressure: 135/85), and you might change into a hospital gown. - **Pre-surgery area**: You’ll meet the anesthesia team and surgeon. They might start an IV (a small tube in your arm for fluids and medicines). You could get medicine to help you relax. - **Family**: Your family can wait with you until it’s time to go to the operating room.   **4. About the Surgery and Anesthesia**   - **How the surgery works**: You’ll be in the operating room for about 1-2 hours. The surgeon uses a thin, lighted tube (called a resectoscope) inserted through your penis into the urethra (urine tube). They remove the overgrown prostate tissue using a special tool. No outside cuts are made—it’s all internal. You’ll have a catheter in place afterward to drain urine and help healing. - **Anesthesia**: This keeps you comfortable and pain-free. For TURP, it’s usually spinal anesthesia (a shot in your back to numb from the waist down—you stay awake but feel nothing) or general anesthesia (you sleep through it). Your team will choose based on your health; since your heart and lungs are fine, either should be okay. They’ll explain risks like temporary numbness or headache from spinal anesthesia.   **5. Possible Risks and Side Effects**  All surgeries have some risks, but TURP is generally safe, especially with your good health. We’ll watch for these closely:   - Common: Temporary bleeding in urine (it usually clears up), infection (prevented with antibiotics), or irritation when urinating. - Less common: Issues with erection or controlling urine (these often improve over time), or fluid imbalance from the procedure. - Rare: Heart strain (but your blood pressure is controlled), allergic reactions (you have no known allergies), or need for more surgery. - Your specific risks: At 75, healing might take a bit longer, but your tests show no major concerns like kidney problems or heart issues.   If anything unusual happens, your team is prepared. The benefits (easier urination, less pain) usually outweigh the risks.  **6. After Surgery: Recovery and Care**   - **Right after**: You’ll go to a recovery area for monitoring (1-2 hours). You might feel groggy if you had general anesthesia. Pain is usually mild—managed with pills or shots. - **Hospital stay**: Often 1-3 days. Your catheter stays in for a few days to a week to let the area heal. Urine might look pink or have clots at first—that’s normal. - **Pain management**: Expect some discomfort in your lower area or bladder spasms. Take pain meds as prescribed (not on an empty stomach). Use ice packs if suggested. - **What to watch for**: Call a nurse if you have severe pain, fever over 38°C, heavy bleeding, or trouble breathing. - **Going home**: Once you can eat, walk, and urinate okay (after catheter removal), you’ll be discharged. Have someone with you the first day.   **7. At-Home Recovery Tips**   - **First few weeks**: Rest a lot, but walk short distances to prevent blood clots. Avoid straining, lifting heavy things (>5 kg), or driving for 1-2 weeks. - **Diet**: Drink plenty of water (8-10 glasses/day) to flush your system. Eat soft foods if needed; avoid constipation with fruits and stool softeners. - **Catheter care (if you go home with one)**: Keep it clean, empty the bag regularly, and watch for signs of infection (fever, cloudy urine). - **Activity**: No sex or strenuous exercise for 4-6 weeks. Resume light work when you feel ready, but check with your doctor. - **Medicines**: Continue blood pressure meds. You might get antibiotics or bladder relaxants. - **Follow-up**: See your doctor 1-2 weeks after for a check-up. They’ll monitor your PSA (prostate marker) and urination. - **Lifestyle changes**: Maintain a healthy weight, eat well, and manage blood pressure to prevent future issues. If symptoms return (like weak stream), tell your doctor.   **8. When to Call Your Doctor or Seek Emergency Help**  Contact your doctor right away if:   - Heavy bleeding (soaking a pad hourly). - Fever, chills, or bad-smelling urine (possible infection). - Severe pain not helped by meds. - Swelling in legs, chest pain, or shortness of breath. - Can’t urinate after catheter removal.   Go to the emergency room if you have sudden severe symptoms.  **Final Thoughts**  This surgery should greatly improve your quality of life by easing your urination problems. You’re in good hands with your medical team. Remember, everyone’s recovery is different—be patient and follow instructions. Share this guide with your family so they can support you.  If anything is unclear or you need more details, talk to your doctor. Wishing you a smooth surgery and quick recovery! |
| Baidu-Ernie-4.5-Turbo |
| 1.Case information: Patients scheduled for coronary artery bypass grafting (CABG) |
| Answer:  **Preoperative Education Material for Coronary Artery Bypass Grafting (CABG)** **Patient Name**: Wang M. **Gender**: Male **Age**: 68 years old **Scheduled Surgery Date**: [Insert Date]  **1. Understanding Your Surgery (CABG)**  **Why is this surgery needed?** Your heart’s blood vessels (coronary arteries) are severely narrowed or blocked due to plaque buildup (atherosclerosis). This reduces blood flow to your heart, causing chest pain (angina) and increasing your risk of a heart attack. CABG will create new pathways for blood to reach your heart by using healthy blood vessels from other parts of your body (e.g., chest, leg, or arm).  **How is the surgery performed?**   1. **Anesthesia**: You will be given medicine to sleep deeply (general anesthesia) so you won’t feel pain. 2. **Incision**: The surgeon will make a cut down the middle of your chest to access your heart. 3. **Bypass Grafting**: Healthy blood vessels will be attached to bypass the blocked areas of your coronary arteries. 4. **Closing Up**: The chest bone will be closed with wires (like sturdy stitches), and the skin will be closed with stitches or staples. 5. **Recovery**: You’ll wake up in a recovery room with tubes to drain fluid and monitors to track your heart and breathing.   **How long will it take?** The surgery usually takes **3–6 hours**, but plan to spend **5–7 days** in the hospital for recovery.  **2. Preparing for Surgery**  **Tests and Screenings**: You’ve already completed most tests, but additional ones may include:   - **Chest X-ray**: To check your lungs and heart size. - **Carotid Ultrasound**: To assess blood flow to your brain (reduces stroke risk). - **Lung Function Tests**: To ensure your lungs are healthy enough for surgery.   **Medications**:   - **Stop taking**:   - **Blood thinners** (e.g., aspirin, warfarin) **7 days before surgery** (they increase bleeding risk).   - **Diabetes pills** (e.g., metformin) on the morning of surgery (ask your doctor). - **Continue taking**:   - **Blood pressure pills** (e.g., ACE inhibitors) with a small sip of water on surgery day.   - **Insulin**: Your dose may be adjusted; follow your doctor’s instructions. - **New medications**: You may receive antibiotics or heart medications before surgery.   **Diet and Fasting**:   - **Do not eat or drink anything** (including water, gum, or mints) after **midnight** the night before surgery. - If you take morning medications, ask your doctor if you can take them with a tiny sip of water.   **Lifestyle Adjustments**:   - **Quit smoking**: Even though you quit 1 year ago, avoid secondhand smoke to improve lung healing. - **Avoid alcohol**: Stop drinking **48 hours before surgery** to reduce bleeding risks. - **Stay active**: Light walking is okay, but avoid heavy lifting or strenuous exercise.   **Other Preparations**:   - Arrange for a family member to drive you home and stay with you for **at least 3 days** after surgery. - Pack a bag with loose-fitting clothes, slippers, and personal items (e.g., toothbrush, glasses, hearing aids). - Leave valuables (e.g., jewelry, watches) at home.   **3. What to Expect on Surgery Day**  **Arrival**:   - Arrive at the hospital **2 hours before your scheduled surgery time**. - Check in at the admissions desk and proceed to the preoperative area.   **Pre-Op Procedures**:   - A nurse will review your medical history, check your vital signs, and place an IV line in your arm for fluids and medications. - You’ll change into a hospital gown and meet your anesthesia team to discuss pain control options. - You may receive a sedative to help you relax before surgery.   **During Surgery**:   - The surgical team will monitor your heart rate, blood pressure, and oxygen levels closely. - Your family will wait in a designated area and will be updated after surgery.   **4. After Surgery: Immediate Recovery**  **Recovery Room (PACU)**:   - You’ll wake up in a recovery room where nurses will monitor you for **1–2 hours**. - You may have:   - A breathing tube (removed once you wake up).   - Tubes in your chest to drain fluid.   - An IV line for fluids and medications.   - A catheter (tube in your bladder) to drain urine (removed in 1–2 days).   **Pain Management**:   - You’ll receive pain medication through your IV or as pills. Tell your nurse if the pain is severe. - Ice packs or a cooling blanket may help reduce swelling.   **Breathing and Circulation**:   - **Deep breathing exercises**: Take slow, deep breaths every hour to prevent lung infections. - **Coughing**: Use a pillow to support your chest while coughing to ease pain. - **Leg movements**: Flex and point your feet or use compression stockings to improve blood flow and prevent blood clots.   **Eating and Drinking**:   - Start with sips of water or ice chips. If you don’t feel nauseous, you’ll progress to clear liquids (broth, juice) and then soft foods.   **5. Going Home: Post-Op Care**  **Incision Care**:   - Keep the bandage clean and dry. Your nurse will show you how to change it or when to shower. - Watch for signs of infection: **redness, swelling, pus, or fever**. Contact your doctor if these occur. - Avoid soaking in baths or pools until your doctor says it’s safe.   **Activity Restrictions**:   - **No driving** for **4–6 weeks** (check with your doctor first). - **No lifting** anything heavier than **10 pounds** for **6 weeks**. - **No strenuous exercise** (e.g., jogging, heavy lifting) for **3 months**. - Gradually increase activity (e.g., short walks) as tolerated.   **Follow-Up Appointments**:   - Schedule a post-op visit with your surgeon in **1–2 weeks** to check healing progress. - Attend cardiac rehabilitation (a supervised exercise and education program) to improve heart health.   **When to Call the Doctor**: Contact your surgeon or go to the ER if you experience:   - **Severe chest pain** not relieved by medication. - **Fever above 101°F (38.3°C)**. - **Heavy bleeding** or drainage from the incision. - **Shortness of breath** or **chest tightness**. - **Swelling or redness** in your legs (possible blood clot).   **6. Managing Chronic Conditions After Surgery**  **High Blood Pressure (Hypertension)**:   - Take your blood pressure pills as prescribed. - Check your blood pressure at home regularly (your doctor will show you how).   **Diabetes**:   - Monitor your blood sugar **4 times a day** (before meals and at bedtime). - Follow a heart-healthy diet (low in sugar, salt, and saturated fats).   **High Cholesterol (Hyperlipidemia)**:   - Avoid fatty foods (e.g., fried foods, red meat). - Take cholesterol medication (e.g., statins) as prescribed.   **7. Emotional Support and Resources**  It’s normal to feel anxious before surgery. Talk to your doctor, nurse, or a counselor if you need support. Consider:   - Bringing a family member or friend for emotional support. - Using relaxation techniques (deep breathing, meditation) to stay calm. - Joining a cardiac support group (ask your nurse for local resources).   **Contact Information**:   - **Hospital Pre-Op Team**: [Phone Number] - **Surgeon’s Office**: [Phone Number] - **Cardiac Rehabilitation Center**: [Phone Number] - **Emergency Contact**: [Name and Phone Number]   **Final Reminder**: This guide covers the basics, but your care team may give you additional instructions. **Don’t hesitate to ask questions!** Your safety and comfort are our top priorities.  **We wish you a smooth surgery and a strong recovery!**  *This material is for educational purposes only. Always follow your healthcare provider’s specific instructions.* |
| 2.Case information: Patients scheduled for total knee arthroplasty (TKA) |
| Answer:  **Preoperative Education Material for Total Knee Arthroplasty (TKA)** **Patient Name**: Li M. **Gender**: Female **Age**: 72 years old **Surgery Date**: [Insert Date] **Surgical Procedure**: Total Knee Arthroplasty (Right Knee Replacement)  **1. Welcome & Introduction**  Dear Mrs. Li, This guide is designed to help you prepare for your upcoming knee replacement surgery. Our team is committed to ensuring you feel informed, comfortable, and supported throughout this process. Please read this carefully and ask questions anytime—we’re here to help!  **2. Why Is This Surgery Needed?**  Your surgery will replace the damaged parts of your right knee joint with artificial components to:   - **Relieve severe pain** caused by osteoarthritis (Grade IV, the most advanced stage). - **Correct the "O-shaped leg" (varus deformity)** to improve balance and walking. - **Restore mobility** so you can walk, climb stairs, and perform daily activities without pain or a cane.   **3. Preparing Your Body for Surgery**  **A. Medical Tests & Clearances**   - You’ve already completed blood tests, an EKG, and a chest X-ray—all results were normal, which is great! - **Hypertension & Osteoporosis**: Your blood pressure is well-controlled with medication, and your bone density is stable with calcium/vitamin D supplements. Continue these as prescribed.   **B. Diet & Fasting**   - **The night before surgery**: Do not eat or drink anything after midnight (including water, gum, or mints). This prevents complications during anesthesia. - **Morning of surgery**: If instructed, take your blood pressure medication with a small sip of water.   **C. Smoking & Alcohol**   - Avoid smoking and alcohol for at least 24 hours before surgery to promote healing.   **D. Skin Preparation**   - Shower the morning of surgery using mild soap. Do not shave the knee area—we’ll handle this to prevent infections.   **4. Managing Medications**   - **Allergies**: You’re allergic to penicillin (rash). We’ll use alternative antibiotics during/after surgery. - **Continue taking**: Blood pressure medications and calcium/vitamin D unless told otherwise. - **Stop taking**: Herbal supplements (e.g., ginseng, garlic) 1 week before surgery, as they may affect bleeding. - **Pain relief**: Avoid NSAIDs (e.g., ibuprofen) 3 days before surgery unless approved by your doctor.   **5. What to Wear & Bring**   - **Wear**: Loose pants (e.g., sweatpants) and flat shoes. Avoid jewelry, makeup, or nail polish. - **Bring**:   - Insurance card and ID.   - A list of all medications (including doses).   - Glasses/hearing aids (if needed).   - A small bag for personal items (leave valuables at home).   - A book or phone charger for waiting time.   **6. The Day of Surgery**  **A. Arrival & Check-In**   - Arrive **2 hours before** your scheduled surgery time. Check in at the front desk and proceed to the preoperative area.   **B. Before the Operating Room**   - You’ll change into a hospital gown and meet your anesthesia team. - An IV will be placed in your arm to deliver fluids and medications. - Your surgeon will review the plan and answer any last-minute questions.   **C. Anesthesia**   - You’ll receive **spinal anesthesia** (numbs the lower body) combined with sedation (you’ll sleep through surgery). This reduces risks compared to general anesthesia.   **D. During Surgery**   - Surgery will take **1.5–2 hours**. Your family can wait in the designated area and will be updated when surgery is complete.   **7. After Surgery: Recovery Room**   - You’ll wake up in a recovery area with nurses monitoring your vital signs. - Expect some grogginess, nausea, or soreness at the IV site—this is normal. - Pain medication will be given through your IV or as a pill.   **8. Going Home: Discharge Instructions**  **A. Activity Restrictions**   - Avoid bending your knee past 90°, lifting >10 pounds, or driving for **4–6 weeks**. - Use a walker or cane as instructed by physical therapy. - Sleep with a pillow under your knee to keep it straight.   **B. Wound Care**   - Keep the surgical site clean and dry. Your nurse will show you how to change dressings if needed. - Watch for signs of infection: redness, swelling, or yellow drainage. Report these immediately.   **C. Pain Management**   - Take prescribed pain medications (e.g., acetaminophen, opioids) as directed. Do not wait until pain is severe. - Use ice packs (20 minutes on, 20 minutes off) to reduce swelling.   **D. Diet & Hydration**   - Start with clear liquids (broth, juice) and progress to soft foods (e.g., mashed potatoes, scrambled eggs). - Stay hydrated unless instructed otherwise.   **E. Follow-Up Appointments**   - **First post-op visit**: Schedule with your surgeon in **10–14 days** to check the incision and remove stitches. - **Physical therapy**: Begin within 1–2 days after surgery to regain strength and mobility.   **9. When to Call the Doctor**  Contact us immediately if you experience:   - Fever >101°F (38.3°C). - Severe pain not relieved by medication. - Shortness of breath or chest pain. - Redness/swelling at the surgical site. - Difficulty urinating or moving your bowels for >2 days.   **10. Support & Resources**   - **Pre-Op Class**: Attend our free joint replacement class on [date/time] to learn exercises and tips. - **Contact Information**:   - Surgeon’s Office: [Phone Number]   - Hospital Pre-Op Team: [Phone Number]   - After-Hours Emergency Line: [Phone Number]   **11. Final Checklist**   - Confirm transportation to/from the hospital. - Arrange help at home for the first 2 weeks (e.g., cooking, cleaning). - Pack your bag with essentials (medications, loose clothing, toiletries). - Fast as instructed (no food/drink after midnight). - Review this guide and write down questions for your doctor.   **We’re Here for You!** This surgery is a big step toward a more active, pain-free life. Trust our team to guide you every step of the way. Thank you for choosing us—we’re honored to care for you.  Warm regards, **[Your Name/Title]** **[Hospital/Clinic Name]** **[Contact Information]**  *This material is for educational purposes only. Always follow your doctor’s specific instructions.* |
| 3.Case information: Patients scheduled for laparoscopic cholecystectomy (LC) |
| Answer:  **Preoperative Education Guide for Ms. Zhang M.** *Preparing for Your Laparoscopic Cholecystectomy (LC)*  **1. Understanding Your Surgery**  **What is laparoscopic cholecystectomy (LC)?** This surgery removes your gallbladder using small incisions and a camera (laparoscope). It treats **gallbladder stones** and **acute cholecystitis** (inflammation of the gallbladder), which have caused your severe right upper abdominal pain, nausea, and vomiting.  **Why is surgery needed?** Your gallbladder has multiple stones and is inflamed, causing recurrent pain. Removing it will relieve symptoms and prevent future complications like infections or bile duct blockages.  **How long will it take?** The surgery lasts **1–2 hours**, followed by a short recovery period in the hospital.  **What are the risks?** Risks are low but include infection, bleeding, injury to nearby organs (e.g., bile duct), or anesthesia reactions. Your surgeon will explain these in detail.  **2. Preparing Your Body for Surgery**  **Medical Tests**: You’ve already completed blood tests, an EKG, chest X-ray, and abdominal ultrasound. These confirm you’re healthy enough for surgery.  **Medications**:   - **Stop** blood thinners (e.g., aspirin) or herbal supplements (e.g., ginseng) **7 days before surgery** unless your doctor says otherwise. - **Continue** daily medications (e.g., for blood pressure) with a small sip of water on surgery morning, unless told to stop. - **Bring a list** of all medications and allergies to your preoperative appointment.   **Diet Restrictions**:   - **Do not eat or drink anything** (including water, gum, or mints) after **midnight the night before surgery**. - **If you take morning medications**, ask your doctor if you can take them with a tiny sip of water.   **Smoking and Alcohol**:   - **Avoid smoking** and **alcohol** for **24 hours before surgery** to reduce healing risks.   **Skin Preparation**:   - **Shower or bathe** the night before or morning of surgery using **antibacterial soap** (provided by the hospital) to lower infection risk. - **Do not shave** the surgical area; hospital staff will handle this.   **3. What to Bring to the Hospital**   - **ID and insurance card**. - **List of medications and allergies**. - **Comfortable, loose-fitting clothes** (e.g., pajamas, button-up shirt). - **Personal items**: Glasses, hearing aids, or dentures (leave jewelry and valuables at home). - **A family member or friend** to drive you home and stay with you afterward.   **Do NOT bring**:   - Jewelry, piercings, or contact lenses. - Large sums of money or credit cards.   **4. Day of Surgery: What to Expect**  **Arrival Time**: Arrive **2 hours before surgery** to check in, change into a hospital gown, and meet your surgical team.  **Anesthesia**: You’ll receive general anesthesia (sleep medicine) through an IV. An anesthesiologist will monitor you throughout surgery.  **During Surgery**:   - You’ll be asleep and pain-free. - The surgeon will make 3–4 small incisions in your abdomen, insert a camera and tools, and remove your gallbladder.   **After Surgery**:   - You’ll wake up in a recovery room with nurses checking your vitals. - You may feel groggy, nauseous, or have mild shoulder pain (from gas used during surgery). This is temporary.   **5. Recovery at Home**  **Pain Management**:   - Take pain medications as prescribed. Use ice packs on your abdomen (20 minutes on, 20 minutes off) to reduce swelling.   **Wound Care**:   - Keep incisions clean and dry. Your doctor will tell you when to shower or remove bandages. - Watch for signs of infection: redness, swelling, pus, or fever >100.4°F (38°C). Call your doctor if these occur.   **Activity Restrictions**:   - **Avoid lifting >10 pounds**, strenuous exercise, or driving for **1 week** (or until your doctor says it’s safe). - Walk short distances daily to prevent blood clots.   **Diet**:   - Start with **clear liquids** (broth, juice) and progress to soft foods (mashed potatoes, toast) as tolerated. - Avoid **greasy/spicy foods** for 4–6 weeks to prevent digestive upset.   **Follow-Up Appointments**:   - Return **7–10 days after surgery** for a wound check. - Ask your doctor when you can resume work (usually 1–2 weeks for desk jobs, longer for physical labor).   **6. When to Call the Doctor**  Contact your surgeon or go to the ER if you experience:   - Fever >100.4°F (38°C). - Severe pain not relieved by medication. - Redness, swelling, or drainage from incisions. - Persistent nausea/vomiting or inability to eat/drink. - Yellowing of skin/eyes (jaundice) or dark urine.   **7. Support System**   - Arrange for a family member/friend to help with meals, chores, and childcare (if needed) for **3–5 days**. - Keep emergency contacts (doctor, hospital) handy.   **8. Questions to Ask Your Surgeon**   - How long until I can return to work/exercise? - When can I shower or bathe? - Will I need a special diet long-term? - What are the signs of a bile leak (rare but serious)?   **Final Reminder**: Your health is our priority. If you have concerns, call **[Hospital Name] at [Phone Number]** or visit **[Website/Contact Page]**. Rest well before surgery, and follow all instructions carefully!  *Prepared by: [Your Name/Title, e.g., "Dr. Li, Surgeon"]* *Date: July 10, 2025*  **Note**: This guide supplements, but does not replace, discussions with your healthcare team. Always follow your doctor’s specific advice. |
| 4.Case information: Patients scheduled for transcatheter arterial chemoembolization (TACE) |
| Answer:  **Preoperative Education Guide for Transcatheter Arterial Chemoembolization (TACE) for Mr. Ma M.** *Preparing for Your Procedure*  **1. Understanding TACE: What Is It and Why Is It Needed?**  **What is TACE?** TACE is a minimally invasive treatment for liver cancer. A thin tube (catheter) is inserted through a small cut in your groin or wrist and guided to the blood vessels supplying the tumor in your liver. Special drugs (chemotherapy) and tiny particles are injected to block blood flow to the tumor, shrinking it and controlling its growth.  **Why is TACE recommended for you?**   - You have been diagnosed with **hepatocellular carcinoma (liver cancer)** in the S7 segment of your liver, along with **liver cirrhosis** due to hepatitis B. - TACE is a good option because it targets the tumor directly while minimizing damage to healthy liver tissue, especially since your liver function is still stable (Child-Pugh grade A). - The goal is to **reduce tumor size, relieve symptoms (like abdominal pain), and improve your quality of life**.   **2. Preoperative Preparations**  **Medical Tests and Clearances**   - You’ve already completed key tests: blood work, liver/kidney function, CT scan, and liver biopsy. These confirm you’re ready for TACE. - An **electrocardiogram (ECG)** and **chest X-ray** ensure your heart and lungs are healthy enough for the procedure.   **Medications**   - **Continue taking**:   - Antiviral medications for hepatitis B (if prescribed).   - Blood pressure or other regular medications **with a small sip of water** on the morning of TACE. - **Stop taking**:   - Blood thinners (e.g., aspirin, warfarin) or herbal supplements (e.g., ginseng, garlic pills) **3–5 days before TACE** unless your doctor says otherwise.   - Pain relievers like ibuprofen or naproxen, as they can increase bleeding risk.   **Fasting Instructions**   - **Do not eat or drink anything (including water)** after **midnight the night before TACE**. - If your procedure is scheduled later in the day, your doctor may allow a light breakfast early in the morning—follow their specific instructions.   **Smoking and Alcohol**   - Avoid smoking or drinking alcohol **at least 24 hours before TACE**. Smoking can slow healing, and alcohol can stress your liver.   **3. Day of TACE: What to Expect**  **Arrival Time**   - Arrive **2–3 hours before your scheduled TACE time** to check in, change into a hospital gown, and meet with your care team.   **What to Wear/Bring**   - Wear loose, comfortable clothing. - Leave jewelry, watches, and valuables at home. - Bring a list of all medications you take and any necessary personal items (e.g., glasses, hearing aids).   **During the Procedure**   1. You’ll lie on an X-ray table. 2. The doctor will numb a small area in your groin or wrist and insert a catheter (thin tube) into a blood vessel. 3. Using X-ray guidance, the catheter is moved to the liver’s blood vessels supplying the tumor. 4. Chemotherapy drugs and tiny particles are injected to block blood flow and shrink the tumor. 5. The procedure takes **1–2 hours**, followed by a short recovery period.   **Anesthesia**   - You’ll receive **local anesthesia** (numbing medicine) at the catheter insertion site and **mild sedation** to help you relax. You’ll be awake but drowsy and won’t feel pain.   **4. After TACE: Immediate Recovery**  **Recovery Room**   - You’ll wake up in a recovery area with nurses monitoring your blood pressure, heart rate, and the insertion site for bleeding. - You may feel groggy or nauseous from the sedation—this is normal and passes quickly.   **Pain Management**   - You may have mild to moderate pain or a burning sensation at the catheter site or in your abdomen. - Pain medication (e.g., acetaminophen) will be given as needed. Tell your nurse if pain isn’t controlled.   **Activity Restrictions**   - **First 6–12 hours**: Lie flat with your leg or arm straight (if the catheter was inserted in your groin/wrist) to prevent bleeding. - **First 24 hours**: Avoid driving, operating machinery, or making important decisions (sedation effects). - **Next few days**: Rest at home, avoid heavy lifting (>10 pounds), and gradually increase activity as tolerated.   **5. Post-TACE Care at Home**  **Incision Care**   - Keep the insertion site clean and dry for **24–48 hours**. - Avoid showering for **24 hours**; use a sponge bath instead. After 24 hours, you may shower but gently pat the site dry. - Watch for signs of infection: redness, swelling, warmth, or drainage. Report these to your doctor immediately.   **Diet and Hydration**   - Start with clear liquids (broth, juice, water) after TACE, then advance to soft foods (toast, rice) as tolerated. - Avoid fatty, spicy, or fried foods for a few days if they cause nausea. - Drink plenty of water to stay hydrated and help your liver recover.   **Fatigue and Appetite**   - You may feel tired for **1–2 weeks** after TACE. Rest when needed and gradually resume light activities. - Eat small, frequent meals if your appetite is poor. Focus on protein-rich foods (eggs, yogurt, lean meat) to maintain strength.   **Side Effects to Expect**   - **Fever (up to 100.4°F/38°C)**: Common for 1–2 days as your body reacts to the treated tumor. Drink fluids and take acetaminophen if needed. - **Nausea/vomiting**: May occur due to anesthesia or chemotherapy drugs. Your doctor can prescribe anti-nausea medication. - **Abdominal pain**: Mild to moderate cramping is normal as the tumor breaks down. Pain medication will help. - **Fatigue**: Can last up to 2 weeks. Pace yourself and ask for help with daily tasks.   **When to Call the Doctor**   - Fever above **101°F (38.3°C)** or chills. - Severe pain not relieved by medication. - Heavy bleeding, swelling, or redness at the catheter site. - Vomiting blood or dark, coffee-ground-like material. - Yellowing of the skin/eyes (jaundice) or dark urine.   **6. Follow-Up Care**  **First Appointment**   - Schedule a follow-up visit **1–2 weeks after TACE** to check your recovery and discuss the next steps (e.g., additional treatments or scans).   **Imaging Tests**   - A **CT scan or MRI** will be done **4–6 weeks after TACE** to see how well the tumor has responded.   **Long-Term Management**   - TACE may be repeated if the tumor grows back or new lesions appear. - Continue regular check-ups with your liver specialist (hepatologist) to monitor your liver health and cancer status.   **7. Support and Resources**  **Contact Information**   - **Hospital phone**: [Insert number] - **Oncology nurse coordinator**: [Insert name/phone] - **24/7 emergency line**: [Insert number]   **Emotional Support**   - Talk to your family, friends, or a counselor about your feelings. - Consider joining a **liver cancer support group** (ask your care team for recommendations).   **Financial/Practical Help**   - If needed, ask about social work services for transportation, financial assistance, or home care support.   **8. Frequently Asked Questions**  **Q: Will TACE cure my liver cancer?** A: TACE is not a cure but can shrink tumors, relieve symptoms, and extend survival. Your doctor will discuss long-term treatment plans with you.  **Q: How long will I stay in the hospital?** A: Most patients go home the **same day** or stay **1 night** for observation.  **Q: Can I eat before TACE?** A: No—follow fasting instructions strictly to avoid complications from anesthesia.  **Q: When can I return to normal activities?** A: Light activities (walking, cooking) can resume in **3–5 days**. Avoid strenuous exercise or heavy lifting for **1–2 weeks**.  **Q: Will I lose my hair from chemotherapy?** A: No—the chemotherapy in TACE is delivered directly to the liver, so hair loss is rare.  **9. Final Checklist Before TACE**   - Confirm transportation to/from the hospital. - Arrange for a family member/friend to stay with you for **24 hours** after discharge. - Pack a bag with comfortable clothes, toiletries, and entertainment (books, phone charger). - Follow all fasting and medication instructions carefully.   **We’re Here to Help!** If you have questions or concerns, please call **[Hospital Phone]** or ask your nurse/doctor during your next visit. Our goal is to ensure you feel informed and supported throughout your treatment.  *Best wishes for a successful TACE and smooth recovery!*  **Note**: This guide is tailored to your specific case. Always follow your doctor’s instructions, as individual needs may vary. |
| 5.Case information: Patients scheduled for appendectomy |
| Answer:  **Preoperative Education Material for Mr. Chen M. (Scheduled for Appendectomy)** *Simple, clear, and tailored to your needs.*  **1. Welcome and Introduction**  Hello, Mr. Chen! You’re scheduled for surgery to treat acute appendicitis, a condition where your appendix (a small pouch in the abdomen) has become inflamed and needs to be removed. This guide explains everything you need to know to feel prepared and confident. If you have questions, please ask your doctors or nurses—we’re here to help!  **2. Understanding Your Appendectomy**   - **Why is surgery needed?** Your appendix is infected and could burst if not removed quickly. Surgery prevents complications like severe infection or abscesses. - **How is the surgery done?**   - **Anesthesia**: You’ll receive general anesthesia (asleep and pain-free) for the entire procedure.   - **Incision**: The surgeon will make 1–2 small cuts (about 1–2 inches) in your lower abdomen to remove the appendix.   - **Duration**: The surgery will take **30 minutes to 1 hour**.   **3. Preparing for Surgery**  **A. Tests and Screenings**   - You’ve already had blood tests, an ultrasound, and a physical exam to confirm appendicitis. No additional tests are needed.   **B. Medications**   - **Continue taking**: Any regular medications (e.g., for allergies) unless your doctor says to stop. - **Stop taking**:   - Aspirin, ibuprofen, or other pain relievers **7 days before surgery** (they can increase bleeding risk).   - Herbal supplements (e.g., ginseng, garlic pills) **3 days before surgery**.   **C. Eating and Drinking**   - **Do not eat or drink anything after midnight** the night before surgery (including water, gum, or mints). - **Morning medications**: If approved, take with a tiny sip of water.   **D. Smoking and Alcohol**   - Avoid smoking or using tobacco **24 hours before surgery**. - Avoid alcohol **48 hours before surgery**.   **E. Personal Preparation**   - **Bathing**: Shower the night before or morning of surgery using antibacterial soap (if provided). - **Clothing**: Wear loose, comfortable clothes. Leave jewelry, watches, and valuables at home. - **Nails**: Remove nail polish and acrylic nails.   **4. What to Bring to the Hospital**   - Photo ID and insurance card. - A list of all medications you take (including doses). - Comfort items (e.g., headphones, a book, or a pillow). - **Do not bring**: Valuables like jewelry or large sums of cash.   **5. The Day of Surgery**   - **Arrival time**: Plan to arrive **2 hours before your scheduled surgery** (e.g., if surgery is at 8:00 AM, arrive by 6:00 AM). - **Check-in**: You’ll register and meet a nurse to review your health history. - **Anesthesia**: You’ll speak with an anesthesia provider to discuss how you’ll stay asleep and pain-free. - **IV line**: A small needle will be placed in your arm to give fluids and medications. - **Operating room**: You’ll move to the OR on a stretcher. The team will ensure you’re comfortable before starting.   **6. During Surgery**   - You’ll be monitored the entire time by a specialized team. - Your family/friends can wait in the designated area and will be updated when surgery is done.   **7. After Surgery (Recovery Room)**   - You’ll wake up in a recovery area where nurses will check your vital signs (e.g., heart rate, breathing). - You may feel groggy, nauseous, or have a sore throat (from the breathing tube). - Pain medication will be given as needed.   **8. Going Home (Discharge)**   - **Same-day discharge**: Most appendectomies are outpatient (you’ll go home the same day). - **Criteria for going home**:   - You can eat, drink, and use the bathroom.   - Your pain is controlled with medication.   - You can walk safely (with help if needed).   **9. Caring for Yourself at Home**  **A. Wound Care**   - Keep the incision clean and dry. Your doctor will tell you when you can shower (usually after **24–48 hours**). - Watch for signs of infection: redness, swelling, or yellow/green drainage. Report these to your doctor.   **B. Pain Management**   - Take pain medications (e.g., acetaminophen/Tylenol) as prescribed. Don’t wait until pain is severe. - Use an ice pack (wrapped in a towel) on your abdomen for **10–15 minutes at a time** to reduce swelling.   **C. Activity**   - **Rest often**, but walk a little each day to prevent blood clots. - **Avoid**:   - Heavy lifting (>10 pounds) for **1–2 weeks**.   - Strenuous exercise (e.g., running, weightlifting) for **2–4 weeks**.   - Driving until you’re no longer taking pain medication and feel alert (usually **1–2 weeks**).   **D. Diet**   - Start with clear liquids (e.g., broth, apple juice), then advance to soft foods (e.g., toast, rice) as tolerated. - Avoid spicy, greasy, or hard-to-digest foods for **3–5 days**. - Stay hydrated! Drink plenty of water.   **E. Follow-Up Appointments**   - Schedule a post-op visit with your doctor **7–10 days after surgery** to check your healing.   **10. When to Call the Doctor**  Contact your healthcare team if you experience:   - Fever above **100.4°F (38°C)**. - Severe pain not relieved by medication. - Redness, warmth, or pus at the incision site. - Vomiting or inability to keep fluids down for **more than 12 hours**. - Difficulty urinating or passing gas/stool.   **11. Support and Resources**   - **Family/friends**: Ask them to help with errands, cooking, or schoolwork for a few days. - **University**: Let your professors know you’ll need time to recover. Many schools offer medical leave options. - **Online support**: Search for “appendectomy recovery tips” from reputable sites like Mayo Clinic or WebMD.   **12. Final Checklist Before Surgery**   - Confirm transportation to/from the hospital. - Arrange help at home for **3–5 days** after surgery. - Pack your bag with essentials (ID, medications, comfort items). - Follow medication, eating, and drinking instructions carefully.   **You’re in Good Hands!** Appendicitis surgery is common and has a high success rate. By following these instructions, you’ll be back to your university routine in no time!  **Contact Information**   - Hospital phone number: [Insert] - After-hours emergency line: [Insert] - Your doctor’s office: [Insert]   *Wishing you a smooth surgery and speedy recovery, Mr. Chen! 🌸* |
| 6.Case information: Patients scheduled for transurethral resection of the prostate (TURP) |
| Answer:  **Preoperative Education Material for Mr. Meng M.** **Preparing for Your Transurethral Resection of the Prostate (TURP) Surgery**  **1. Welcome and Overview**  Dear Mr. Meng, This guide is designed to help you and your family prepare for your upcoming **TURP surgery**, a procedure to relieve symptoms caused by an enlarged prostate. We want you to feel informed and supported throughout this process. If you have questions, please contact our team at [Hospital Phone Number/Email].  **2. Understanding Your Surgery**  **What is TURP?** TURP stands for **Transurethral Resection of the Prostate**. During the procedure:   - A thin, lighted tube (resectoscope) is inserted through the urethra (the tube that carries urine from the bladder) to remove excess prostate tissue. - This opens the urethra, improving urine flow and relieving symptoms like difficulty urinating or frequent nighttime trips to the bathroom.   **Why is it needed?** You’ve been experiencing **benign prostatic hyperplasia (BPH)**, a non-cancerous enlargement of the prostate that blocks urine flow. Recently, you developed **acute urinary retention** (inability to urinate), which required emergency catheterization. TURP will prevent this from happening again and improve your quality of life.  **Procedure Details**   - **Duration**: About 60–90 minutes. - **Hospital Stay**: Typically 1–2 days. - **Anesthesia**: Spinal anesthesia (you’ll be awake but numb from the waist down) or general anesthesia (asleep), depending on your health and preference.   **3. Preoperative Preparations**  **Tests and Screenings** Before surgery, you’ll have:   - **Blood tests** (to check for anemia, clotting, or infection). - **Urine test** (to rule out infection). - **Electrocardiogram (ECG)** and **chest X-ray** (to assess heart and lung health). - **Urinary system ultrasound** (already completed; confirms prostate size and bladder function).   **Medications**   - **Continue taking** your blood pressure medications as usual (with a small sip of water on surgery day). - **Stop blood thinners** (e.g., aspirin, clopidogrel) **7 days before surgery** to reduce bleeding risk. - **Avoid herbal supplements** (e.g., ginseng, garlic) **1 week before surgery**. - **If you take diabetes medications**, your doctor will adjust them before surgery.   **Fasting Instructions**   - **Do not eat or drink anything after midnight** the night before surgery (including water, gum, or mints). - **Take approved medications** (e.g., blood pressure pills) with a tiny sip of water.   **Lifestyle Adjustments**   - **Quit smoking** (if applicable) to improve healing and reduce infection risk. - **Arrange for help** at home for **1–2 weeks** after surgery (e.g., cooking, cleaning, transportation). - **Pack a bag** with loose-fitting pants, non-slip shoes, and personal items (glasses, hearing aids). Leave valuables at home.   **4. Day of Surgery**  **What to Expect**   1. **Check-in**: Arrive at the hospital **2 hours before your scheduled surgery time**. 2. **Prep area**: You’ll change into a hospital gown and meet your surgical team. 3. **Anesthesia**: A specialist will discuss your options (spinal or general anesthesia). 4. **Surgery**: Your family can wait in the designated area and will be updated afterward.   **5. After Surgery: Immediate Recovery**  **In the Recovery Room**   - You’ll wake up in a room with monitors checking your heart rate, blood pressure, and oxygen levels. - A **catheter** (flexible tube) will remain in your bladder to drain urine. This is normal and will be removed in 1–2 days. - You may feel groggy or have a sore throat from the breathing tube (if general anesthesia was used).   **Hospital Stay**   - You’ll stay in the hospital for **1–2 days** for monitoring. - Nurses will help you walk shortly after surgery to prevent blood clots. - You’ll drink fluids to ensure the catheter is draining properly.   **6. Going Home: Postoperative Care**  **Catheter Care**   - If the catheter remains after discharge, your nurse will teach you how to care for it at home. - **Signs of infection**: Redness, swelling, or foul-smelling urine. Contact us if this occurs.   **Pain Management**   - You may have mild discomfort or burning during urination. Take prescribed pain medications as directed. - Use a warm compress (not hot) on your lower abdomen to ease bladder spasms.   **Activity and Rest**   - **Avoid heavy lifting (>10 pounds)** or strenuous activity for **4–6 weeks**. - **Walk daily** to improve circulation, but avoid long trips or driving until cleared by your doctor. - **No sexual activity** for **4 weeks** to allow healing.   **Diet**   - Start with clear liquids (broth, juice), then progress to soft foods (oatmeal, mashed potatoes). - Drink plenty of water (8–10 glasses/day) to prevent constipation and urinary infections. - Avoid alcohol, caffeine, and spicy foods for **2 weeks** (they can irritate the bladder).   **Bowel Movements**   - Constipation is common after surgery. Take stool softeners (e.g., docusate) as prescribed and eat high-fiber foods (prunes, vegetables).   **Follow-Up Appointments**   - Your first post-op visit is scheduled for **[date/time]** to check your healing and remove the catheter (if still in place). - Attend all appointments to monitor progress.   **7. Potential Risks and Complications**  While rare, risks include:   - **Bleeding**: May require a blood transfusion or another procedure. - **Infection**: Of the bladder, prostate, or surgical site. - **Urinary incontinence**: Temporary leakage of urine (usually improves with time). - **Retrograde ejaculation**: Semen flows backward into the bladder during orgasm (harmless but may affect fertility; not a concern if you’ve completed your family).   **When to Call the Doctor**   - Heavy bleeding (urine looks bright red or clotted). - Fever >100.4°F (38°C) or chills. - Severe pain not relieved by medication. - Inability to urinate after catheter removal.   **8. Emotional and Mental Preparation**   - **Anxiety is normal**. Practice deep breathing or listen to calming music before surgery. - **Lean on your spouse/family** for support. Let them know how they can help (e.g., accompanying you to appointments). - **Focus on recovery goals**: “I’ll sleep better without frequent nighttime trips to the bathroom” or “I’ll enjoy activities without urgency.”   **9. Resources and Support**   - **Contact Information**:   - Hospital: [Phone Number]   - Surgeon’s Office: [Phone Number/Email]   - After-Hours Nurse Line: [Phone Number] - **Support Services**:   - Physical therapy: [Contact Details] (if needed for mobility).   - Home health care: [Contact Details] (for catheter care or meal delivery).   **10. Final Checklist Before Surgery**   - Confirm transportation to/from the hospital. - Pack your bag with essentials (loose clothing, toiletries, medications). - Arrange post-op help at home (e.g., spouse, children, or hired caregiver). - Follow fasting and medication instructions carefully. - Write down questions for your surgical team (e.g., “When can I resume my morning walks?”).   **We’re Here to Help!** Your care team is committed to ensuring a smooth surgery and recovery. If you feel confused or overwhelmed, please reach out. Together, we’ll help you regain comfort and independence.  Warm regards, [Your Name/Title] [Hospital Name] [Contact Information]  **Note**: This material is tailored to Mr. Meng M.’s case (TURP for BPH with acute urinary retention). Adjustments can be made for language preferences or additional medical details. |
| Deepseek-R1 |
| 1.Case information: Patients scheduled for coronary artery bypass grafting (CABG) |
| Answer：  **Comprehensive Preoperative Education Guide for Coronary Artery Bypass Grafting (CABG)**  **1. Introduction** Hello, Mr. Wang! This guide is designed to prepare you for your upcoming heart surgery and answer common questions. Our goal is to ensure you feel informed, safe, and supported throughout the process.  **2. Understanding Your Surgery**   - **What Will Happen**: You will undergo **coronary artery bypass grafting (CABG)**. This surgery will create new pathways for blood to flow around the blocked parts of your heart arteries. The surgeon will use healthy blood vessels (usually from your leg, chest, or arm) to bypass the narrowed or blocked arteries in your heart. - **Purpose**: This surgery aims to **improve blood flow to your heart**, reduce chest pain, lower your risk of heart attack, and help you breathe more easily.   **3. Preparing for Surgery**  **Medical Preparations**:   - **Tests Before Surgery**:   - Blood tests, chest X-ray, and ECG (heart tracing) to ensure you’re ready.   - Your diabetes and blood pressure will be closely monitored. - **Medications**:   - **Continue**:     - Most blood pressure medications (unless your doctor specifies otherwise).     - Diabetes medications (your care team will adjust doses based on blood sugar levels).   - **Stop**:     - **Blood thinners** (e.g., aspirin, clopidogrel, ibuprofen) **7 days before surgery**. Your doctor will confirm the exact list.     - Avoid herbal supplements (e.g., fish oil, garlic pills) to reduce bleeding risks. - **Fasting**:   - **No food or drinks** (including water, gum, or candy) **after midnight the night before surgery**. This prevents complications during anesthesia.   **Practical Preparations**:   - **What to Bring**:   - ID, insurance card, glasses (no contact lenses), and a list of your current medications.   - Comfortable, loose-fitting clothing for after surgery. - **What to Wear**: Wear simple, button-up pajamas or a shirt. Avoid jewelry, watches, or belts. - **Arrangements**:   - Arrange for a **trusted adult to drive you home** and stay with you for **at least 1–2 weeks** after surgery.   - Prepare your home with easy-to-reach essentials (e.g., medications, water, phone).   **4. Day of Surgery**   - **Check-In**: Arrive at the hospital by **6:00 AM** on **July 10th, 2025**. A nurse will review your details, insert an IV (for fluids and medication), and prepare you for surgery. - **Pre-Op Care**:   - You will meet your surgical team, including your surgeon and anesthesiologist.   - You’ll receive general anesthesia to keep you **pain-free and asleep** during the surgery. - **Family Updates**: Your family can wait in the **surgery waiting area**. The team will update them during and after surgery.   **5. During Surgery**   - The surgery typically takes **3–6 hours**, depending on the number of bypasses needed. - Your surgeon will use blood vessels from your leg (saphenous vein) or chest (internal mammary artery) to create the bypasses.   **6. Recovery After Surgery**  **Immediate Recovery (Hospital Stay: 5–7 Days)**:   - **ICU Stay**: You’ll wake up in the intensive care unit (ICU) for **1–2 days** for close monitoring.   - Tubes and monitors will be attached to check your heart, drainage, and oxygen levels.   - Nurses will manage your pain with medication through an IV or pills. - **Wound Care**:   - Incisions on your chest and leg will be covered with bandages. Keep them clean and dry.   - Mild soreness, swelling, or bruising around the incisions is normal.   **At-Home Recovery (4–6 Weeks)**:   - **Activity**:   - Rest often but take short walks daily to improve circulation.   - Avoid lifting objects heavier than **5 pounds** (2 kg), driving, or strenuous activities. - **Diet**:   - Follow a **heart-healthy diet**: low salt, low sugar, and low fat. Focus on vegetables, lean proteins, and whole grains.   - Monitor your blood sugar closely (due to diabetes). - **Incision Care**:   - Wash incisions gently with mild soap and water. Pat dry; avoid scrubbing.   - Watch for signs of infection: redness, swelling, pus, or fever over **101°F (38.3°C)**. - **Warning Signs**: Contact your doctor immediately if you experience:   - Severe chest pain, shortness of breath, or dizziness.   - Sudden weight gain or swelling in your legs (signs of fluid retention).   **7. Follow-Up Care**   - **First Appointment**: Schedule a follow-up with your surgeon **1–2 weeks after discharge** to check your incisions and recovery progress. - **Cardiac Rehabilitation**: You’ll be enrolled in a **supervised exercise and education program** to strengthen your heart safely.   **8. Possible Risks**  Though rare, risks include:   - Infection, bleeding, or blood clots. - Irregular heart rhythms (temporarily managed with medication). - Reactions to anesthesia. Your care team will take steps to minimize these risks.   **9. Managing Chronic Conditions**   - **Blood Pressure**: Continue medications and monitor daily. Aim for **<140/90 mmHg**. - **Diabetes**: Check blood sugar regularly. Your doctor may adjust medications post-surgery. - **Cholesterol**: Start cholesterol-lowering medications (e.g., statins) to protect your heart.   **10. Emotional Support**   - Feeling anxious or low energy is common. Share concerns with your care team or family. - Practice deep breathing or meditation to relax. - Join a cardiac support group if you’d like to connect with others who’ve had similar surgeries.   **11. Contact Information**  For questions or urgent concerns:   - **Surgeon’s Office**: [Insert Phone Number] (Available 8:00 AM–5:00 PM). - **After-Hours Line**: [Insert Hospital Emergency Number]. - **Emergency**: Dial 911 or go to the nearest ER for chest pain, fainting, or severe shortness of breath.   **Conclusion** You’re in good hands, Mr. Wang! Follow these steps, attend all appointments, and prioritize rest. Your care team is here to ensure your surgery and recovery go smoothly.  Wishing you a strong recovery, [Your Hospital/Care Team Name]  *Note: Replace [bracketed] details with hospital-specific information.* |
| 2.Case information: Patients scheduled for total knee arthroplasty (TKA) |
| Answer:  Here’s the **personalized preoperative education guide** for Li M. based on her case details:  **Preoperative Education Guide for Total Knee Arthroplasty (TKA)**  **Patient Name:** Li M. **Age:** 72 **Surgery Date:** July 15, 2025  **1. Procedure Overview**   - **What You’re Having Done:** You will undergo **total knee replacement surgery (TKA)** on your right knee. This surgery will replace the damaged bone and cartilage with artificial parts to restore knee function. - **Why It’s Needed:** Your knee has severe arthritis (grade IV) with significant pain, deformity, and stiffness. This surgery aims to **eliminate pain, correct the O-shaped leg deformity, and improve mobility**, allowing you to walk and perform daily activities more comfortably. - **Duration:** The surgery will take about **1.5–2 hours**.   **2. Preoperative Preparation**  **A. Medications**   - **Stop Blood Thinners:**   - Avoid aspirin, ibuprofen, or other blood-thinning medications for **7 days before surgery**. Your doctor will confirm which medications to pause.   - **Continue Taking:**     - **Hypertension pills** (take with a small sip of water on the morning of surgery).     - **Osteoporosis medications** (calcium and vitamin D). - **Allergy Alert:** Inform every healthcare provider about your **penicillin allergy** (causes rash).   **B. Fasting**   - **No Food or Drink After Midnight** on July 14, 2025. - **Clear Liquids Allowed** (water, black tea, apple juice) until **2 hours before arriving at the hospital**.   **C. Lifestyle Adjustments**   - **Avoid Alcohol**: For at least **3 days before surgery** to reduce risks. - **Arrange Support**: Your spouse or a trusted adult **must drive you home** and stay with you for **at least 24 hours after surgery**.   **D. Pre-Surgery Tests (Already Completed):**   - Blood tests, ECG, chest X-ray, and knee X-rays. No further tests are needed.   **E. What to Bring/Wear**   - **Wear**: Loose pants or a skirt (easy to put on over bandages), slip-on shoes, and avoid jewelry/makeup. - **Bring**: ID, insurance card, walking cane, and a list of your medications.   **3. Day of Surgery**  **A. Arrival**   - Arrive at the hospital by **6:00 AM**. Check-in at the **Orthopedic Surgery Wing**.   **B. Anesthesia**   - You will receive **spinal anesthesia** (numbing medication injected near the spine) with sedation to keep you relaxed and pain-free during surgery. - **Rare Risks**: Nausea, headache, or temporary leg numbness.   **C. During Surgery**   - Your family can wait in the designated area. Staff will update them after surgery.   **4. After Surgery**  **A. Recovery Room**   - You’ll wake up with a bandaged knee. Nurses will monitor your pain, blood pressure, and leg movement. - **Common Side Effects**: Grogginess, mild nausea (medication will help).   **B. Pain Management**   - Pain relief medications will be given through an IV initially, then switched to pills. - **Avoid NSAIDs** (like ibuprofen) due to your osteoporosis; safer alternatives will be prescribed.   **C. Activity Restrictions**   - **First 6 Weeks**:   - Use a **walker or cane** for support. Avoid squatting, kneeling, or twisting your knee.   - **No driving** until your doctor approves.   - Perform gentle ankle exercises to prevent blood clots.   **D. Diet**   - Start with bland foods (rice porridge, broth) to avoid nausea. Resume your usual diet as tolerated.   **E. Wound Care**   - Keep the incision clean and dry. Bandages will be changed during your follow-up visits. - **No soaking** the knee in baths or pools until cleared by your doctor (about 3–4 weeks).   **F. Warning Signs (Call Your Doctor Immediately):**   - Fever > 101°F (38.3°C). - Severe redness, swelling, or pus around the incision. - Sudden calf pain or trouble breathing (possible blood clot).   **5. Follow-Up Plan**   - **First Post-Op Visit**: July 22, 2025 (1 week after surgery) to check the incision and remove stitches. - **Physical Therapy**: Starts 2–3 days after surgery to strengthen your knee and improve mobility. - **Bone Health**: Continue calcium/vitamin D and schedule a bone density test in 6 months.   **6. Questions or Concerns?**   - **Surgeon’s Team**: Dr. [Name] at [Phone Number]. - **Nurse Line**: [Hospital Contact] (24/7). - **Emergency**: Go to [Hospital Name] ER if severe symptoms arise.   **Important Notes for Li M.:**   - Your osteoporosis and hypertension are well-controlled—keep taking your medications as directed. - Use your cane until your physical therapist approves walking without it. - Practice deep breathing exercises after surgery to keep your lungs clear.   **We’re here to support your journey to a pain-free knee!**  This guide emphasizes simplicity while addressing Li M.’s specific needs (osteoporosis, hypertension, penicillin allergy). Let me know if further adjustments are needed! |
| 3.Case information: Patients scheduled for laparoscopic cholecystectomy (LC) |
| Answer:  **Preoperative Education Materials for Laparoscopic Cholecystectomy** **Patient Name:** Zhang M. \| **Age:** 45 \| **Scheduled Surgery:** Laparoscopic Gallbladder Removal \| **Date:** July 14, 2025  **1. Overview of Your Surgery**  **Purpose:** This surgery will remove your gallbladder to stop the pain caused by gallbladder stones and inflammation. Removing the gallbladder will prevent future episodes of pain, nausea, and vomiting, especially after eating greasy foods. **Procedure:**   - You will be given anesthesia (medication to make you sleep) so you feel no pain. - The surgeon will make 3–4 small cuts (incisions) in your abdomen. - A tiny camera and tools will be used to gently remove the gallbladder. - The incisions will be closed with stitches or glue and covered with small bandages.   **2. Preparing for Surgery**  **A. Medications**   - **Stop Blood Thinners:** Avoid aspirin, ibuprofen, or supplements like fish oil for **7 days** before surgery. Your doctor will confirm which medications to pause. - **Continue Essential Medications:** Since you have no history of diabetes or high blood pressure, follow your doctor’s advice if you take any regular medications.   **B. Fasting (No Food/Drink)**   - **No food or drink** after **midnight** the night before surgery. This includes water, gum, and mints. Fasting prevents complications during anesthesia.   **C. Hygiene**   - Shower with antibacterial soap the night before and the morning of surgery. - Do not wear lotions, perfumes, nail polish, or jewelry. - Wear loose, comfortable clothing to the hospital.   **D. Arrange Support**   - Bring your ID and insurance card. - Arrange for a responsible adult to drive you home and stay with you for **at least 24 hours** after surgery.   **3. Day of Surgery**  **A. Arrival**   - Arrive at the hospital by **6:00 AM** (confirm exact time with your surgical team). - Bring glasses (no contact lenses) and any necessary medical documents.   **B. Pre-Surgery Steps**   - Nurses will check your blood pressure, heart rate, and temperature. - An IV (small tube in your arm) will provide fluids and anesthesia. - Your surgeon and anesthesia team will review the plan with you.   **C. During Surgery**   - The surgery typically takes **1–2 hours**. - The team will monitor you closely throughout.   **4. After Surgery**  **A. Recovery Room**   - You’ll wake up here. You may feel sleepy, nauseous, or have a sore throat (from the breathing tube). - Nurses will manage your pain with medication.   **B. Going Home**   - **Rest:** Avoid driving, alcohol, and decision-making for 24 hours. - **Diet:** Start with bland, low-fat foods (e.g., toast, rice, broth). Avoid greasy or spicy foods for 2–4 weeks. - **Activity:**   - Walk gently daily to prevent blood clots.   - Avoid heavy lifting (over 10 lbs) or strenuous exercise for **4–6 weeks**. - **Wound Care:**   - Keep incisions clean and dry. Shower after 48 hours; pat the area gently.   - Watch for redness, swelling, or fluid around the incisions.   **C. Common Side Effects**   - Mild shoulder pain (from gas used during surgery; it will resolve in 1–2 days). - Bloating or mild discomfort in the abdomen.   **D. Warning Signs (Call Your Doctor Immediately If):**   - Fever over 101°F (38.3°C). - Severe or worsening belly pain. - Yellowing of skin/eyes, dark urine, or clay-colored stools. - Vomiting that won’t stop.   **5. Risks and Complications**  While rare, risks include:   - Bleeding, infection, or blood clots. - Injury to nearby organs (e.g., liver, intestines). - Bile leakage (may require further treatment).   **6. Follow-Up**   - **First Appointment:** Schedule within **7–10 days** to check your incisions and recovery. - **Return to Work:** Most patients resume desk work (like accounting) in **1–2 weeks**, depending on recovery.   **Emergency Contact:** [Insert Hospital/Surgeon’s Phone Number]  **Your health and comfort are our priority. Follow these steps to ensure a smooth recovery!**  **Diet Tips After Surgery:**   - Gradually reintroduce fats over 4–6 weeks. Your body will adjust to digesting without a gallbladder. - Eat smaller, frequent meals to avoid discomfort.   **Note:** Genetics (family history of gallbladder stones) may have contributed to your condition. Maintain a healthy, low-fat diet long-term to support digestion.  Let your care team know if you have questions or concerns! |
| 4.Case information: Patients scheduled for transcatheter arterial chemoembolization (TACE) |
| Answer:  **Comprehensive Preoperative Education Guide for Transcatheter Arterial Chemoembolization (TACE)** *Prepared for: Ma M.* *Surgery Type: Transcatheter Arterial Chemoembolization (TACE)* *Surgeon: [Surgeon’s Name]* *Date of Procedure: [Date]*  **1. Overview of Your Procedure**  **Goal:** To explain TACE in simple terms.   - **What is TACE?** TACE is a minimally invasive treatment for liver cancer. Doctors will deliver chemotherapy directly to the tumor and block its blood supply to shrink it. - **How It Works:**   - A thin tube (catheter) is inserted into a blood vessel in your groin and guided to the liver.   - Chemotherapy drugs are injected into the tumor, followed by tiny particles to block its blood flow. - **Expected Benefits:**   - Slow or stop tumor growth.   - Reduce symptoms like pain and fatigue. - **Length of Procedure:**   - Approximately 1–2 hours.   **2. Preparing for the Procedure**  **A. Health Check and Medications**   - **Share with Your Care Team:**   - Hepatitis B history and liver cirrhosis.   - All current medications (including herbal supplements). - **Important Instructions:**   - **Start Antiviral Medication:** Begin taking prescribed antiviral drugs (for Hepatitis B) immediately.   - **Stop Blood Thinners (if any):** Avoid aspirin, ibuprofen, or blood thinners **[X days]** before the procedure (your doctor will confirm).   - **Continue Essential Medications:** Take approved medications (e.g., for blood pressure) with a small sip of water on the morning of TACE.   **B. Fasting Instructions**   - **No food or drinks** (including water, gum, or candy) after **[time]** the night before the procedure. - **Why?** To prevent complications during sedation.   **C. Day Before TACE**   - **Shower:** Use antibacterial soap (provided by the hospital). - **Clothing:** Wear loose, comfortable clothes. - **Arrange Transportation:** You **cannot drive yourself home** after sedation.   **3. What to Expect on the Day of TACE**  **A. Arrival**   - Arrive at the hospital by **[time]** at **[location]**. - Bring:   - ID, insurance card, medication list.   - Comfort items (e.g., glasses, hearing aids). **Do not wear jewelry.**   **B. Before the Procedure**   - Change into a hospital gown. - Nurses will check your blood pressure, heart rate, and oxygen levels. - An IV line will be placed for fluids and sedation.   **C. During TACE**   - You will receive sedation to help you relax (you may be awake or lightly asleep). - A local anesthetic will numb the groin area before inserting the catheter. - Family will wait in a designated area; updates will be provided.   **4. Risks and Possible Complications**  While rare, risks include:   - **Post-Embolization Syndrome:** Temporary fever, nausea, or pain (common and manageable). - **Bleeding or Infection** at the groin site. - **Liver Function Changes** (due to your existing cirrhosis). - Allergic reactions to contrast dye (rare).   **When to Call Your Doctor:**   - Fever >101°F, severe abdominal pain, yellowing skin/eyes (jaundice), or bleeding/swelling at the groin site.   **5. Recovery After TACE**  **A. Immediate Recovery (1–2 Days)**   - You’ll rest in a recovery room for 2–4 hours. - Pressure will be applied to the groin to prevent bleeding. - Pain/nausea medication will be given as needed.   **B. At-Home Care**   - **Activity Restrictions:**   - Avoid heavy lifting, exercise, or prolonged standing for **3–5 days**.   - Walk gently to prevent blood clots. - **Wound Care:**   - Keep the groin site clean and dry for 24 hours.   - Watch for redness, swelling, or discharge. - **Diet:**   - Eat small, frequent meals to support your liver (focus on protein and vegetables).   - Avoid alcohol and limit salty foods (to prevent fluid buildup).   **C. Managing Side Effects**   - **Fatigue/Nausea:** Rest and drink fluids. These usually improve in 3–7 days. - **Mild Pain:** Use prescribed painkillers or acetaminophen (avoid ibuprofen).   **D. Follow-Up**   - Blood tests and imaging (CT/MRI) in **[X weeks]** to assess tumor response. - Continue antiviral therapy for Hepatitis B and regular liver checkups.   **6. Your Questions**  Write down any questions here:  **Contact Information**   - Surgeon’s office: [Phone] - Hepatology Team: [Phone] - Emergency: 911 or go to the nearest ER.   **We’re here to support you throughout your treatment. Call us with any concerns!**  *This guide is tailored to your condition. Always follow your care team’s specific instructions.*  **Key Reminders for Ma M.:**   - Start antiviral medication ASAP to control Hepatitis B. - Report worsening fatigue, abdominal swelling, or confusion (signs of liver stress). - Attend all follow-ups to monitor your liver health and tumor response.   **Stay hopeful—TACE has helped many patients manage liver cancer effectively.** |
| 5.Case information: Patients scheduled for appendectomy |
| Answer:  **Comprehensive Preoperative Education Guide for Appendectomy** *Preparing Chen M. for a Safe and Successful Surgery*  **1. Understanding Your Surgery**   - **What is an appendectomy?**   - A surgery to **remove your appendix**, a small organ in the lower right abdomen.   - **Why is it needed?** Your appendix is swollen and infected (acute appendicitis). Removing it prevents rupture, which can cause serious complications.   - **Who will perform it?** Your surgeon, *[Dr. Name]* (to be filled in), and their team.   **2. Preparing for Surgery**  **Medications**   - **Stop blood thinners** (e.g., aspirin, ibuprofen) 3 days before surgery, as they can increase bleeding. - **Continue approved medications** (if any) with a small sip of water on the morning of surgery.   **Fasting**   - **No food, drinks, gum, or water** after **midnight the night before surgery**.   **Hygiene**   - **Shower the night before or morning of surgery** using antibacterial soap (if provided). - **Remove nail polish, jewelry, and piercings**.   **What to Bring**   - ID, insurance card, and loose, comfortable clothing for discharge. - Leave valuables at home.   **Lifestyle Adjustments**   - **Avoid alcohol** for 24 hours before surgery. - **Arrange transportation**: You cannot drive for 24 hours after surgery.   **3. Day of Surgery**   - **Arrival**: Arrive at *[time]* at *[hospital location]*. - **Check-In**: Nurses will confirm your details, check your vitals, and place an IV for fluids/medication. - **Anesthesia**: You’ll receive **general anesthesia** to sleep pain-free during the procedure. - **Procedure**: The surgery will take **about 1 hour** and is likely done laparoscopically (small incisions).   **4. After Surgery**  **Recovery Room**   - You’ll wake up in a recovery area. Nurses will monitor your pain, vital signs, and incisions.   **Pain Management**   - Prescribed pain medication (e.g., acetaminophen) will help. Report severe pain promptly.   **Activity**   - **Rest for 1–2 days**. Avoid heavy lifting, sports, or driving for **1–2 weeks**. - Walk gently the same day to prevent blood clots.   **Diet**   - Start with **clear fluids** (water, broth) and progress to bland foods (toast, rice) as tolerated.   **Wound Care**   - Keep incisions clean and dry. Avoid baths; take quick showers after 48 hours.   **5. Risks and Complications**  While rare, possible risks include:   - **Infection**: Redness, swelling, or pus at the incision. - **Bleeding**: Unexpected bleeding or bruising. - **Blood clots**: Calf pain, swelling, or trouble breathing. **Call your doctor immediately if you notice these signs.**   **6. Emotional Support**   - **Anxiety is normal**. Talk to your care team about any fears. - **Arrange a friend/family member** to stay with you for 24 hours after discharge.   **7. Follow-Up Care**   - **First appointment**: *[Date/time]* to check healing and remove stitches (if needed). - **Call your doctor if**:   - Fever over 101°F (38.3°C).   - Severe pain or vomiting.   - Incision opens or leaks fluid.   **Pre-Op Checklist**   - □ Fasting from midnight. - □ Shower with antibacterial soap. - □ Bring ID, insurance card, and loose clothing. - □ Arrange a ride home.   **Contact Information**   - Surgeon: *[Name, phone]* - Hospital: *[Phone]* - Emergency line: *[Phone]*   **Follow your surgical team’s specific instructions closely.** *Wishing you a smooth surgery and recovery!* 🌟  **Note for Chen M.**: As a university student, inform your school about your surgery and arrange for academic flexibility during recovery. Avoid strenuous activities until cleared by your surgeon. |
| 6.Case information: Patients scheduled for transurethral resection of the prostate (TURP) |
| Answer:  **Comprehensive Preoperative Education Guide for Mr. Meng M.**  **1. Understanding Your Surgery**  **Procedure Name:** Transurethral Resection of the Prostate (TURP) **Purpose of Surgery:** To remove part of your enlarged prostate gland to improve urine flow and relieve symptoms like difficulty urinating. **Surgeon:** [Insert Surgeon’s Name] **Date & Location:** [Insert Surgery Date], [Insert Hospital Name].  **2. Preparing for Surgery**  **A. Medications**   - **Continue Essential Medications:**   - Keep taking your **blood pressure medications** as usual (they are important for your safety during surgery).   - **Do NOT stop any prescribed medications unless your doctor advises you to.** - **Stop Blood Thinners:** Avoid aspirin, ibuprofen, or herbal supplements (like ginkgo or garlic) **7 days before surgery** (your doctor will confirm the exact date). - **Diabetes Management:** Not applicable (you have no history of diabetes).   **B. Fasting**   - **No Food or Drink:** Do **not** eat or drink (including water) after **12:00 AM (midnight)** the night before surgery.   **C. Hygiene**   - **Shower:** Use antibacterial soap the night before and morning of surgery. Focus on cleaning the genital area gently. - **No Jewelry/Cosmetics:** Remove rings, watches, nail polish, and dentures before arriving at the hospital.   **D. Arrange Support**   - **Transportation:** You **cannot drive yourself home** after surgery. Ask a family member or friend to take you. - **Caregiver:** Have someone stay with you for at least **24 hours after surgery** to assist you.   **E. Consent Form**   - You will sign a consent form before surgery. Ask your surgeon any questions you have about the procedure.   **3. What to Expect on Surgery Day**  **A. Check-In**   - Arrive at the hospital at **[insert time]**. Bring your ID, insurance card, and a list of your medications.   **B. Preoperative Prep**   - Change into a hospital gown. - A nurse will check your blood pressure, heart rate, and oxygen levels. - An IV line will be placed in your arm to give fluids and medications.   **C. Anesthesia**   - **Type:** You will receive **spinal anesthesia** (numbing medication injected near your spine) or **general anesthesia** (medication to make you sleep). Your anesthesiologist will decide the best option for you. - **During Surgery:** You will feel no pain. The surgery takes about **1–2 hours**.   **D. During Surgery**   - A thin tube with a camera (resectoscope) will be inserted through your urethra (the tube that carries urine out of your body) to remove excess prostate tissue. - A catheter (flexible tube) will be placed in your bladder to drain urine after surgery.   **4. Recovery After Surgery**  **A. Immediate Post-Op**   - You will wake up in the recovery room. Nurses will monitor you for 1–2 hours. - You may feel drowsy, have a sore throat (if a breathing tube was used), or bladder discomfort.   **B. Catheter Care**   - The catheter will stay in place for **1–3 days** to help your bladder heal. - You may feel the urge to urinate—this is normal. The catheter will drain urine automatically.   **C. Pain Management**   - Mild pain or burning during urination is common. Your doctor will prescribe pain relievers. - Drink plenty of water to flush your bladder and reduce discomfort.   **D. Activity Restrictions**   - Rest for **1–2 days** after surgery. Avoid heavy lifting, driving, or strenuous activity for **4–6 weeks**. - Walk short distances at home to prevent blood clots.   **E. Diet**   - Start with light meals (broth, toast) after surgery. Gradually return to your normal diet.   **5. Risks & Complications***  While rare, possible risks include:   - **Bleeding:** Small amounts of blood in urine are normal for a few weeks. - **Infection:** Signs include fever, chills, or cloudy/painful urine. - **Temporary Urinary Symptoms:** Burning, urgency, or leakage may occur. - **Retrograde Ejaculation** (semen flows backward into the bladder during ejaculation—harmless but may affect fertility).   **Call Your Doctor Immediately If You Experience:**   - Fever over 101°F (38.3°C). - Severe pain, heavy bleeding, or inability to urinate after catheter removal. - Chest pain or trouble breathing.   **6. Follow-Up Care**   - **Catheter Removal:** The catheter will be removed in the hospital or at a follow-up visit. - **First Appointment:** See your surgeon **1–2 weeks after surgery** to check healing. - **Long-Term Care:** Practice pelvic floor exercises (if advised) to improve bladder control.   **7. Checklist Before Surgery**   - [ ] Continue blood pressure medications. - [ ] Stop blood thinners/herbal supplements 7 days before surgery. - [ ] Fast after midnight the night before surgery. - [ ] Arrange transportation and a caregiver. - [ ] Pack ID, insurance card, and medication list.   **Questions? Contact Your Surgical Team:**   - Surgeon: **[Phone]** - Nurse: **[Phone]** - Hospital: **[Phone]**   **We’re here to ensure your surgery and recovery go smoothly! Follow these steps and reach out with any concerns.**  *This guide is tailored to your medical history (hypertension, enlarged prostate, no diabetes/allergies).* |

Table A.4: Accuracy - a 5-point Likert scale

| score | description | requirement details |
| --- | --- | --- |
| 5 | Extremely accurate | • All medical content (such as surgical procedures, risks, and rehabilitation plans) is in accordance with the latest guidelines/consensus.  • The data sources are clearly stated  • There are no misleading statements or outdated information. |
| 4 | Basically accurate, with a slight deviation. | • The core content is accurate, but there are non-critical deviations in secondary details (such as drug dosage and recovery time);  • No references were cited, but the content conforms to clinical norms. |
| 3 | Partially correct, needs correction | • The key content contains 1-2 errors (such as incorrect descriptions of anesthesia methods or prosthesis lifespan);  • Some information lacks evidence support (such as "able to run 3 days after surgery"); The core content is accurate, but there are non-critical deviations in secondary details (such as drug dosage, rehabilitation time);  • No references were cited but the content conforms to clinical norms. |
| 2 | Clearly incorrect, poses a safety risk | • There are significant medical errors (such as underestimating the risk of thrombosis, incorrect fasting times);  • This may mislead patients or increase the risk of complications. |
| 1 | Absolutely not credible | • It significantly deviates from medical consensus (such as "no need for rehabilitation training");  • Contains false information or unverified statements. |

Table A.5: Completeness - a 5-point Likert scale

| score | description | requirement details |
| --- | --- | --- |
| 5 | Extremely comprehensive, covering all key aspects in a thorough manner. | It covers all the "must-have" contents, such as preoperative preparations, surgical procedures, risks, rehabilitation plans, and common questions. No important information has been omitted. |
| 4 | Almost complete, the main content is covered, but there are a few details missing. | Only a few minor contents or details are omitted, but the core information is fully presented. |
| 3 | Generally, there are some flaws or omissions, which affect the understanding to some extent. | The key content is incomplete or insufficient, such as the absence of risk warnings or recovery information, which affects the comprehensive understanding of the surgery. |
| 2 | The structure is incomplete and crucial aspects are clearly omitted. | The main content blocks are missing, such as those related to post-operative rehabilitation or potential risks. The content is seriously insufficient. |
| 1 | Extremely incomplete, with significant information loss or a serious deviation from the topic. | Providing only a small amount of information is basically not sufficient to help patients understand the preoperative preparations and the surgical procedures. |

Table A.6: Subgroup Analysis of PEM Quality Across Surgery Types

| Dimension & Model | Mean | SD | Median | Variance | Min | Max | Friedman Test (χ²(5), *P*) |
| --- | --- | --- | --- | --- | --- | --- | --- |
| **Accuracy** |  |  |  |  |  |  | χ² = 8.18,  *P* = 0.146 |
| CABG | 4.00 | 0.00 | 4.00 | 0.00 | 4.00 | 4.00 |  |
| TKA | 3.83 | 0.41 | 4.00 | 0.17 | 3.00 | 4.00 |  |
| LC | 3.83 | 0.41 | 4.00 | 0.17 | 3.00 | 4.00 |  |
| TACE | 3.83 | 0.41 | 4.00 | 0.17 | 3.00 | 4.00 |  |
| APPX | 3.50 | 0.55 | 3.50 | 0.30 | 3.00 | 4.00 |  |
| TURP | 4.00 | 0.00 | 4.00 | 0.00 | 4.00 | 4.00 |  |
| **Completeness** |  |  |  |  |  |  | χ² = 10.95,  *P* = 0.052 |
| CABG | 3.17 | 0.75 | 3.00 | 0.58 | 2.00 | 4.00 |  |
| TKA | 3.00 | 0.55 | 3.00 | 0.30 | 2.00 | 4.00 |  |
| LC | 3.67 | 0.52 | 4.00 | 0.27 | 3.00 | 4.00 |  |
| TACE | 3.67 | 0.52 | 4.00 | 0.27 | 3.00 | 4.00 |  |
| APPX | 3.50 | 0.55 | 3.50 | 0.30 | 3.00 | 4.00 |  |
| TURP | 3.67 | 0.52 | 4.00 | 0.27 | 3.00 | 4.00 |  |
| **Understandability** |  |  |  |  |  |  | χ² = 11.47,  *P* = 0.043 |
| CABG | 77.348 | 6.746 | 72.727 | 45.501 | 72.727 | 90.909 |  |
| TKA | 82.583 | 4.609 | 81.818 | 21.246 | 77.777 | 90.909 |  |
| LC | 83.080 | 7.373 | 81.818 | 54.359 | 72.727 | 90.909 |  |
| TACE | 81.818 | 1.488 | 81.818 | 2.214 | 77.777 | 81.818 |  |
| APPX | 83.743 | 3.737 | 81.818 | 13.966 | 81.818 | 90.909 |  |
| TURP | 86.868 | 5.378 | 90.909 | 28.928 | 77.777 | 90.909 |  |
| **Actionability** |  |  |  |  |  |  | χ² = 7.5,  *P* = 0.18 |
| CABG | 76.67 | 2.58 | 75.00 | 6.67 | 75.00 | 80.00 |  |
| TKA | 71.67 | 10.80 | 75.00 | 116.67 | 50.00 | 80.00 |  |
| LC | 75.00 | 0.00 | 75.00 | 0.00 | 75.00 | 75.00 |  |
| TACE | 75.00 | 0.00 | 75.00 | 0.00 | 75.00 | 75.00 |  |
| APPX | 75.83 | 2.04 | 75.00 | 4.17 | 75.00 | 80.00 |  |
| TURP | 77.50 | 2.74 | 77.50 | 7.50 | 75.00 | 80.00 |  |
| **Suitability** |  |  |  |  |  |  | χ² = 5.614,  *P* = 0.345 |
| CABG | 76.67 | 2.58 | 75.00 | 6.67 | 75.00 | 80.00 |  |
| TKA | 72.50 | 11.23 | 75.00 | 126.04 | 50.00 | 80.00 |  |
| LC | 75.00 | 0.00 | 75.00 | 0.00 | 75.00 | 75.00 |  |
| TACE | 75.00 | 0.00 | 75.00 | 0.00 | 75.00 | 75.00 |  |
| APPX | 75.83 | 1.89 | 75.00 | 3.58 | 75.00 | 80.00 |  |
| TURP | 77.50 | 2.74 | 75.00 | 7.50 | 75.00 | 80.00 |  |
| **Readability** |  |  |  |  |  |  |  |
| *FKRE* |  |  |  |  |  |  | χ² = 2.512,  *P* = 0.7747 |
| CABG | 61.0667 | 11.0558 | 64.70 | 122.2300 | 39.9 | 69.9 |  |
| TKA | 60.5167 | 6.4851 | 61.45 | 42.0567 | 50.5 | 67.7 |  |
| LC | 58.9333 | 9.6053 | 61.95 | 92.2627 | 41.3 | 69.5 |  |
| TACE | 59.6833 | 9.3752 | 59.60 | 87.8947 | 46.8 | 71.5 |  |
| APPX | 57.2667 | 13.5649 | 61.30 | 183.9927 | 31.6 | 66.9 |  |
| TURP | 59.1333 | 6.6212 | 56.00 | 43.8407 | 53.6 | 69.0 |  |
| *FKGL* |  |  |  |  |  |  | χ² = 2.3077,  *P* = 0.8051 |
| CABG | 8.7333 | 3.8966 | 7.30 | 15.1830 | 6.6 | 16.8 |  |
| TKA | 8.4167 | 2.3168 | 7.25 | 5.3670 | 6.8 | 12.3 |  |
| LC | 8.5000 | 3.1654 | 7.60 | 10.0200 | 6.3 | 15.2 |  |
| TACE | 8.6500 | 3.4687 | 7.45 | 12.0320 | 6.4 | 14.4 |  |
| APPX | 8.4000 | 4.4996 | 7.35 | 20.2460 | 6.3 | 18.9 |  |
| TURP | 8.5167 | 1.0991 | 7.75 | 1.2087 | 6.9 | 9.5 |  |


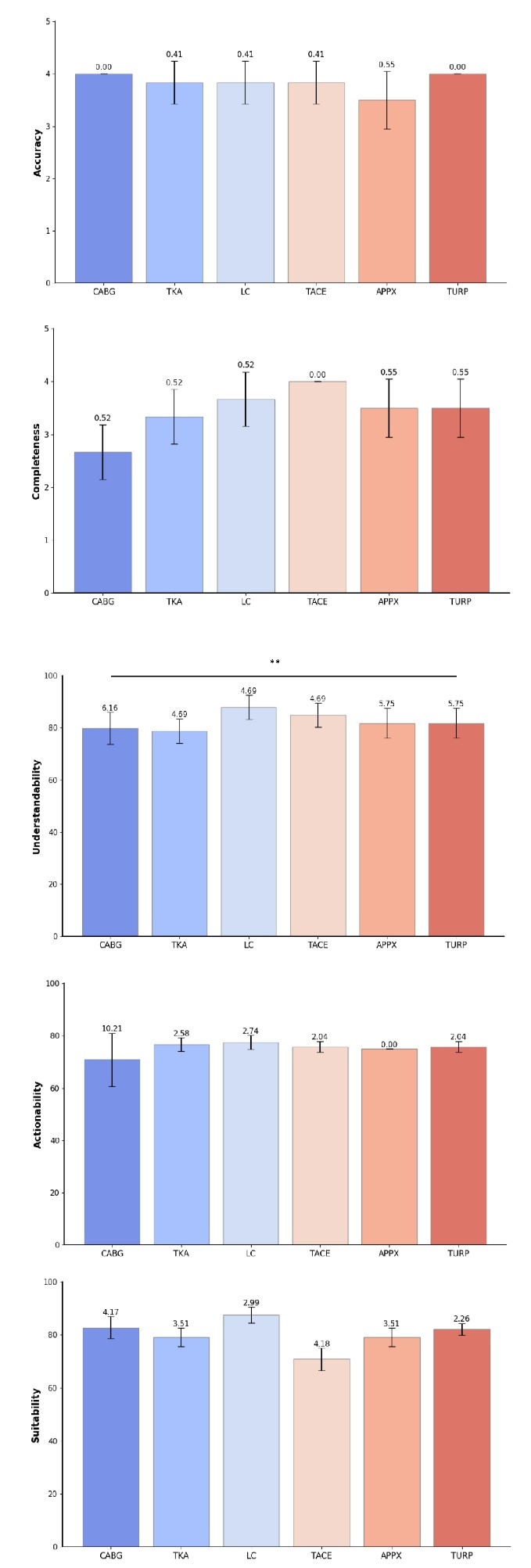


Figure A.1: Comparison of PEM Scores Across Different Surgery Types.
